# LA VISION^©^: A Structured Approach to Lip Analysis and Aesthetic Treatment with Hyaluronic Acid Fillers

**DOI:** 10.1007/s00266-026-05998-7

**Published:** 2026-07-01

**Authors:** Andrea F. Armenti

**Affiliations:** LA VISION Training Institute, Via Magenta, 5, 00185 Rome, Italy

**Keywords:** Lip enhancement, Injection planning, Algorithm, Filler, Hyaluronic acid, Facial aesthetics

## Abstract

**Background:**

Hyaluronic acid (HA) filler-based aesthetic improvement of the lips is popular with patients. However, it is complicated by great diversity in individual desires and underlying morphology. Many injection techniques have been published, but there is a need to integrate these within a methodological framework for codified lip analysis and treatment planning. The LA VISION Method provides such a framework. This paper describes the methodology, explains the coding language, and illustrates its clinical application.

**Methods:**

The LA VISION Method is composed of three consecutive and interdependent phases: (1) MAP, which establishes the morpho-geometric structure of the lips through anthropometric lines and defined topographic mapping, using a novel coding system; (2) ASSESS, which collects and analyzes patient-specific information, such as aesthetic desires, detailed lip morphology, and facial proportions, to define their current and ideal lip structure; and (3) PLAN, which translates the assessment into a tailored logic-based injection strategy to convert lip structure towards the defined ideal and individual shape.

**Results:**

An initial cohort of 400 patients (mean age: 41.0 years; 53.2% female) was treated using a mean filler volume of 1.00 ± 0.01 mL (first session). Adverse events were typically localized and mild in severity; no major complications were observed. Patient-reported outcomes were positive.

**Conclusions:**

The LA VISION Method provides a systematic, logical, clinically usable approach with a reproducible decision logic for lip analysis and injection planning using HA fillers. It also creates a common language for clinician training, documentation, communication with peers and patients, and future digital integration. Further studies are warranted.

**Level of Evidence IV:**

This journal requires that authors assign a level of evidence to each article. For a full description of these Evidence-Based Medicine ratings, please refer to the Table of Contents or the online Instructions to Authors www.springer.com/00266.

**Supplementary Information:**

The online version contains supplementary material available at 10.1007/s00266-026-05998-7.

## Important Points

- There is a need to integrate injection techniques for nonsurgical lip enhancement within a methodological framework for codified analysis and treatment planning.
- The LA VISION Method provides a systematic and logical approach with a reproducible decision logic for lip analysis and injection planning based on hyaluronic acid fillers.
- LA VISION is composed of three consecutive and interdependent phases, known as MAP (which establishes the morpho-geometric structure of the lips), ASSESS (which collects and analyzes patient-specific information to define their current and ideal lip structure), and PLAN (which translates the assessment into a tailored injection strategy).
- This paper describes the methodology, explains the proposed coding language, illustrates its clinical application through representative case material, and documents a favorable safety profile and positive patient-reported outcomes in a longitudinal cohort of 400 subjects.

## Introduction

The lips and mouth are a central feature of the human face. They serve not only functional roles in speaking and eating but also play a major part in facial expression, emotional signaling, and perceived attractiveness [1, 2]. Their contribution to overall harmony is so critical that even subtle modifications in shape, volume, or contour can substantially alter the aesthetic perception of the entire face [3–5].

Furthermore, the lips remain a dominant symbol of personal identity around the world, particularly among women. It is no coincidence that the cosmetics industry has long centered its marketing on lip-focused products, and the recent influence of social media has further amplified the perception of the lips as a key element of female beauty [4, 6].

As a result, demand for aesthetic enhancement continues to grow, and lip improvements are now among the most commonly performed aesthetic procedures worldwide [7]. Despite this popularity, many clinical approaches remain primarily technique-driven, without a standardized framework that explicitly integrates patient individuality, aesthetic analysis, and procedural planning.

However, assessment and planning should be considered as particularly important when treating the lips, given that their morphology is so profoundly individual. In addition, aesthetic ideals can differ greatly according to personality, cultural background, and emotional identity, while perceptions of beauty may vary significantly with age, gender, and ethnicity [8–13]. Some patients seek harmony and subtle correction; others desire transformation and individual expression. Such diversity in biology and expectation cannot be treated based on overly simplified techniques or passing filler trends.

Nonetheless, although key anatomical variables—like fullness, upper-to-lower lip ratio, lateral projection, lip shape, and proportion—are not static, they are widely observed across populations. As clinicians, we need to be in full control of assessment and planning so that we respect both the uniqueness of each patient and the shared structural dimensions that underlie lip analysis.

The present work introduces the Lip Algorithmic (LA) VISION Method, a structured, algorithmic approach to lip analysis and injection planning based on hyaluronic acid (HA) fillers. The aim of this paper is to describe the methodology, explain the coding language, illustrate its clinical application through real-world case presentations, and assess safety and patient satisfaction in an initial cohort of 400 subjects—thus supporting a new paradigm for aesthetic lip enhancement.

## Materials and Methods

### Overview

The LA VISION Method provides a structured, reproducible framework that integrates patient desires, objective facial analysis, individualized anatomical considerations, lip shape codification, tissue layer-based stratification, and strategic injection planning into a unified algorithmic process.

It is composed of three consecutive and interdependent phases:

1. *MAP*: Establishes the morpho-geometric structure of the lips through anthropometric lines and defined topographic mapping, using a novel coding system;
2. *ASSESS*: Collects and analyzes patient-specific information, such as aesthetic desires, detailed lip morphology, and facial proportion, to define their current and ideal lip structure; and
3. *PLAN*: Translates the assessment into a tailored injection strategy to convert lip structure toward the defined ideal and individual shape.

**Fig. 1:**
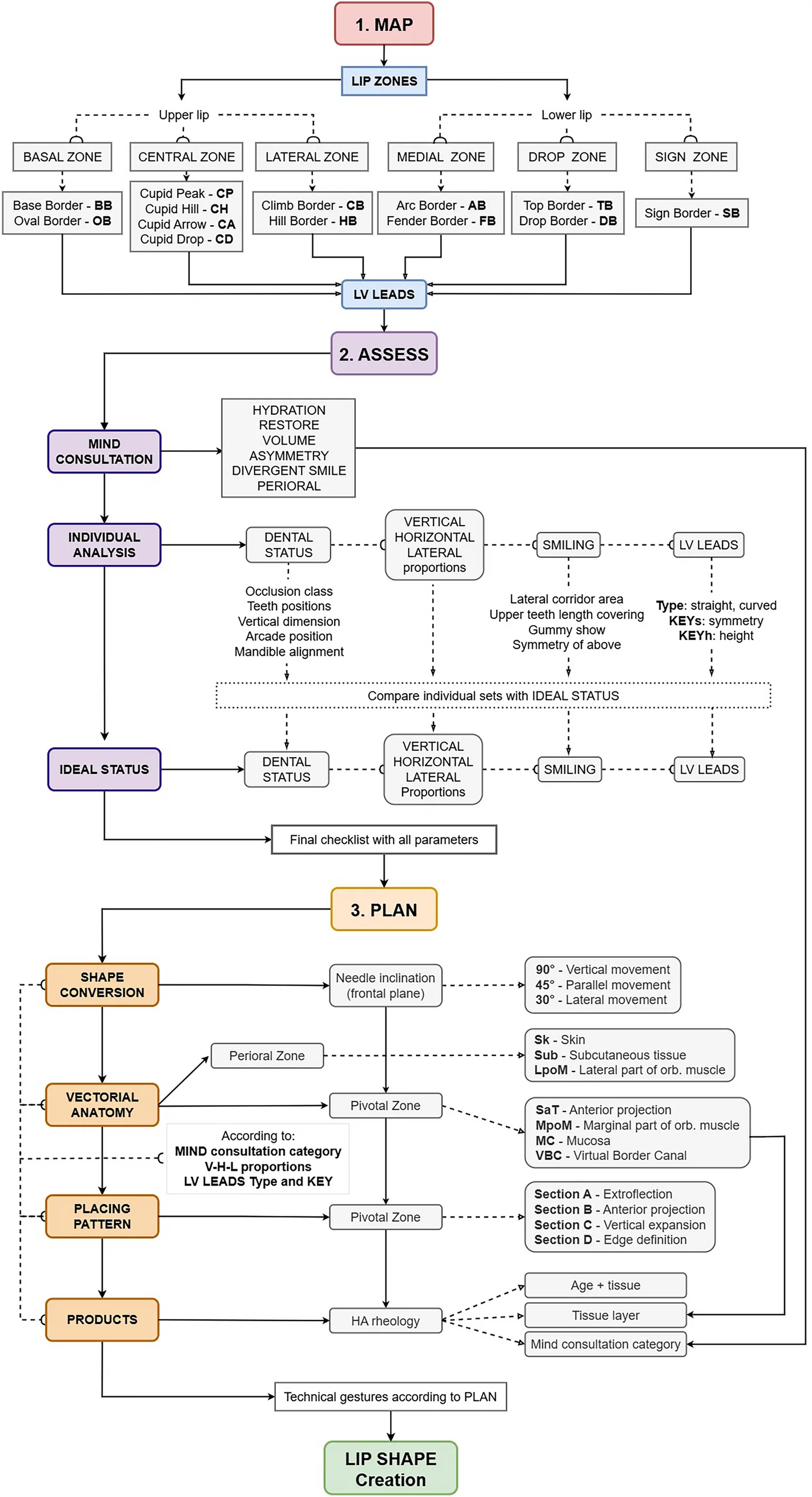
LA VISION Method: summary. *HA* hyaluronic acid, *LV LEADS* Lip Visual Linear Elements for Aesthetic Design and Symmetry, *orb.* orbicularis

These phases are summarized in Fig. 1 and described in turn below.

### MAP: Geometric Coding of Lip Shape

**Fig. 2:**
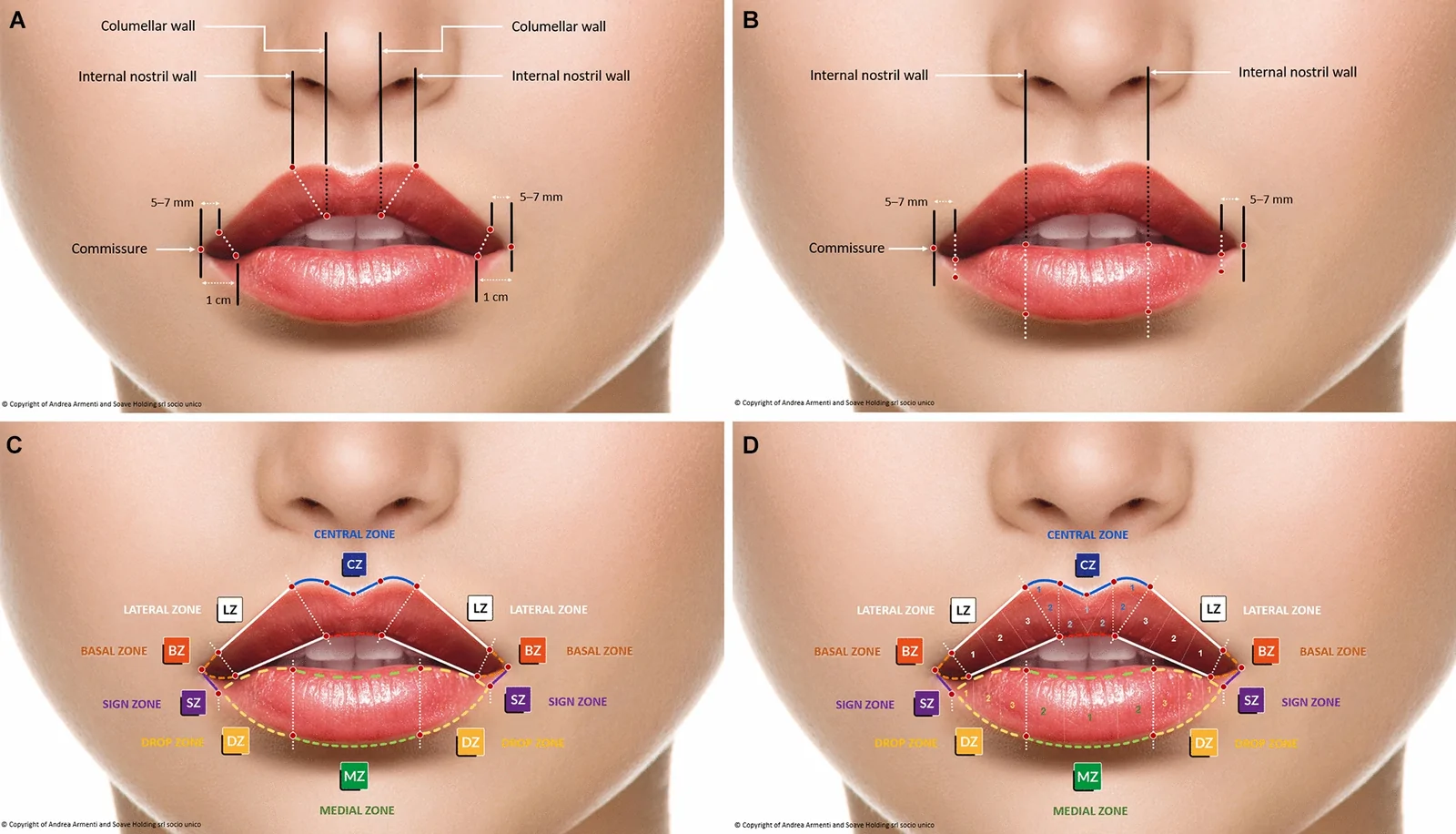
Defining the six zones of the upper and lower lip. On the upper lip, key points (demarcated with red circles) are at the oral commissure, a point 5–7 mm medial to the commissure along the upper edge, a point 1 cm medial to the commissure along the lower edge, a point vertically below the internal nostril wall on the upper edge, and a point vertically below the columellar wall on the lower edge (part **A**). On the lower lip, key points (demarcated with red circles) are the oral commissure, points 5–7 mm medial to the commissure along the upper and lower edges, and points vertically below the internal nostril wall on the upper and lower edges (part **B**). These distances are contextual orientation ranges used to support proportional mapping rather than fixed anatomical measurements or universal ‘safe’ points. Using these points, the upper lip is divided into a Central Zone, flanked on each side by a pair of Lateral Zones and Basal Zones; similarly, the lower lip is divided into a single Medial Zone, flanked on each side by a pair of Drop Zones and Sign Zones (part **C**). For more precise location of exact points, each zone can be further separated into numerical subdivisions (part **D**)

In this first phase, the lips are analyzed according to easily demarcated, fixed anthropometric landmarks and facial reference lines—shown for the upper lip in Fig. 2A and for the lower lip in Fig. 2B. Using these landmarks, the lips can then be divided into six anatomical–functional zones, each representing a unique aspect of aesthetic architecture (Fig. 2C and D). In the upper lip, there is a single Central Zone, flanked on each side by a Lateral Zone and a Basal Zone; similarly, the lower lip is divided into a single Medial Zone, flanked on each side by a Drop Zone and a Sign Zone. The key elements, function, and clinical relevance of each zone are summarized in Table 1.

**Table 1:** Zones of the upper and lower lip

Zone	Key elements	Function	Clinical relevance
*Upper lip*			
Central Zone	Cupid’s bow, vermilion apex, philtral crest	Primary visual focus in frontal view; defines midline projection and harmony	Often the target for Cupid enhancement, projection correction, and central balancing
Lateral Zone	Lateral vermilion curve, smile junction	Defines width and lateral balance of the lip, influences smile width and contour	Important for smoothing asymmetry and supporting lip curvature in smile dynamics
Basal Zone	Nasolabial groove, base of the columella	Structural support zone, connecting upper lip to the nasal and midface anatomy	Often involved in age-related volume loss, influences prolabial angle and nasolabial fold aesthetics
*Lower lip*			
Medial Zone	Central vermilion bulk, chin transition	Core area for lower lip projection and facial balance	Target for central fullness enhancement, balancing upper lip projection
Drop Zone	Curved arc forming the lower lip drop	Controls vertical dimension, particularly in profile view	Crucial for creating definition, avoiding overfilling, and respecting mandibular harmony
Sign Zone	Inferior lateral border, adjacent to chin and jawline	Shapes lateral contour and extension, contributing to the ‘signature’ of lip movement	Sensitive area for expression dynamics; must be handled with caution to avoid distortion during projection procedures

These six zones should not be considered as rigid ‘rules’ but rather as modular structures that are adjustable based on individual gender expression (e.g., sharpness in males versus roundness in females), age dynamics (e.g. volume loss in the Basal Zone or elongation of the Medial Zone), and ethnicity (vermilion prominence, philtrum length, etc) [8–13]. Indeed, typical lip volume and proportions can differ greatly between ethnicities [8], while recent data showed that ideals of philtrum length—and associated incisor show—vary substantially in faces from different ethnic groups (Black, East Asian, Caucasian, Latino, and Middle Eastern) [13].

Each of the LA VISION zones can be analyzed independently, but their synergy determines the overall result. For example, the Central Zone of the upper lip must project harmoniously with the Medial Zone of the lower lip, while overcorrection of the Drop Zone should be avoided as it can lead to unnatural protrusion or a pseudo-class II effect. Meanwhile, proper shaping of the Sign Zone ensures natural lateral convergence and avoids tension build-up at the commissures.

**Fig. 3:**
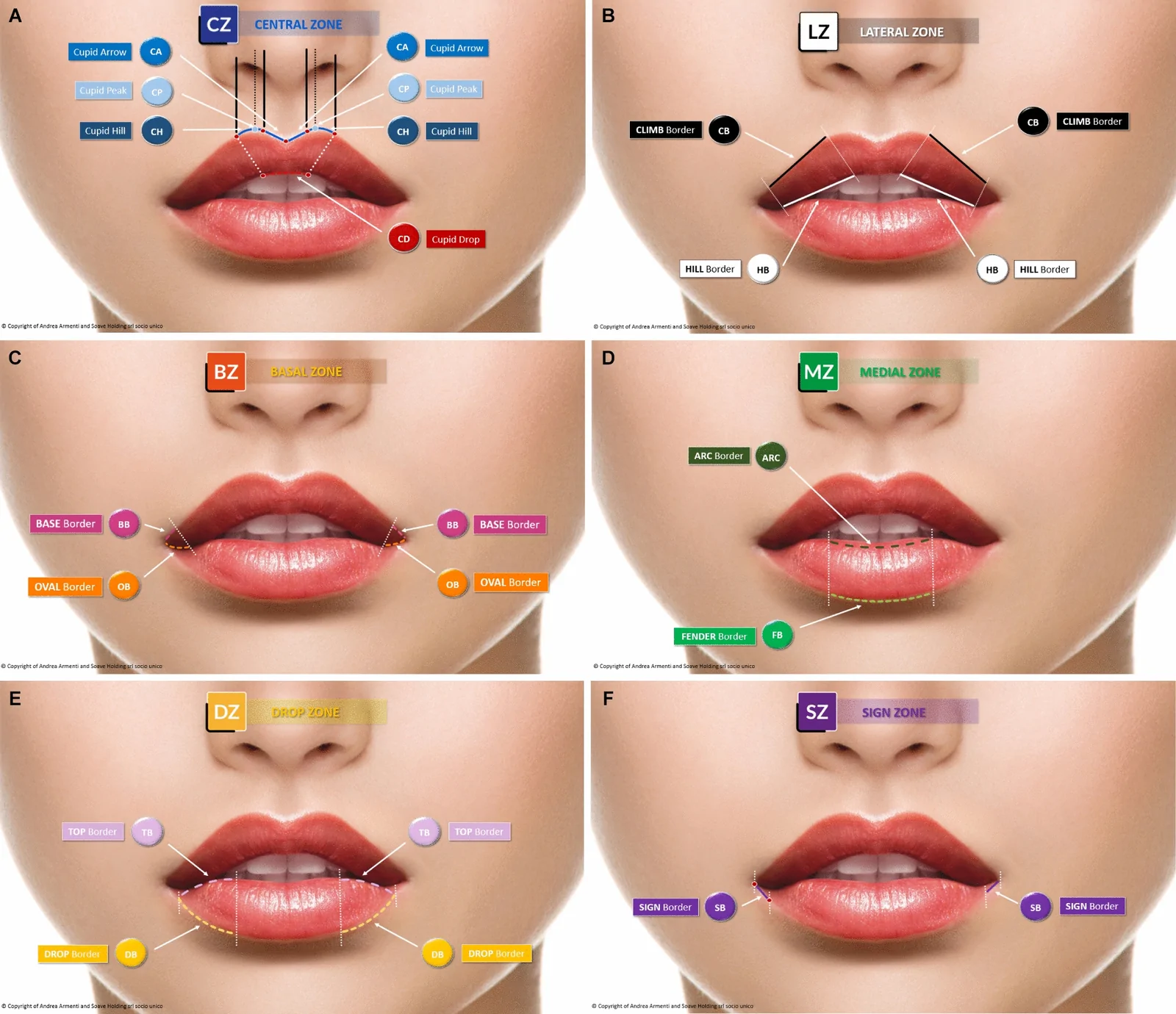
LV LEADS. These are shown for the Central Zone (part **A**), Lateral Zone (part **B**), and Basal Zone (part **C**) of the upper lip; and the Medial Zone (part **D**), Drop Zone (part **E**), and Sign Zone (part **F**) of the lower lip. *LV LEADS* Lip Visual Linear Elements for Aesthetic Design and Symmetry

Within these six zones, 13 visual aesthetic lines and points can be identified—known as ‘Lip Visual Linear Elements for Aesthetic Design and Symmetry’ (LV LEADS). These are shown in Fig. 3. Each one describes a precise element of the lip’s contour. By codifying these elements, the LV LEADS provide a detailed blueprint of lip form.

Specifically, in the upper lip, the Central Zone incorporates four LV LEADS: Cupid Peak^©^ (a point 1 mm lateral to the intersection of the upper edge of the lip with a vertical line from the columellar wall), Cupid Hill^©^ (along the upper edge of the lip from the lateral extremity of the zone to Cupid Peak), Cupid Arrow^©^ (the remaining portion of the upper edge of the lip medial to Cupid Peak), and Cupid Drop^©^ (along the lower edge of the upper lip). The Lateral Zone includes two LV LEADS: Climb Border^©^ (along its upper edge) and Hill Border^©^ (along its lower edge). Similarly, the Basal Zone has two LV LEADS: Base Border^©^ (along its upper edge) and Oval Border^©^ (along its lower edge).

In the lower lip, the Medial Zone includes two LV LEADS: Arc Border^©^ (along its upper edge) and Fender Border^©^ (along its lower edge). The Drop Zone also incorporates two LV LEADS: Top Border^©^ (along its upper edge) and Drop Border^©^ (along its lower edge). Finally, the Sign Zone includes just one of the LV LEADS: the Sign Border^©^ (along its lower edge).

Within the MAP phase, the LV LEADS system functions as a topographic decision framework rather than as an anatomical coordinate system. Spatial references relative to individualized facial landmarks (e.g., oral commissures, midline, nostrils) are used to partition the lips into operational zones relevant to shape construction and planning. These references are intentionally proportional and contextual, allowing the mapping structure to remain consistent in logic across patients, while accommodating individual morphological, cultural, gender, and generational variations.

The terminology for designating individual LV LEADS (‘Cupid Arrow’, ‘Cupid Peak’, etc) is used as an operational descriptive language designed to translate lip features into a structured decision-making framework. These terms are not intended to replace classical anatomical nomenclature but instead function as complementary descriptors that facilitate consistent observation, communication, and planning.

### ASSESS: Clinical Evaluation and Ideal Targeting

This second phase of the LA VISION Method provides an objective approach to evaluate the patient’s individual aesthetic desires, lip morphology, and facial proportions. It is broadly applicable, repeatable, and usable across different levels of clinical experience.

The ASSESS phase incorporates three consecutive elements:

- *Mind consultation:* To understand the patient’s motivations and expectations;
- *Individual analysis:* To examine each of the LV LEADS in terms of shape, symmetry, proportion, and dynamic behavior, as well as lower-third facial proportions in accordance with these; and
- *Ideal status:* To define an ideal version of each of the LV LEADS and of lower-third facial proportions.

The ‘Mind consultation’ categorizes the patient’s desires so that the clinician can align the treatment plan accordingly. Common desires for improvement include: hydration (improving lip texture and moisture, ‘Hydration’); restoration (addressing age-related deficits, ‘Restore’); volume enhancement (increasing lip fullness, ‘Volume’); asymmetry management (balancing uneven lip contours, ‘Asymmetry’); smile correction (modifying a divergent or asymmetric smile, ‘Divergent smile’); and addressing the perioral area (optimizing the perioral frame, ‘Perioral’). Such categorizations are essential because they facilitate conversion of the patient’s wishes into programmed tasks for the clinician to perform.

The ‘Individual analysis’ incorporates four parts that should be completed consecutively: dental status; vertical, horizontal, and lateral proportions; evaluation of LV LEADS; and smile dynamics. These four elements form the basis of patient analysis for all clinicians.

Starting with dental status, key variables include occlusion class, overjet, overbite, teeth malposition, and vertical dimension (Table 2). These can greatly influence the vectorial feasibility of the lip vermilion. In this regard, it is also important to assess the lips in static and dynamic (smiling), based on observing movement during expression and speech (Table 3). Each of these parameters alters lip tension, shape, and functional limits.

**Table 2:** Dental status

Parameters	Clinical situation
Dental class	Class I–II–III
	Overjet (1–2 mm ideal), overbite (open/normal/deep)
Teeth positions	Overjet (1–2 mm ideal)
	Overbite (open/normal/deep)
Vertical dimension^a^ and its impact on lip support	Normal
	Short
	Long
Mandible alignment	In line
	(Right–left) lateralization

^a^Distance between the upper and lower jaw when the teeth are in contact

**Table 3:** Lip status

Parameters	Clinical situation
Lips in static	Upper lip vermilion eversion (fully inverted, partially inverted, everted)
	Lower lip vermilion eversion (fully inverted, partially inverted, everted—flat or round)
Lips in dynamic	Lateral black corridor shape (wide or short)
	Upper lip—lower margin (no gingival display over cuspids)
	Lower lip—upper margin (no covering upper incisor dental arcade)

In addition, facial proportions should be examined to ensure harmony between the lips and surrounding features. The most relevant assessments of vertical, horizontal, and lateral proportions are summarized in Supplementary Table 1. With regard to vertical proportions, previous work has suggested that four parameters may be particularly important [14]: philtrum height, upper vermilion height, the ratio between philtrum and upper vermilion height, and the ratio between upper and lower vermilion height. Key lateral assessments are summarized in Supplementary Table 1 and are covered in detail in Supplementary Material 1—including vermilion properties and profile lines.

Evaluation of each of the 13 LV LEADS should be based on three criteria (Table 4). First, there are the ‘Types’, which define the geometric form of the line (e.g., curved, straight, or mixed). Second, there are the ‘KEYs’, which examine functional morphometric properties (e.g., symmetry, projection direction, and length), and third, there are the ‘KEYh’, which assess any height-related asymmetries or vertical deviations (Supplementary Material 2). These elements provide a codified approach to examining complex anatomical variations and planning targeted interventions.

**Table 4:** Individual analysis of LV LEADS and typical ideals

Zone	LV LEADS	Individual analysis			Typical ideal
		Types	KEYs	KEYh	
*Upper lip*					
Central Zone	Cupid Hill	Curved	*N/A*	*N/A*	Triangular upward elevation to define Cupid’s bow
		Triangular			
	Cupid Peak	*N/A*	Short distance from columella	Peak height (high or low)	At base of philtrum edge, projecting cleanly upward
			Long distance from columella		
	Cupid Arrow	Normal	Length variation	*N/A*	Line from base of columella to Cupid Peak, normal inclination
		Flat			
		Closed			
	Cupid Drop	Curved upward	*N/A*	*N/A*	Smooth vertical descent, M-shaped or gently curved downward
		Straight			
		Curved downward			
		M-shaped			
		Key-holed			
Lateral Zone	Climb Border	Straight	Slope asymmetry	Height deviation (uneven along curve)	Upward convergence toward Cupid’s system
		Curved			
		Mixed			
	Hill Border	Straight	Upward directionality	Height deviation (left–right difference)	Upward converging slope, symmetrical to contralateral side
		Curved			
			Horizontal directionality		
		Mixed			
Basal Zone	Base Border	Straight	Commissural position (before or at the commissure)	*N/A*	Straight or subtly convex
	Oval Border	Curved	Introflexed orientation	*N/A*	Smoothly curved outward (convex), continuous with basal transitions
			Extroflexed orientation		
*Lower lip*					
Medial Zone	Arc Border	Straight	*N/A*	*N/A*	Elliptical and curved downward for natural volume projection
		Curved			
	Fender Border	Straight	*N/A*	*N/A*	Oval-shaped, downward flowing
		Boat-like			
		Oval			
Drop Zone	Top Border	Curved	*N/A*	*N/A*	Elliptical curved with upward orientation
		Straight			
	Drop Border	Straight	Length variation	*N/A*	Elliptical, downward convex curve
		Curved			
		Mixed			
Sign Zone	Sign Border	Upward	*N/A*	*N/A*	Subtle convergence toward commissure, natural lateral flow
		Downward			
		Horizontal			

Symmetry can be assessed using standardized frontal photographs obtained under controlled conditions—bilateral landmarks are compared relative to the facial midline, and asymmetry expressed as proportional deviation, calculated as the relative difference between homologous left–right distances normalized to facial midline reference. *LV LEADS* Lip Visual Linear Elements for Aesthetic Design and Symmetry, *N/A* not applicable

The ‘Ideal status’ module can then be used to define the target configuration of the lips relative to their current status and in relation to surrounding features, delineated within the ‘Individual analysis.’ The aim is to define the goals of the shape-changing plan, i.e., the optimal geometric condition for each of the LV LEADS in relation to individual proportions. In this context, ‘ideal’ does not imply a universal or prescriptive aesthetic standard. Rather, it denotes a patient-specific target configuration that represents a structurally balanced and functionally coherent lip relationship, defined through clinical judgment and shared decision-making. Although these targets should always be individualized, they may be informed by established anthropometric norms and aesthetic principles. These include patient desires (structured during the ‘Mind consultation’), lip shape (the potential curvature and contour of each LV LEAD), proportional relationships, and functional considerations to ensure that enhancements support natural lip function and expression. Characteristic reference configurations for each LV LEAD are summarized in Table 4.

### PLAN: Strategic Injection Protocol

The PLAN phase translates the insights gathered through mapping and assessment into a precise HA filler injection strategy. It is based on four integrated components:

- Shape conversion
- Vectorial anatomy
- Placing pattern
- Products

Each of the four components functions as a procedural layer of intervention, allowing the clinician to execute aesthetic modifications using a logic-based framework, with high levels of personalization control.

For this technical phase, either needles or cannulas may be employed, depending on the clinician’s preference and treatment objectives, except where explicitly stated below. When using a needle, a 30G device (or at most 29G) is recommended to minimize tissue trauma and reduce the risk of post-procedural edema [15]. With a cannula, a 25G instrument is generally preferred for improved safety and lower risk of intravascular injection [16, 17].

The four components of the PLAN phase are described in order here. First, the ‘Shape conversion’ component translates the patient’s current lip morphology into a targeted visual transformation using precise geometric vectors and needle inclination angles. Conversion is based on the ‘Type’ of each LV LEAD; the symmetry and morphometric keys (‘KEYs’ and ‘KEYh’); and the inclination and projection angles applied to each segment. The goal is to change each of the LV LEADS according to their ideal and individual shape.

The foundational principle of the ‘Shape conversion’ component is the use of a needle inserted vertically, directly at the vermilion border. This allows greater control in shaping the lip architecture and may contribute to improved procedural control and safety**.** It also provides a stable reference plane and precise vectorial access for filler deposition. Variations of vertical injection techniques have been published previously, supporting this rationale with regard to both anatomical logic and clinical effectiveness [18].

**Fig. 4:**
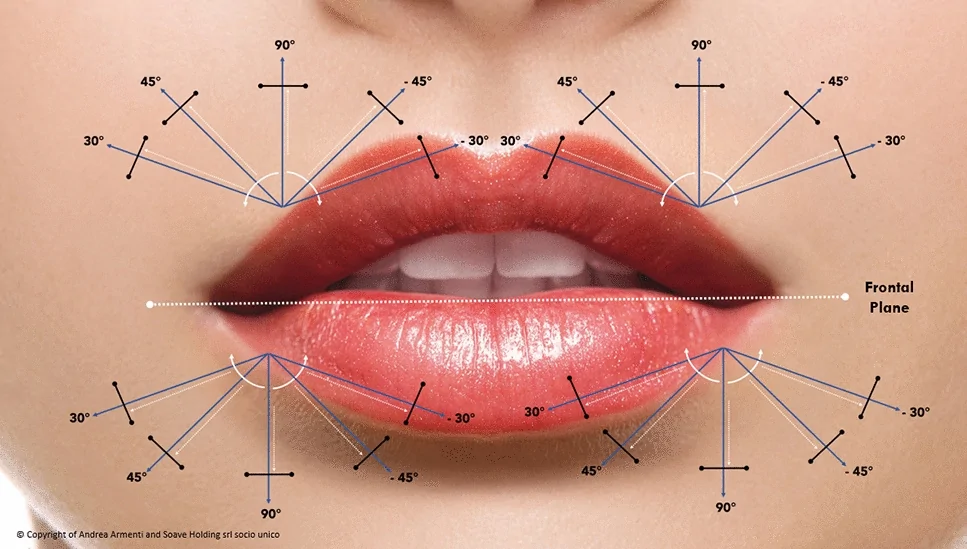
Frontal planes of inclination

The inclination angle of the needle—in the frontal and sagittal planes—is crucial to executing these changes. In the frontal plane, three needle inclinations guide the impact of changing the shape of individual sections of LV LEADS (based on an entry point at the vermilion border). These are shown in Fig. 4. Needle inclination at 90° provides vertical elevation of that section of the LV LEAD; inclination at 45° facilitates upward movement without modifying shape (parallel movement); and inclination at 30° changes the LV LEAD section by moving it laterally. These angles can be applied in either a positive or a negative direction relative to the frontal plane (e.g., + 30° or – 30°), depending on whether the clinician needs to direct the needle toward the patient’s right or left side. This bidirectional angular control allows the clinician to adapt the shape modification vector laterally, adjusting each of the LV LEADS according to its spatial orientation and asymmetry.

Similarly, in lateral view, three inclination angles guide how the needle (and thus the HA filler) goes superficially or deep inside the lip tissue from a vermilion border entry point. An inclination of 0° creates a neutral vertical movement with the needle remaining parallel to the vermilion plane and the filler gel going into the subareolar tissue (SAT); needle inclination at 30° creates a subtle anterior vertical movement, with the gel going deeper into the SAT; and needle inclination at 45° creates middle anterior vertical movement, with the needle going under the SAT and the HA filler going to the marginal part of the orbicularis oris muscle.

It should be emphasized that the ‘Shape conversion’ component is intrinsically dependent on needle use. Only a needle allows the angular precision and vectorial control required to modify the shape of individual LV LEADS. These corrections—based on defined inclination angles—cannot be effectively performed with a cannula. However, the other PLAN components below remain fully applicable regardless of the chosen injection device. If the clinician prefers to use a cannula, the PLAN protocol can still be implemented, but without the shape-modifying potential of the ‘Shape conversion’ layer.

**Fig. 5:**
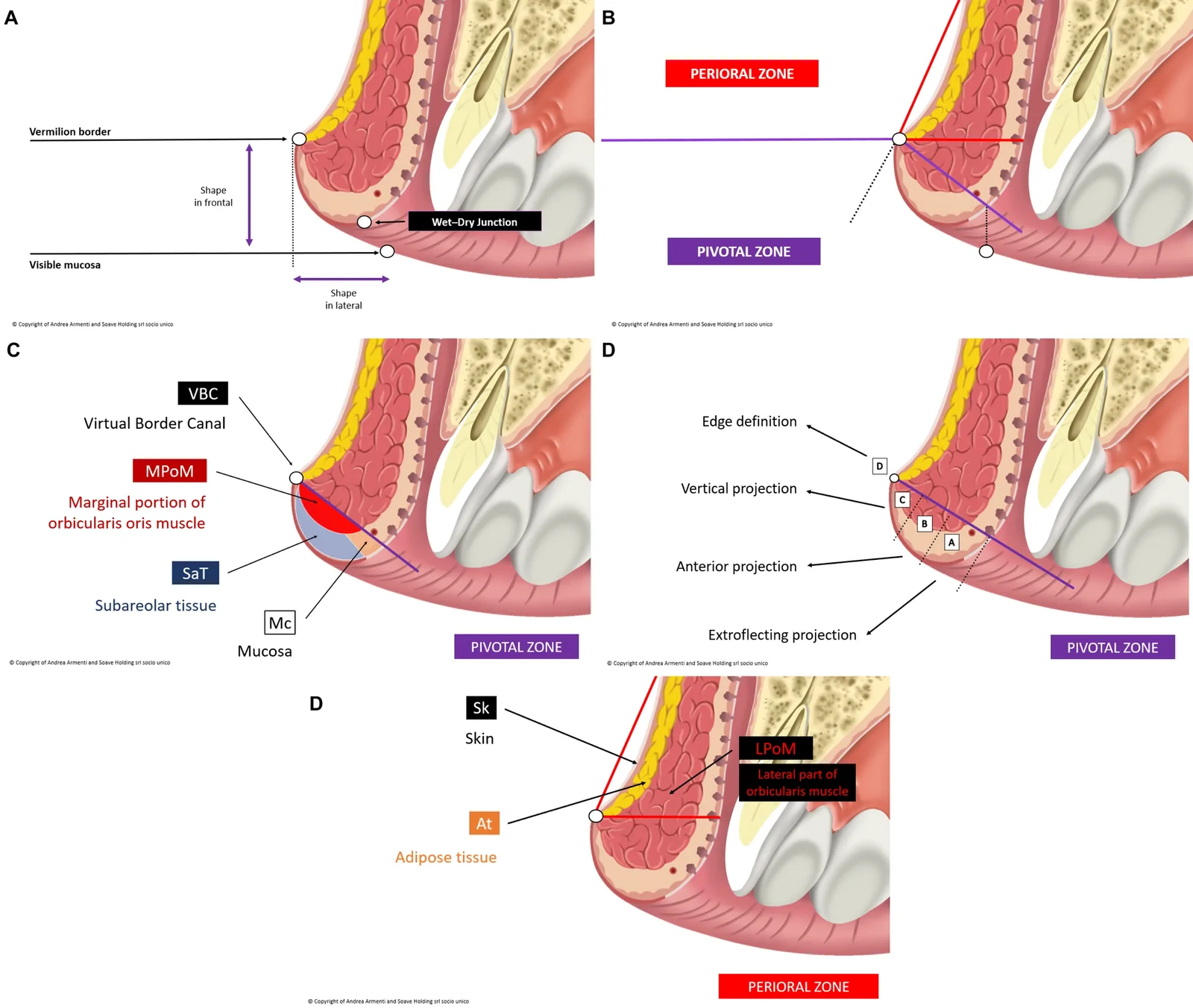
Pivotal and Perioral Zones. Parts **A** and **B** show the delineation of the Pivotal and Perioral Zones. Part **C** shows the layered anatomy of the Pivotal Zone. Part **D** demonstrates the four-section framework of the Pivotal Zone. In the latter, section A (lower part of the lip) allows outward tissue expansion by lowering the inferior margin and contributing to extroflection. Section B (central part) allows anterior tissue expansion by projecting the central part of the lip and contributing to anterior projection. Section C (higher part) facilitates vertical enhancement of tissue. Section D (top edge) allows sculpting of the lip border. Part **E** shows the layered anatomy of the Perioral Zone

Within the ‘Vectorial anatomy’ component, lip anatomy is organized into two zones, each with implications for injection planning: the Pivotal Zone and the Perioral Zone (Fig. 5A and B). Regarding the Pivotal Zone, key regions are the wet–dry junction (defining the threshold between the vermilion and mucosa layer) and the lip border (defining the threshold between vermilion and perioral skin). In a frontal plane, viewing down from a dividing line passing through the vermilion border, there are four layers (from anterior to posterior). These are the subareolar layer (tissue between vermilion and orbicularis oris muscle) [SAT]; muscular layer (marginal portion of orbicularis oris muscle) [MPoM]; mucosa layer [Mu]; and virtual border canal (structure of tissue conjunction below the lip border) [VBC] (Fig. 5C). This is called the ‘Pivotal Zone’ because only HA filler placed here allows for changes in lip shape.

It should be noted that the term ‘subareolar layer’ is used here for describing the tissue located between the dry vermilion epithelium and the orbicularis oris muscle, in order to distinguish it from the ‘submucosal layer’ that underlies the wet mucosa beyond the wet–dry line. The latter term is frequently used in the literature to describe both regions despite their different anatomical and functional characteristics.

Seen in a frontal plane caudo-cranially, the Pivotal Zone can be subdivided into four strategic sections, each associated with a dominant functional transformation (Fig. 5D). Specifically, section A (the lower part of the lip) allows outward tissue expansion by lowering the inferior margin and contributing to extroflection; section B (the central part) allows anterior tissue expansion by projecting the central part of the lip and contributing to anterior projection; section C (the higher part) facilitates vertical enhancement of tissue; and finally section D (top edge) allows sculpting of the lip border. Each section is associated with corresponding tissue layers and injection depths.

With regard to the Perioral Zone, viewed in a frontal plane (up from a dividing line passing through the vermilion border) and running from anterior to posterior, there are the following layers: skin; subcutaneous/connective layer; muscular layer (pars lateralis of the orbicularis oris muscle); and the mucosa layer. Treatment here does not alter lip shape but rather can be used to manage the perioral area (Fig. 5E).

A further key consideration relates to safety, particularly with regard to avoiding vascular compromise. Detailed anatomical understanding of the typical positions of the superior and inferior labial arteries is essential [19, 20]. Placement within the SAT is commonly considered a lower-risk plane for shape-oriented deposits compared with deeper planes; nevertheless, vascular safety cannot be guaranteed by plane selection alone and remains dependent on integrated anatomical knowledge and cautious technique. In selected clinical situations—most notably when wanting anterior projection of the upper lip or myomodulatory effects on the orbicularis oris muscle—the filler material may need to be placed within the MPoM. This layer inherently carries a higher theoretical risk of vascular compromise. However, both safety and effectiveness can be supported through a combination of anatomical knowledge, cautious technique, and precise control of injection parameters. When available, ultrasound guidance may further enhance spatial awareness and risk assessment.

Once the ‘Shape conversion’ and ‘Vectorial anatomy’ have been mapped, the third component—‘Placing pattern’—can be used to define how to distribute product volumetrically and geometrically across the lip architecture. This component turns design logic into a precise pattern of injections, balancing aesthetic intent with anatomical safety. It integrates the four sections of the Pivotal Zone (A–B–C–D) and relevant anatomical layers (SAT, MPoM, VBC, Mu) with a dose strategy (volume range per plane) and dose patterns.

With regard to dose strategy, the injection amounts—known as ‘Vector doses’—should be precisely tailored according to the expansion and definition needed (Table 5), to match functional transformation with anatomical feasibility. In this context, the reported micro-volumes reflect the resolution at which clinical decisions are articulated within the method rather than fixed injection units. The entry point is along the lip border, with a stop point at the wet–dry junction, which is a natural anatomical boundary. The needle angle should be as defined in the ‘Shape conversion’ component. With respect to the dosing pattern, HA filler gels support specific vector forces, and they shape the pattern of distribution (Table 6). These shapes are functional designs and not mere aesthetic preferences. Each shape promotes either movement (dynamic injection) or expansion (static deposit) and must be matched to the tissue plane and desired effect.

**Table 5:** Pivotal Zone sections and vector dose strategy

Section	Transformation Type	Clinical Focus	Layer	Vector Dose (Range Per Section/Fence)
A	Extroflection	Outward expansion at vermilion border	SAT	0.01–0.04 mL
B	Anterior projection	Forward volume for contour	SAT/MPoM	0.01–0.04 mL
C	Verticalization	Vertical enhancement and lift	SAT/MPoM	0.01–0.03 mL
D	Edge definition	Sculpted edge border	VBC	Maximum 0.01 mL

*MPoM* marginal portion of orbicularis oris muscle, *SAT* subareolar tissue, *VBC* virtual border canal

**Table 6:** Dose patterns

HA shape (single section/fence)	Vector type	Use case
Bolus	Expansion vector	Non-moving injection for volumetric push

HA shape (pan sections [A–B–C–D]/fence)	Vector type	Use case
Line	Linear force vector	Retrograde injection for lip margin elevation
Lollipop	Static expansion vector	Vertical projection and definition at borders
Inverted cone	Converging pressure	Point-specific projection

*HA* hyaluronic acid

**Fig. 6:**
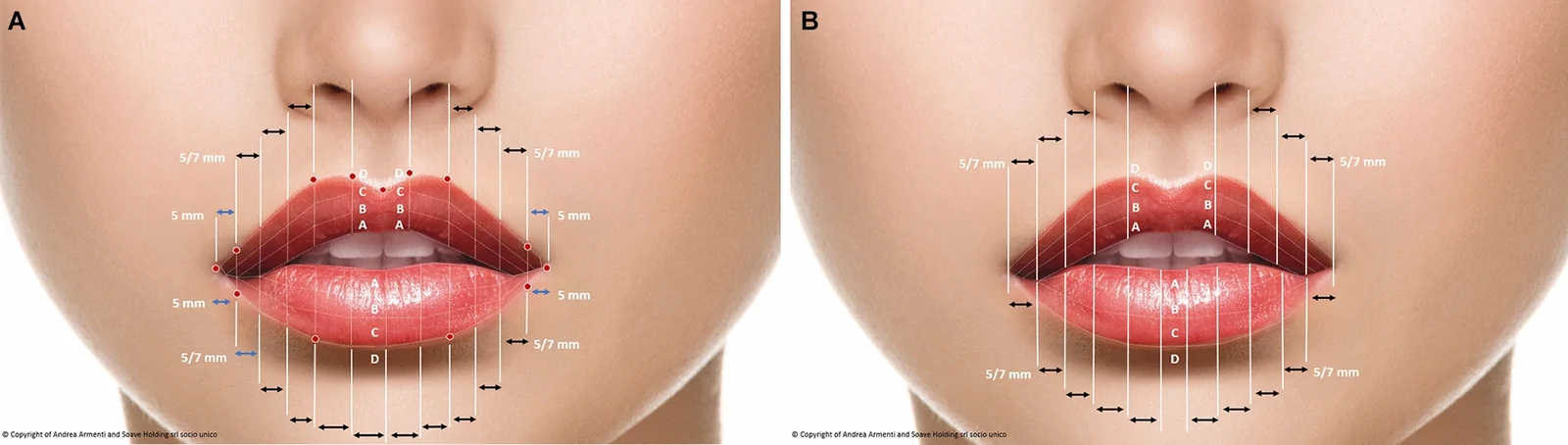
Distribution logic. Distribution logic with a needle (part **A**) or cannula (part **B**)

When using a needle, this distribution logic follows the vertical vector-based design defined in the ‘Shape conversion’ component, in which injections are aligned with vertical ‘fences’ and the A–B–C–D sectioning of the Pivotal Zone, with a 5-mm spacing per entry point (Fig. 6A). However, when using a cannula, the geometry of product placement shifts from vertical to horizontal logic. While the same sectioning of the lip (A–B–C–D) remains valid, an additional frontal grid system should be applied to guide horizontal filler distribution. This grid consists of evenly spaced vertical lines at 5-mm intervals, drawn across the lip in the frontal plane. These vertical gridlines act as a horizontal reinterpretation of the fences used in needle-based injection, helping the clinician to achieve symmetrical, layered deposition of the product with respect to both anatomy and aesthetic design (Fig. 6B).

The fourth and final component of the PLAN phase relates to ‘Products.’ HA filler selection should be based on rheological properties and prediction of tissue response. It is essential to match filler behavior with the geometric intent defined within the ‘Shape conversion’ component (if a needle is to be used), the anatomical realities of ‘Vectorial anatomy,’ and the injection logic set by the ‘Placing pattern.’ In essence, it transforms the HA filler into a dynamic tool for sculptural modulation.

Key rheological parameters that define the appropriate usage of different HA filler gels include [21]:

- Elastic modulus (*G*’): The resistance to deformation, with a higher *G*’ giving greater structure.
- Complex modulus (G*): The global resistance in three-dimensional movement.
- Viscoelastic ratio (tan *δ*): The balance between viscosity and elasticity, with a high tan *δ* defining greater blending ability.

A matching guide is provided in Table 7. Clinicians must appreciate that if they are using a ‘denser’ HA gel, they will require smaller product quantities per injection site, a less complicated shape (for example linear and not bolus), and a deeper injection plane (considering needle inclination and tissue layer). On the flipside, when using a ‘softer’ HA gel, they will need a greater quantity of product, a more sophisticated shape (e.g., inverted cone or bolus), and a more superficial injection plane.

**Table 7:** Product matching guide

REQUEST →		Hydration			Restore		Volume		Divergent smile	Asymmetry
AGE (years) →		18–30		> 30	18–30	> 30	18–30	> 30	> 18	> 18
Pivotal Zone	SAT + VBC	Juvéderm Volbella Hydromax RHA I Restylane Vital* Flux			Juvéderm Volbella Stylage S/Special Lips RHA II/KISS Aliaxin FL Restylane Kysse	Juvéderm 3/Volift Stylage S/Special Lips RHA II/KISS Intense Rheology Aliaxin FL Restylane Kysse	Juvéderm 3 Stylage Lips Plus RHA III Intense Lips Aliaxin LV		Go to ‘Restore’ + ‘Volume’	Go to ‘Restore’ + ‘Volume’
	MPoM	X	X		Juvéderm Volbella Stylage Special Lips RHA KISS Restylane Kysse	Juvéderm 2/Volift Stylage Special Lips RHA II/KISS Intense Rheology Restylane Kysse	Juvéderm 3 Stylage Special Lips RHA III Aliaxin LV			
Perioral Zone^a^	SK	Juvéderm Volite Hydro Hydro Deluxe RHA I Profhilo Restylane Vital Light			X	Juvéderm Volite Hydro RHA I Hydro Deluxe Profhilo Restylane Vital	X	X	Go to ‘Hydration’ + ‘Restore’	
	At	X	X		X	Juvéderm Volbella Hydromax RHA II Flux Aliaxin FL Restylane Refyne	X	Juvéderm Volift Stylage S/M RHA II/III Flux/Intense Rheology Aliaxin GP Restylane Refyne	Go to ‘Restore’ + ‘Volume’	

This matching guide covers relevant fillers from the AbbVie/Allergan, Vivacy, Teoxane, Neauvia, IBSA and Galderma ranges, but other products with equivalent rheological properties are available. ^a^The ‘perioral zone’ is the operational label corresponding 1:1 to the ‘perioral’ category (Mind consultation). *At* adipose tissue, *MPoM* marginal portion of orbicularis oris muscle, *SAT* subareolar tissue, *SK* skin, *VBC* virtual border canal

### Safety Considerations

The LA VISION Method is firmly anchored in key principles of anatomical respect and patient safety. The lip region is rich in vasculature and is sensitive from a neurofunctional perspective. All treatments must therefore be performed within strict safety protocols.

The method does not aim to directly identify or predict the exact course of the labial arteries. However, it does incorporate indirect risk-modulating elements—such as injection plane selection, depth control, conservative volumetric thresholds, and decision sequencing—designed to reduce vascular risk without relying on surface landmark-based artery localization. The spatial ranges used within the MAP phase are not intended to define injection points, but to support zoning and decision sequencing, informed by anatomical considerations and known vascular depth distributions. Their magnitude has been informed by ultrasound-based anatomical data, ensuring coherence with known vascular depth and plane distributions, thereby integrating topographic planning with vascular risk modulation.

Moreover, spatial references from the oral commissure are used as contextual ranges rather than fixed measurements. These references are anchored to published ultrasound data describing the origin, plane, and depth of the superior and inferior labial arteries, which are typically located at ~4 to 6 mm in depth and most frequently within the submucosal or intramuscular planes of the red lip [19, 20]. Therefore, such spatial references are intended to support proportional orientation and risk-modulating clinical decisions rather than to define universally applicable ‘safe points.’

The vector planning considers not only aesthetic outcome but also tissue tension and muscle dynamics, thereby avoiding overcorrection or distortion in motion. Moreover, product delivery is based on micro-volumes, reducing the risk of compression, ischemia, or migration. The method promotes a minimalistic mindset, favoring structure and balance over volume—important for reducing adverse events.

Nonetheless, clinicians must always remain particularly vigilant for signs of vascular compromise and adhere to standard emergency protocols, including the availability of hyaluronidase [22, 23]. The use of aspiration and slow injection techniques are encouraged as routine best practices.

### Patient Cohort

A retrospective analysis was conducted based on 400 consecutive adults treated with the LA VISION Method between December 2023 and July 2025. All patients were treated at baseline (T0) and scheduled for maintenance at ~6 months (T6M) and ~1 year (T1Y). However, not all patients received T1Y, and thus the total number of treatment sessions undertaken was 1039 (T0, *n*=400; T6M, *n*=400; T1Y, *n*=239). At each maintenance session, the injection device and filler used at T0 were maintained whenever clinically appropriate.

Patients were excluded if they had previous lip filler treatment, active infection at the treatment site, or pregnancy/breastfeeding. To balance clinical realism with analytical rigor, a controlled indication taxonomy was used. When multiple desires were expressed, a single primary indication was assigned based on the objective that most directly determined zone selection and dose allocation at T0. Any additional categories were recorded as co-indications (non-mutually exclusive modifiers) from the same list (Volume, Restoration, Hydration, Asymmetry, Smile [i.e., divergent smile]). The ‘Perioral’ category was intentionally excluded as its LV LEADS belong to a separate framework not covered here and will be reported in a dedicated paper. Thus, analyses focus on lip shape categories only.

Adverse events (AEs) were recorded throughout 12 months of follow-up. Primary-based analyses were chosen a priori to prevent double counting of volumes and AEs across categories and to preserve statistical independence between groups. Late-onset swelling was defined a priori as swelling/edema explicitly described as delayed/late/tardive at the relevant timepoint. AEs were abstracted verbatim from the complications fields at T0, T6M, and T1Y using rule-based keyword matching (prespecified patterns; mild severity unless otherwise stated). AE rates are computed per treatment session at each timepoint (T0, *n*=400; T6M, *n*=400; T1Y, *n*=239; overall, *n*=1039).

Domain-level patient-reported outcome measures (PROMs) were based on 10-point numeric rating scales (0 = worst/least satisfied, 10 = best/most satisfied). The domains used were lip appearance satisfaction, psychological well-being, and social confidence, each assessed at T0, T6M, and T1Y. In addition, treatment satisfaction was assessed at T6M and T1Y. The denominators reflect survey completion (*n*=400 at T0, T6M, and T1Y). Changes from baseline were calculated as mean differences with 95% confidence intervals (CIs) from paired-*t* analyses using only participants with data at both timepoints. Effect sizes were reported as Cohen’s d for paired samples (dz = mean of paired differences divided by the standard deviation of paired differences). For completeness, the standardized mean change using the baseline standard deviation (sometimes denoted dh = [\bar{x}T − \bar{x}0]/SDT0) is an alternative; we use dz throughout. PROMs were administered in clinic using standardized instructions. These are not validated instruments and should be considered as descriptive and supportive rather than confirmatory; no formal psychometric validation was attempted.

In the finalized analysis dataset, baseline (T0) treatment volumes were standardized to ≤ 1.00 mL per patient after quality control to harmonize reporting, while preserving proportional zone composition and all safety summaries. Because the study was not designed or powered for hypothesis testing, no inferential analyses were planned. Continuous outcomes are reported as mean ± standard deviation. Proportions are expressed as percentages with Wilson 95% CIs.

At the product level, AE proportions were summarized with Wilson 95% CIs; for comparative context, observed/expected (O/E) outcomes are reported, defined as the product-specific rate divided by the cohort-wide rate, and a composite ‘swelling’ endpoint (= edema + late-onset swelling). Eligibility required complete baseline records and ≥ 1 follow-up (T6M or T1Y).

In addition, a sensitivity analysis was carried out to assess AE rates and treatment satisfaction in patients with a single indication compared with mixed indications for treatment.

Procedures were performed in accordance with the 1964 Helsinki declaration and its later amendments, and all patients gave written informed consent prior to treatment. In addition, three separate case presentations are provided below to demonstrate the utility of the method. These individuals gave written informed consent for the use of their images.

## Results

A total of 400 patients who underwent HA-based lip augmentation according to the LA VISION Method were included in the present analysis (Table 8). Patients were scheduled for three sessions (T0, T6M, T1Y), and 1039 treatments were undertaken overall (T0, *n*=400; T6M, *n*=400; T1Y, *n*=239). The mean age of the cohort was 41.0 ± 13.9 years (range: 18–64), and 213 were female (53.2%). They had a mean of 11.2 ± 0.4 non-straight LV LEADS (i.e., classified as curved or mixed at baseline rather than straight).

**Table 8:** Baseline characteristics and treatment overview

Characteristic	Patients (*N*=400)
Age, years, mean ± SD (range)	41.0 ± 13.9 (18–64)
*Sex, n (%)*	
Female	213 (53.2)
Male	187 (46.8)
Non-straight LV LEADS, mean ± SD	11.2 ± 0.4
*Primary indication, n (%)*	
Volume enhancement	19 (4.8)
Restoration	166 (41.5)
Hydration	38 (9.5)
Smile correction	33 (8.3)
Asymmetry management	144 (36.0)
*Injection device, n (%)*	
Needle	344 (86.0)
Cannula	56 (14.0)
*HA volume injected, mL, mean ± SD*	
T0 (*n*=400)	1.00 ± 0.01
T6M (*n*=400)	0.80 ± 0.06
T1Y (*n*=239)	0.60 ± 0.06

*HA* hyaluronic acid, *LV LEADS* Lip Visual Linear Elements for Aesthetic Design and Symmetry, *SD* standard deviation, *T0* baseline treatment, *T1Y* 1-year treatment, *T6M* 6-month treatment

The most common primary indications were based on restoration (*n*=166; 41.5%) and asymmetry management (*n*=144; 36.0%). Primary indication counts were mutually exclusive (sum to 100%) and reflect the main driver of planning at T0. Co-indications were coded separately as modifiers and are summarized in Supplementary Table 2. Approximately one third of patients presented with mixed indications; the most frequent pairs were ‘Volume’ + ‘Asymmetry’ and ‘Volume’ + ‘Restoration.’

In their first treatment session (T0), most were injected using a needle (*n*=344; 86.0%), and the mean volume of HA used was 1.00 ± 0.01 mL. Smaller HA volumes were required in the second session at T6M (0.80 ± 0.06 mL) and in the third session at T1Y (0.60 ± 0.06 mL), confirming shape stabilization.

Safety findings are listed in Table 9 for the 1039 treatment sessions. The most commonly reported AEs were mild localized (non-late) edema (*n*=136; 13.1%) or bruising (*n*=93; 9.0%), typically appearing within 2–3 days post-treatment. In most cases, these issues resolved spontaneously and did not require further intervention. When not contraindicated, a short standardized course of oral corticosteroids was used (prednisolone 40 mg/day for 5 days), and this may have contributed to reducing local reactions (although no formal causal inference can be drawn). Late-onset swelling occurred in 4 sessions (0.4%), generally linked to external factors, such as upper respiratory infection, fatigue, or sinusitis. Patients with a history of multiple allergies appeared to be at greater risk. One individual (0.1%) reported the formation of a single HA nodule in the mucosal tissue 1 month after treatment, which was dissolved using 5U of hyaluronidase. There were no obvious cases of filler migration, and no major complications, such as vascular occlusion or tissue necrosis.

**Table 9:** Adverse events per treatment session

Event	T0 [*N*=400]	T6M [*N*=400]	T1Y [*N*=239]	Overall [*N*=1039]
Edema (non-late)	69 (17.3) [13.9–21.3]	48 (12.0) [9.2–15.6]	19 (7.9) [5.2–12.1]	136 (13.1) [11.2–15.3]
Bruising	42 (10.5) [7.9–13.9]	38 (9.5) [7.0–12.8]	13 (5.4) [3.2–9.1]	93 (9.0) [7.4–10.8]
Late-onset swelling	2 (0.5) [0.1–1.8]	2 (0.5) [0.1–1.8]	0 (0) [0.0–1.6]	4 (0.4) [0.2–1.0]
Nodule formation	0 (0) [0.0–1.0]	1 (0.3) [0.0–1.4]	0 (0) [0.0–1.6]	1 (0.1) [0.0–0.5]
Filler migration	0	0	0	0
Infection	0	0	0	0
Vascular compromise	0	0	0	0
Tissue necrosis	0	0	0	0

All data are reported as n (%) [Wilson 95% CI]. Edema counts exclude episodes explicitly labeled as late/delayed/tardive, which are reported separately as late-onset swelling. *CI* confidence interval, *T0* baseline treatment, *T1Y* 1-year treatment, *T6M* 6-month treatment

Patient-reported outcomes are summarized in Table 10. Baseline mean scores were 5.80 ± 0.99 for lip appearance satisfaction, 6.43 ± 0.83 for psychological wellbeing, and 6.44 ± 0.86 for social confidence. At T6M, mean scores had increased to 8.20 ± 0.92, 8.02 ± 0.86, and 8.06 ± 0.89, respectively. Mean changes from baseline were +2.40 (95% CI: 2.36–2.44), +1.59 (95% CI: 1.55–1.62), and +1.62 (95% CI: 1.58–1.65), and corresponding effect sizes (Cohen’s d, paired; dz) were 5.85, 4.41, and 4.47. At T1Y, mean scores were 7.73 ± 0.96, 7.66 ± 0.84, and 7.68 ± 0.89, respectively. Mean changes from baseline were +1.93 (95% CI: 1.89–1.98), +1.23 (95% CI: 1.19–1.27), and +1.25 (95% CI: 1.21–1.28), with effect sizes of 4.39, 3.23, and 3.21, respectively, indicating sustained improvements. Mean treatment satisfaction was 8.05 ± 0.59 at T6M and 7.51 ± 0.63 at T1Y.

**Table 10:** Patient-reported outcome scores

Domain	T0 mean ± SD	T6M mean ± SD	T1Y mean ± SD	ΔT6M–T0 mean (95% CI) [effect size]	ΔT1Y–T0 mean (95% CI) [effect size]
Lip appearance satisfaction	5.80 ± 0.99	8.20 ± 0.92	7.73 ± 0.96	2.40 (2.36–2.44) [5.85]	1.93 (1.89–1.98) [4.39]
Psychological well being	6.43 ± 0.83	8.02 ± 0.86	7.66 ± 0.84	1.59 (1.55–1.62) [4.41]	1.23 (1.19–1.27) [3.23]
Social confidence	6.44 ± 0.86	8.06 ± 0.89	7.68 ± 0.89	1.62 (1.58–1.65) [4.47]	1.25 (1.21–1.28) [3.21]
Treatment satisfaction	–	8.05 ± 0.59	7.51 ± 0.63	–	–

*N*=400 at each timepoint. Denominators reflect survey completion and are independent of treatment occurrence at each timepoint. Changes from baseline (Δ) use complete pairs with t-based 95% CIs and effect sizes reported as paired samples Cohen’s d (dz). *CI* confidence interval, *SD* standard deviation, *T0* baseline treatment, *T1Y* 1-year treatment, *T6M* 6-month treatment

Stratified analyses across primary indication, product, device (needle vs cannula), and timepoint (T0/T6M/T1Y) showed broadly comparable AE rates and consistent PROM gains, supporting the broad applicability of the LA VISION Method (Supplementary Tables 3, 4, 5, 6; Tables 9, 10). AE rates decreased over time (e.g., edema: T0, 17.3%; T6M, 12.0%; T1Y, 7.9%; bruising: T0, 10.5%; T6M, 9.5%; T1Y, 5.4%) (Table 9), while rates of late-onset swelling remained ≤ 0.5% (0% at T1Y). Treatment satisfaction was consistent across analyzed subgroups. Product-level differences were modest. For example, rates of edema were 0% with Juvéderm Ultra 3, Intense Rheology, and Flux; 2.9% with RHA III; and 10.4% with Stylage Lips Plus. No primary category or device exhibited a systematic excess risk. Altogether, outcomes appeared to be driven primarily by LV LEADS-based planning and technique, rather than by brand choice or delivery device.

**Fig. 7:**
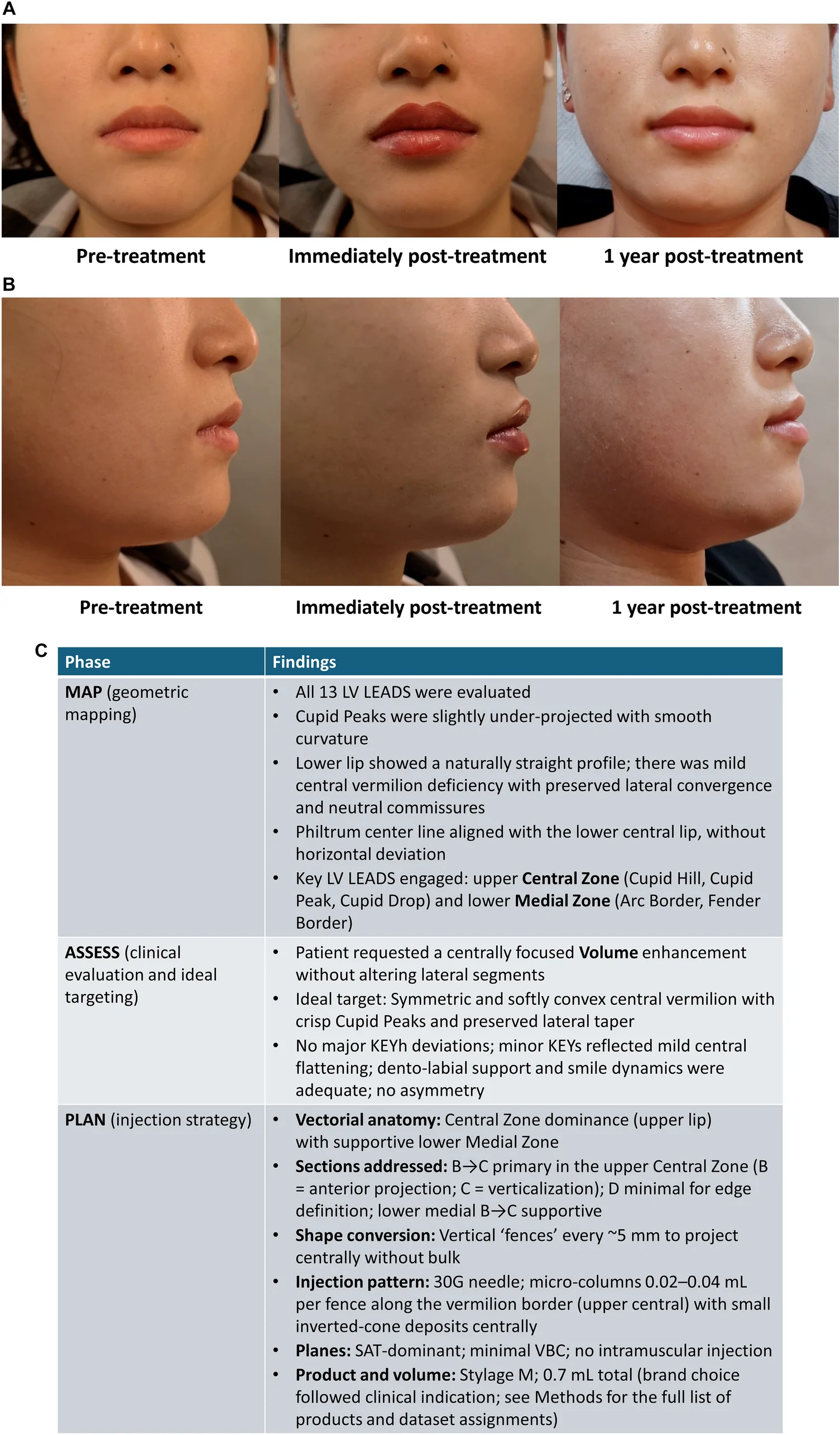

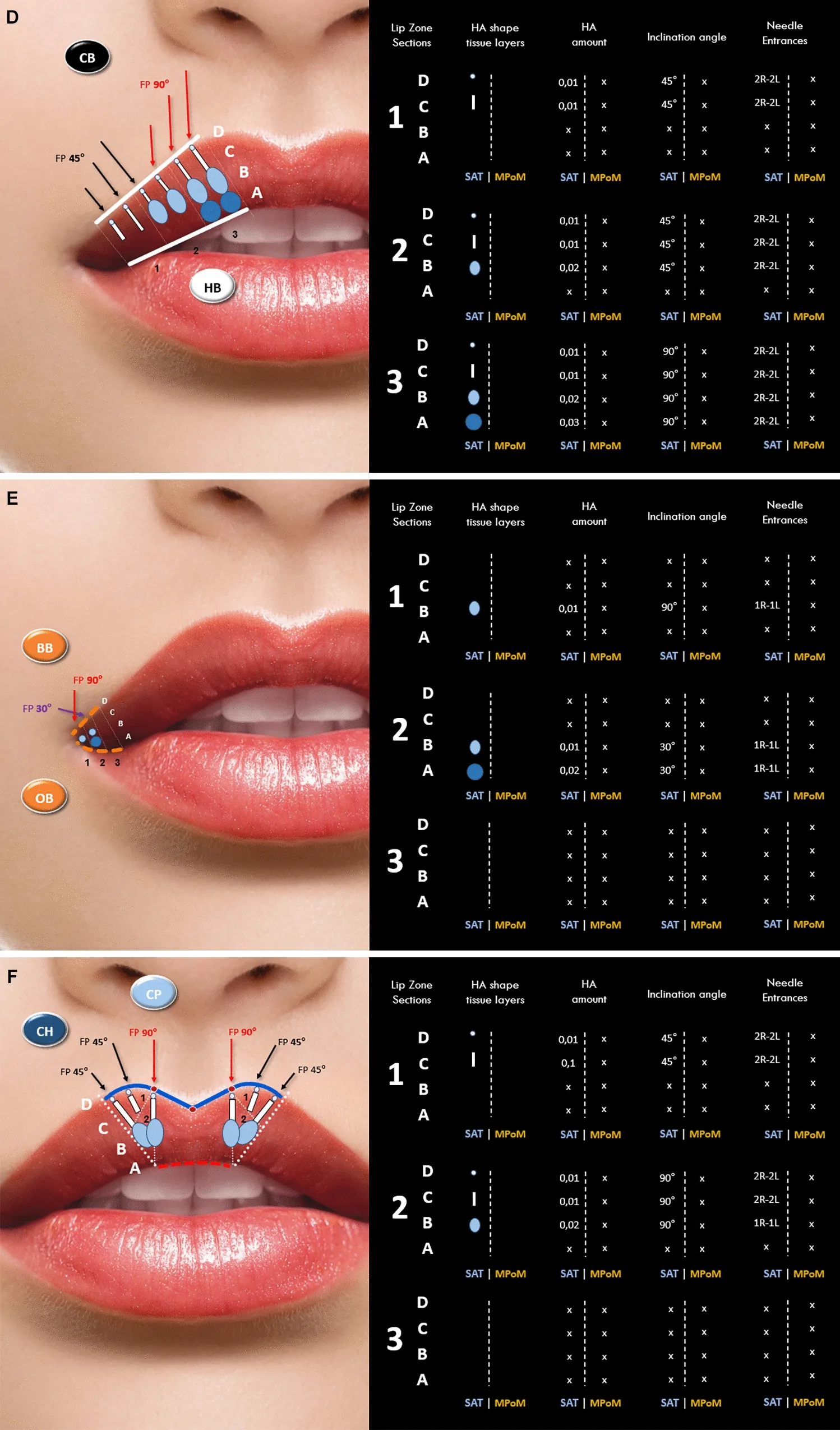

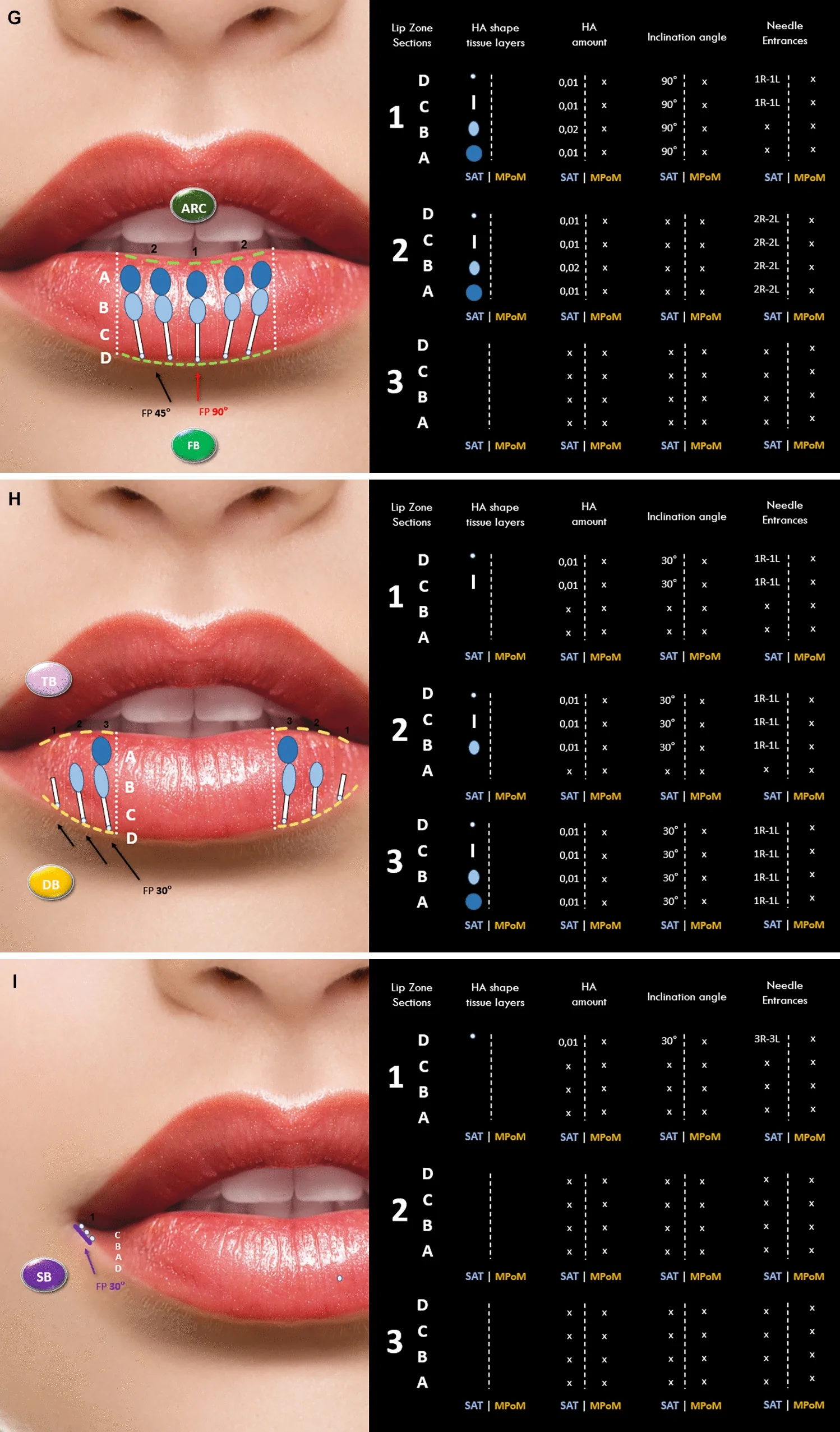
A 25-year-old female treated using the LA VISION Method: Central definition with balanced projection. Parts **A** and **B** show the patient before and after treatment. Part **C** summarizes the workflow performed using the LA VISION Method. Parts **D**–**I** show the strategic injection protocol. *ARC* Arc Border, *BB* Base Border, *CB* Climb Border, *CH* Cupid Hill, *CP* Cupid Peak, *DB* Drop Border, *FB* Fender Border, *FP* frontal plane, *HA* hyaluronic acid, *LV LEADS* Lip Visual Linear Elements for Aesthetic Design and Symmetry, *L* left, *MPoM* marginal portion of orbicularis oris muscle, *OB* Oval Border, *R* right, *SAT* subareolar tissue, *SB* Sign Border, *TB* Top Border, *VBC* virtual border canal

**Fig. 8:**
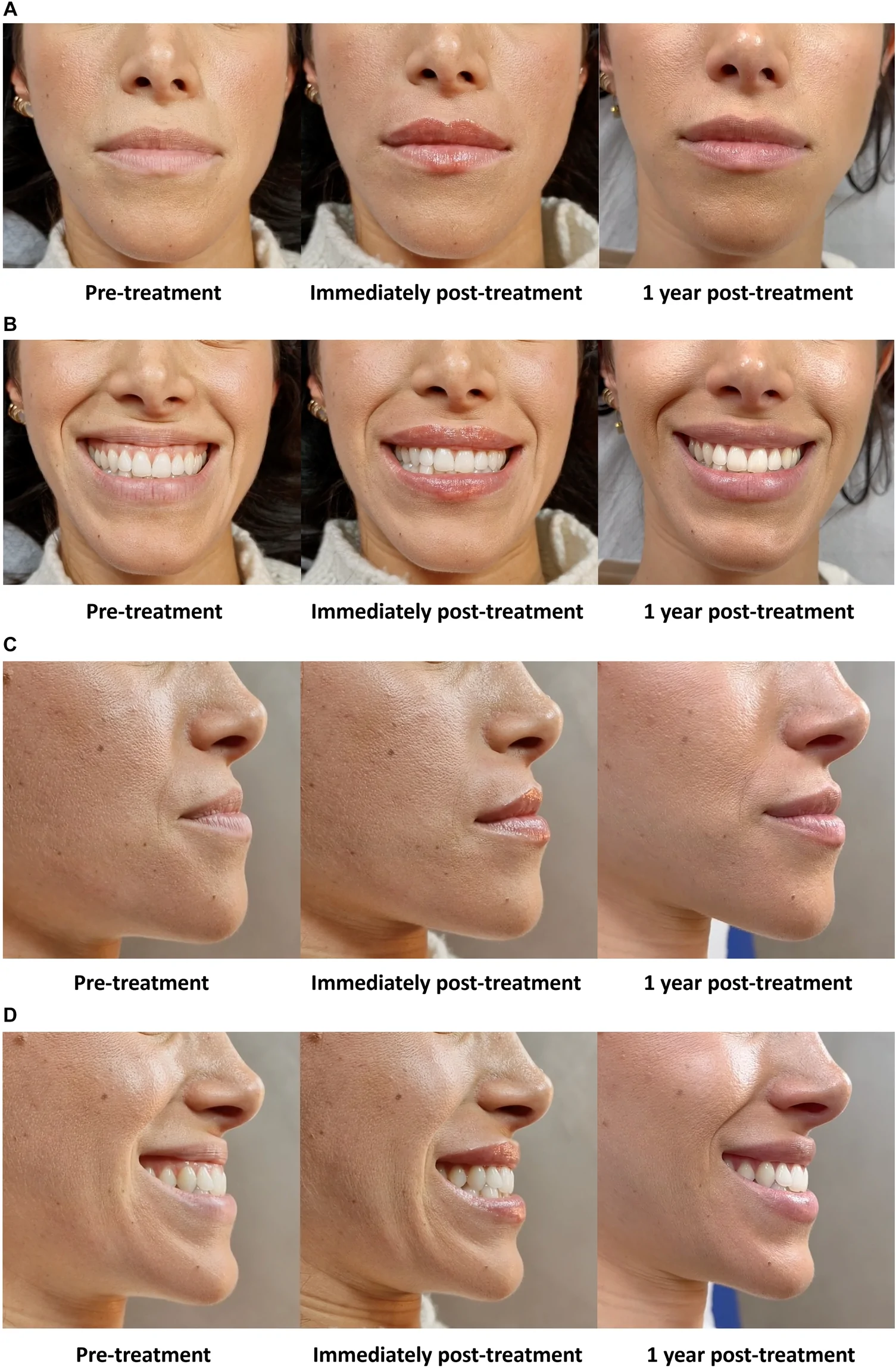

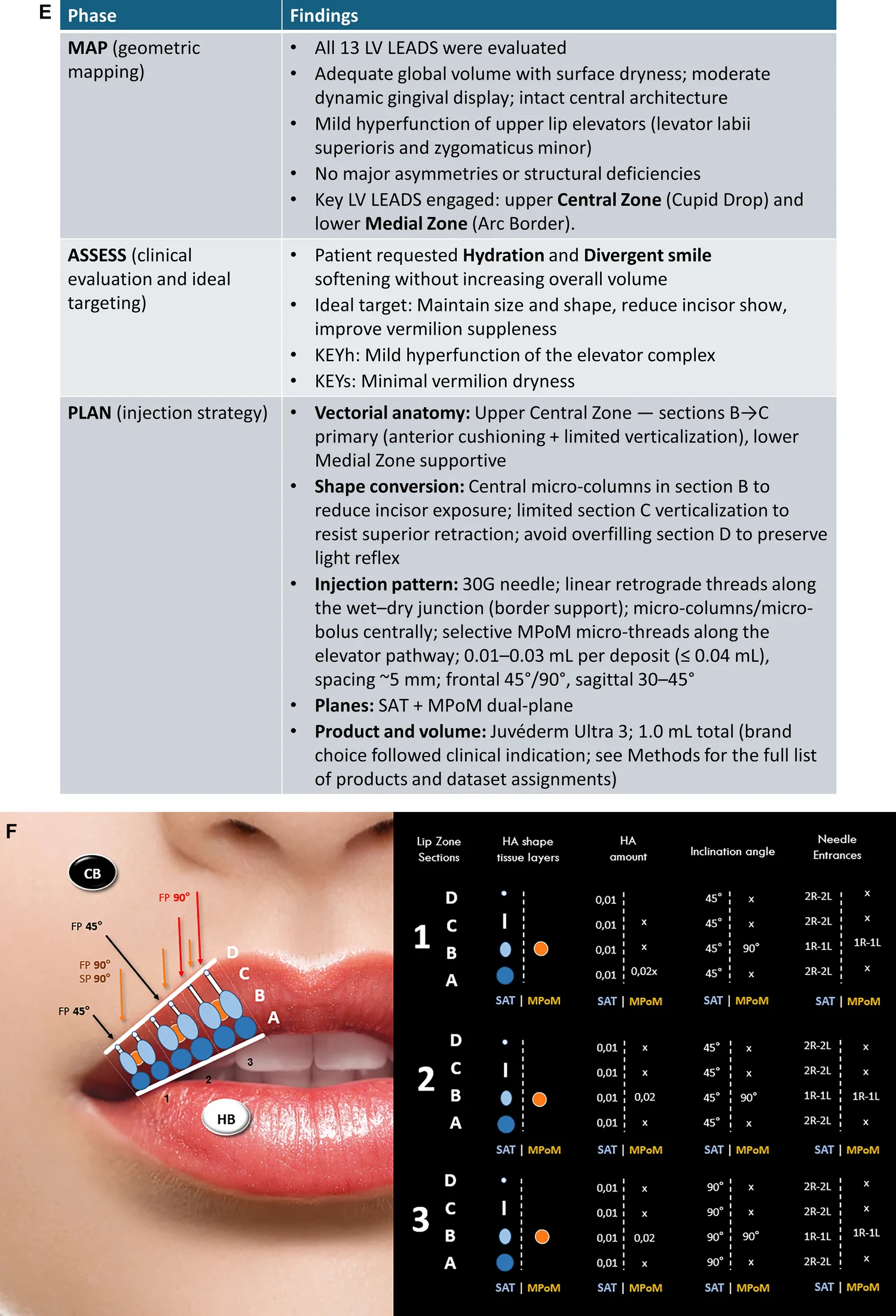

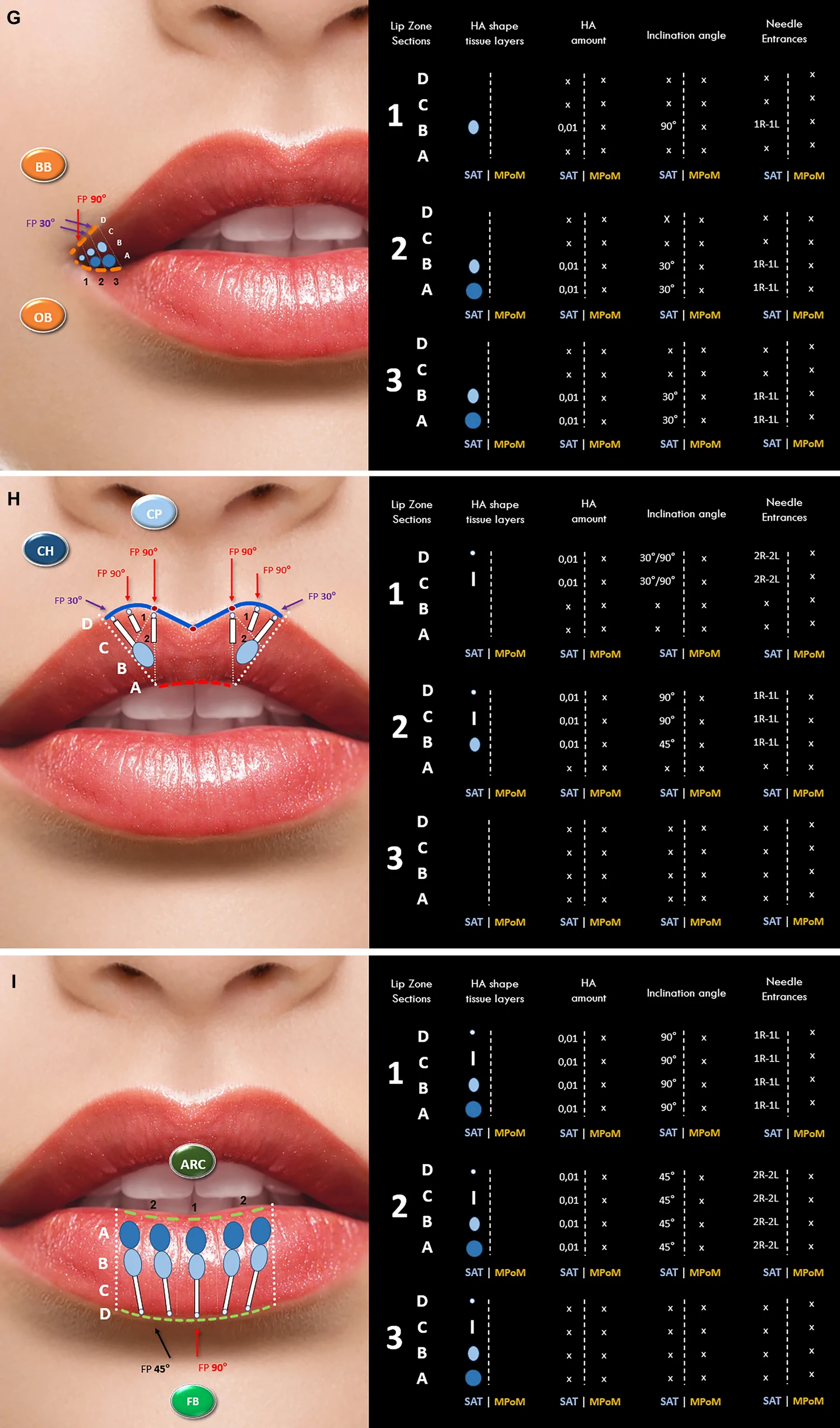

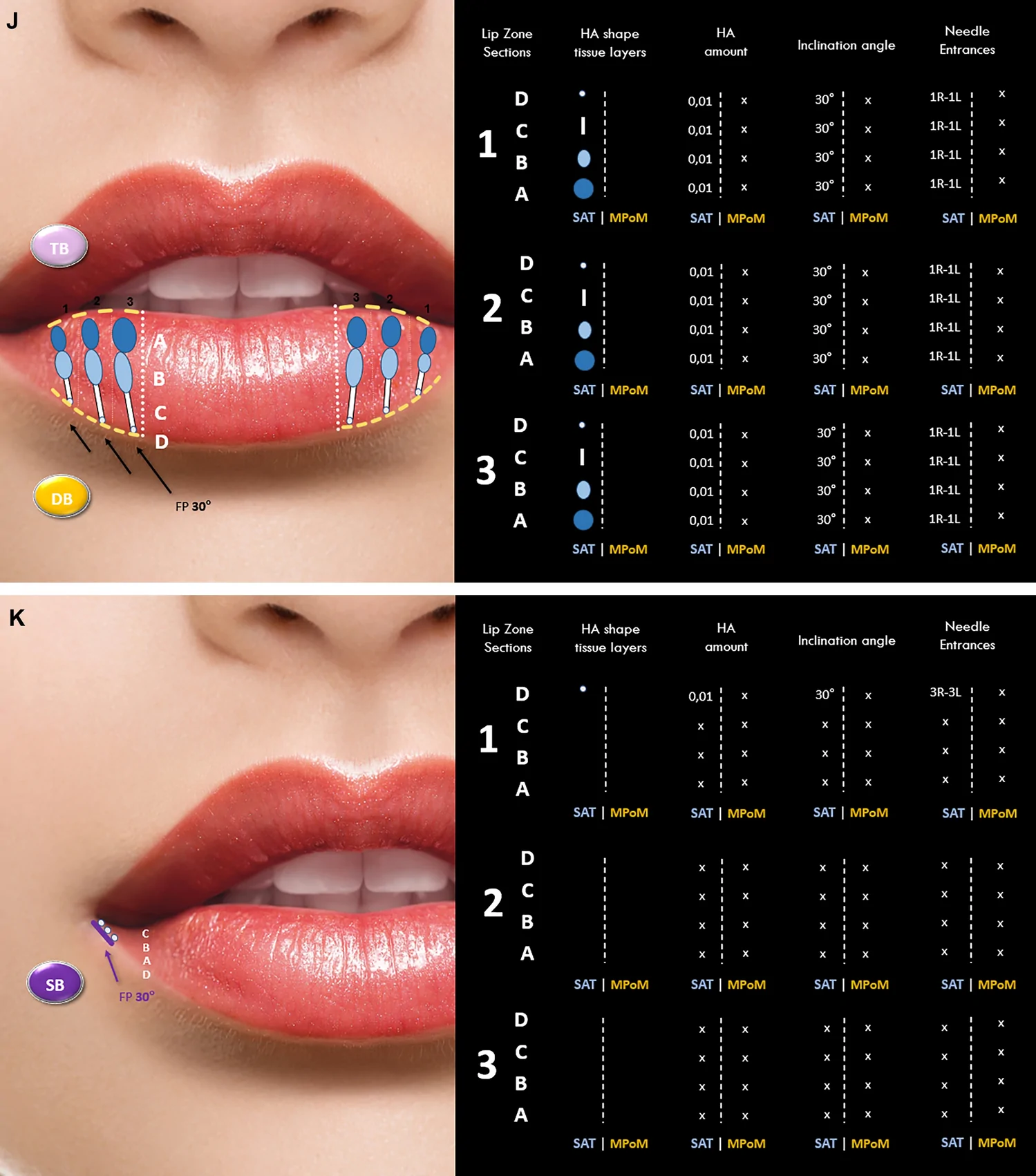
A 30-year-old female treated using the LA VISION Method: Hydration with reduction of gummy smile. Parts **A–D **show the patient before and after treatment. Part **E** summarizes the workflow performed using the LA VISION Method. Parts **F**–**K** show the strategic injection protocol. *ARC* Arc Border, *BB* Base Border, *CB* Climb Border, *CH* Cupid Hill, *CP* Cupid Peak, *DB* Drop Border, *FB* Fender Border, *FP* frontal plane, *HA* hyaluronic acid, *HB* Hill Border, *LV LEADS* Lip Visual Linear Elements for Aesthetic Design and Symmetry, *L* left, *MPoM* marginal portion of orbicularis oris muscle, *OB* Oval Border, *R* right, *SAT* subareolar tissue, *SB* Sign Border, *SP* sagittal plane, *TB* Top Border

**Fig. 9:**
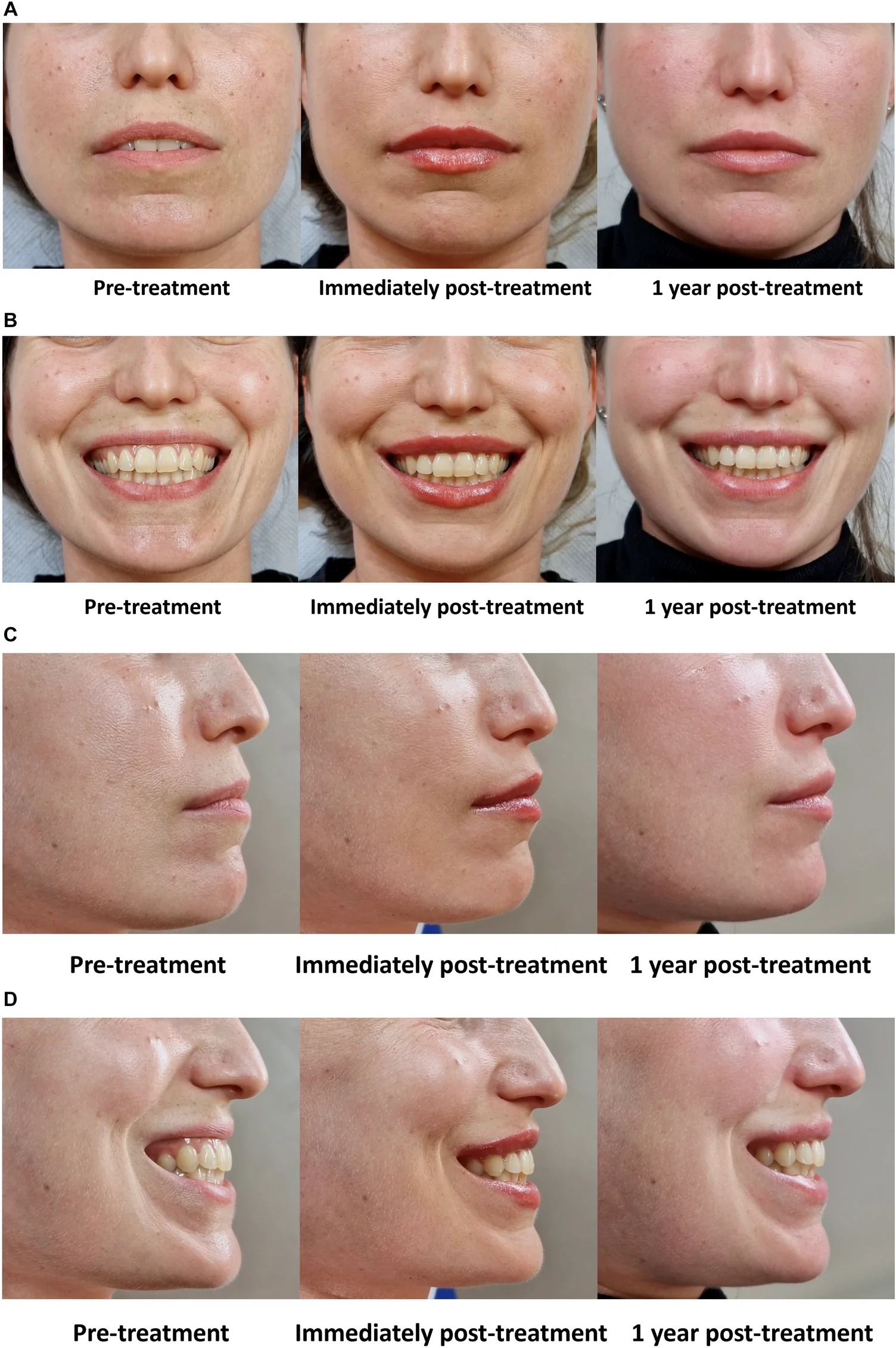

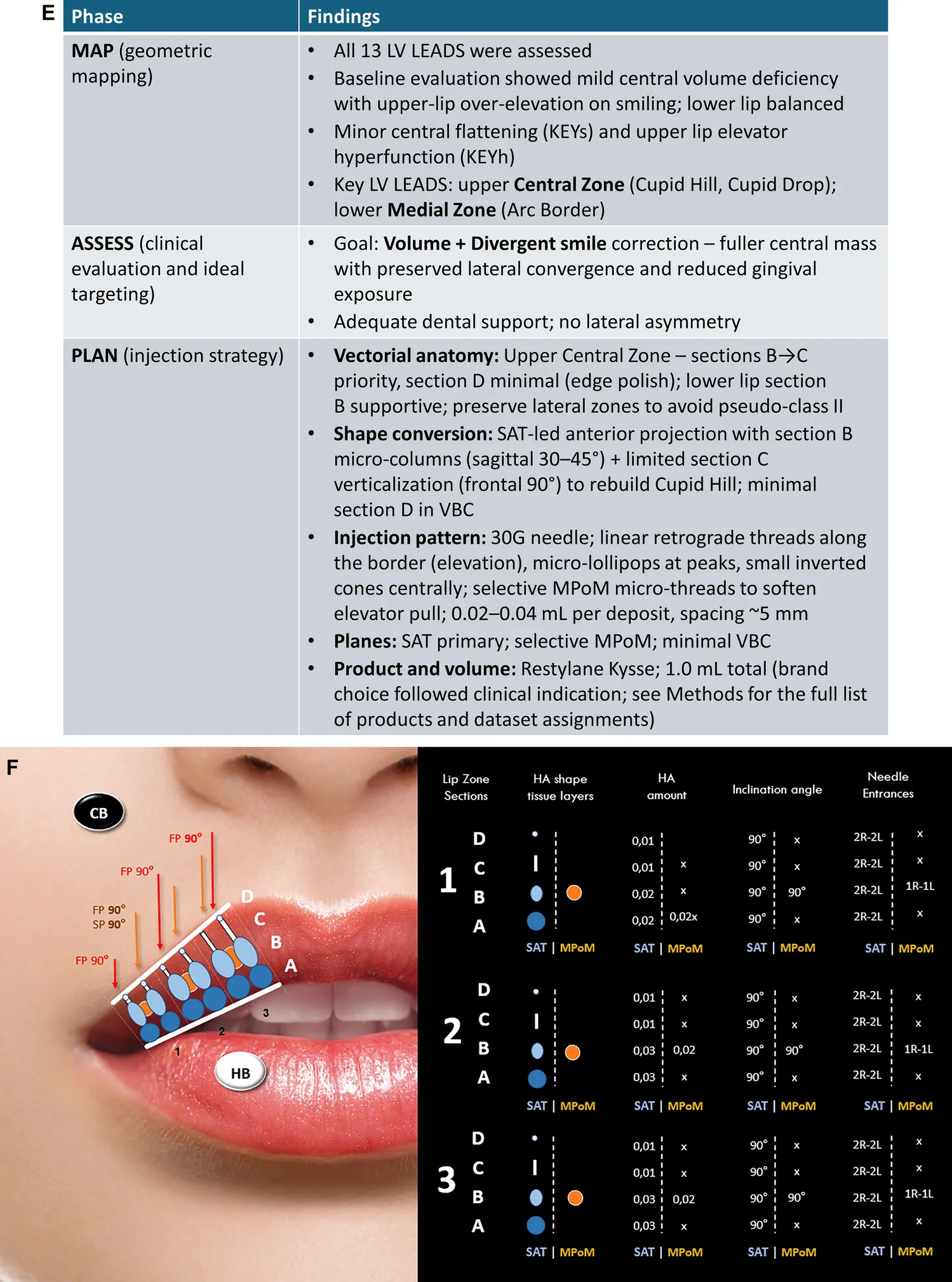

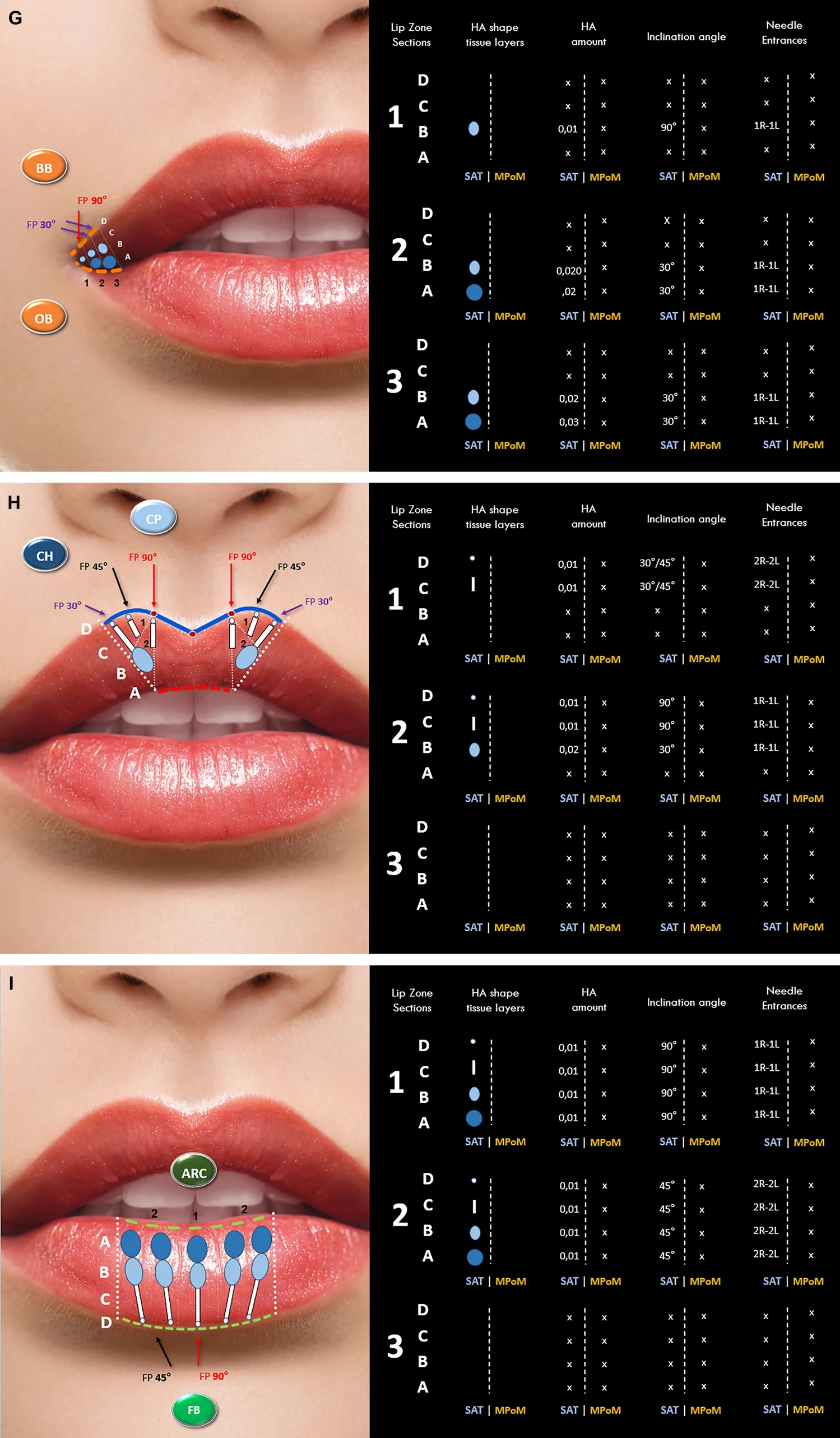

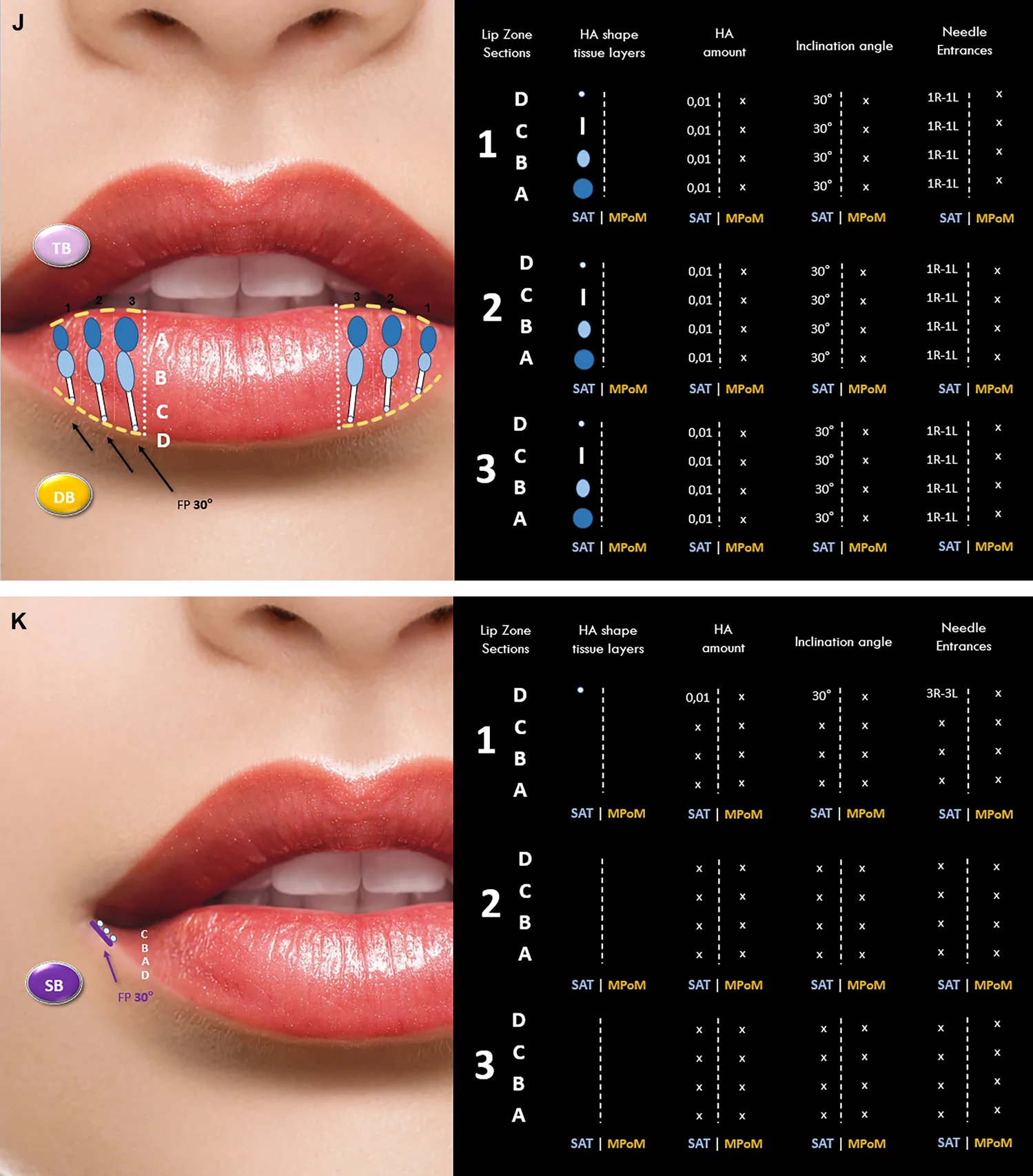
A 37-year-old female treated using the LA VISION Method: Volume enhancement with gummy smile correction. Parts **A**–**D** show the patient before and after treatment. Part **E** summarizes the workflow performed using the LA VISION Method. Parts **F**–**K** show the strategic injection protocol. *ARC* Arc Border, *BB* Base Border, *CB* Climb Border, *CH* Cupid Hill, *CP* Cupid Peak, *DB* Drop Border, *FB* Fender Border, *FP* frontal plane, *HA* hyaluronic acid, *HB* Hill Border, *LV LEADS* Lip Visual Linear Elements for Aesthetic Design and Symmetry, *L* left, *MPoM* marginal portion of orbicularis oris muscle, *OB* Oval Border, *R* right, *SAT* subareolar tissue, *SB* Sign Border, *SP* sagittal plane, *TB* Top Border, *VBC* virtual border canal

Example cases are provided in Figs. 7, 8, 9. Figure 7 shows a 25-year-old female who presented with a desire for fuller lips, prioritizing central enhancement of both lips. She had no relevant medical history and no functional complaints. The MAP phase showed mild central vermilion deficiency with blunted Cupid Peaks; a naturally straight lower lip; and an upper-to-lower lip ratio that was slightly reduced centrally. Key LV LEADS were in the upper Central Zone (Cupid Hill, Cupid Peak, Cupid Drop), and lower Medial Zone (Arc Border, Fender Border). Lateral convergence was preserved and the commissures were neutral. Within the ASSESS phase, the ‘Mind consultation’ categorized the request as ‘Volume’ with a ‘natural’ aesthetic target, and the ‘Individual analysis’ confirmed adequate dental support, normal smile dynamics, and no asymmetry. Her ‘Ideal status’ targeted crisp Cupid Peaks with gentle M-shaped Cupid Drop and an elliptical lower Arc Border. For algorithmic execution in the PLAN phase, the product selected was Stylage M (Vivacy), matching structure with blendability, and injected using a 30G needle. ’Shape conversion’ was based on entry at the vermilion border with vertical fences every 5 mm. In the upper Central Zone, section B (anterior projection) was injected with micro-columns into the SAT, using a frontal 45° inclination (parallel lift) and a sagittal 30–45° inclination to modulate depth; in section C (verticalization), selective 90° micro-columns were injected for Cupid Hill elevation; and in section D (edge definition), there was minimal sculpting in the VBC (≤ 0.01 mL per fence). In the lower Medial Zone, sections B→C, micro-columns were injected in the SAT for soft counterbalancing of upper projection. For the ‘Vectorial anatomy’ and ‘Placing pattern’ components, placement was predominantly in the SAT, with no intramuscular injection required. The ‘Dose pattern’ was linear retrograde along the border for elevation, with inverted cone micro-deposits centrally to project without bulk. Thus, the technical parameters from the planning grid were:

- Inclination angles: frontal plane 90° (verticalization), frontal plane 45° (anterior projection), frontal plane 30° (fine adjustment);
- Sections addressed: Central Zone section B–C primary; section D minimal; lower lip section B–C supportive;
- Tissue layers: SAT dominant; VBC minimal;
- Amount of HA: 0.7 mL total (distributed micro-volumes ≤ 0.04 mL per fence).

Immediately post-treatment, the patient’s lips showed enhanced central volume, sharper Cupid architecture, balanced upper-to-lower lip ratio, and natural lateral flow. At 12 months, the soft central fullness was maintained with minimal resorption, no irregularities, and high patient satisfaction. There were no AEs. This case demonstrates the algorithm for central deficiency with volume—prioritizing section B projection, then section C verticalization, and finishing with minimal section D edge work. Needle-only shape conversion ensured crisp control with small volumes.

Figure 8 shows a 30-year-old female who requested improved lip hydration and a reduction in gingival display on smiling, without increasing overall volume. The MAP phase showed adequate global volume with surface dryness; moderate dynamic gingival display; and intact central architecture. Key LV LEADS were in the upper Central Zone (Cupid Drop), and lower Medial Zone (Arc Border). The perioral skin showed mild texture changes. In the ASSESS phase, the ‘Mind consultation’ categorized the request as ‘Hydration’ + ‘Divergent smile,’ and the ‘Individual analysis’ indicated normal occlusion, upper lip elevator hyperfunction during smiling, and preserved symmetry. The ‘Ideal status’ targeted maintained size and shape with subtler incisor show and supple texture. In the PLAN phase, the selected HA filler was Juvéderm Ultra 3 (AbbVie), allowing structure and glide based on a dual-plane strategy, injected using a 30G needle. ‘Shape conversion’ was based on upper lip central micro-columns in section B to create anterior ‘cushioning’ and reduce incisor exposure, as well as limited section C verticalization to resist superior retraction. Frontal inclinations were 45°/90° as needed, and sagittal inclinations were 30–45° to engage SAT and selective MPoM for mild myomodulatory effects. The ‘Vectorial anatomy’ component was based on split-plane placement: SAT (hydration/support) and targeted MPoM threads (smile softening). Overfilling of section D was avoided to preserve light reflex. The ‘Placing pattern’ employed was linear retrograde threads for border support and micro-bolus in section B for anti-retraction buffer. Thus, the technical parameters in the planning grid were:

- Inclination angles: frontal plane 90° (vertical lift), frontal plane 30° (fine anterior adjustment), frontal plane 45° (projection);
- Sections addressed: upper Central Zone section B–C; lower Medial Zone supportive;
- Tissue layers: SAT and MPoM dual plane;
- Amount of HA: 1.0 mL total (micro-columns and micro-bolus distribution ≤ 0.04 mL per fence).

Immediately post-treatment, the patient showed improved turgor and reduced gingival display, and the dynamic smile was softened without apparent bulk. At 12 months, she had maintained hydration and function, natural behavior, and no unwanted volumization. No AEs were recorded. Overall, the ‘Hydration’ + ‘Divergent smile’ algorithm favors SAT cushioning and selective MPoM engagement to modulate elevator pull while keeping volume neutral. Controlled angles and micro-volumes are critical.

Figure 9 shows a 37-year-old female who presented seeking increased volume with concomitant correction of a gummy smile. Baseline examination showed mild central flattening and upper lip elevator hyperfunction on smiling. The MAP phase revealed a mild central deficit in the upper lip (Cupid system flattened), with a balanced lower lip. There was dynamic over-elevation of the upper lip on smiling. Key LV LEADS were in the upper Central Zone (Cupid Hill, Cupid Drop) and the lower Medial Zone (Arc Border). In the ASSESS phase, the ‘Mind consultation’ placed her goals as ‘Volume’ + ‘Divergent smile.’ The ‘Individual analysis’ showed adequate dental support and no lateral asymmetry, and her ‘Ideal status’ was characterized as a fuller central mass with preserved lateral convergence and reduced gingival exposure. In the PLAN phase, the product selected was Restylane Kysse (Galderma) for elastic support with dynamic integration, injected using a 30G needle. ‘Shape conversion’ in the upper lip was based on section B (primary) micro-columns for anterior projection in the SAT, with sagittal angle 30–45°; section C (secondary) verticalization with frontal inclination of 90° to re-establish Cupid Hill; and minimal section D in the VBC for edge polish. Selective MPoM threads injected centrally were used to reduce superior retraction (smile). In the lower lip, light section B SAT support was provided to maintain upper-to-lower lip harmony. The ‘Placing pattern’ used a combination of linear retrograde threads (border elevation), micro-lollipops at peaks, and small inverted cones centrally. Volume distribution was biased upper centrally, and the lateral zones were preserved to avoid pseudo-class II. The technical parameters from the planning grid were:

- Inclination angles: frontal plane 90° (vertical lift for Cupid Hill), frontal plane 45° (anterior projection), frontal plane 30° (fine anterior adjustment);
- Sections addressed: upper Central Zone section B–C priority; section D minimal; lower lip section B supportive;
- Tissue layers: SAT, selective MPoM, and VBC edge polish;
- Amount of HA: 1.0 mL total (micro-columns and micro-bolus ≤ 0.04 mL per fence)

Immediately post-treatment, the patient’s lips showed increased central volume with harmonious ratio and visible reduction in gingival display. At 12 months, she had stable central volume, preserved hydration, and persistent dynamic improvement. There were no significant AEs. This case illustrates a ‘Volume’ + ‘Divergent smile’ algorithm, based on SAT-led projection/verticalization coupled with focused MPoM modulation. Respecting lateral flow and limiting section D injection avoids unnatural protrusion and maintains natural convergence.

## Discussion

The LA VISION Method offers a systematic, visual, logic-based, and repeatable approach to lip analysis and injection planning based on HA fillers. As the field of aesthetic medicine moves toward greater scientific rigor, methodological frameworks for codified analysis and planning are likely to become a key foundation of best practice. LA VISION is not a theoretical abstraction detached from clinical practice but rather a formalized decision-making grammar derived from (and iteratively refined through) extensive longitudinal clinical application. It provides a structured framework that can be critically evaluated, cited, and refined within the scientific literature. This may be particularly important with nonsurgical lip augmentation given both its popularity worldwide and the anatomical complexity of the area. Many different clinical approaches have been published [18, 24–30], and each yields excellent outcomes, but they are primarily technique-centered rather than planning-focused.

The LA VISION Method emphasizes structured planning and individualized morphology, but it does not reject the value of existing injection techniques. On the contrary, most of the established techniques can be reframed through the LA VISION lens as specific *technical gestures*—defined movements with precise entry points and inclination angles—designed to achieve a certain shape modification. However, these are only effective when matched with the appropriate anatomical context and lip morphology. What is commonly referred to as a ‘technique’ should therefore be understood as a fixed codification of selected parameters within the PLAN phase (such as vector orientation, injection depth, and filler behavior) rather than a universally applicable strategy. Indeed, while a given technique may work well in specific cases, it cannot be applied generically to all lips or all aesthetic goals. Thus, these techniques may be considered as fixed applications within the PLAN phase, while the system itself provides the adaptable strategy that ensures individualized treatment.

The method delivers a modular matrix that can be adapted to individual patients through anthropometric division (MAP phase), visual–functional diagnosis (ASSESS phase), and stepwise procedural planning (PLAN phase). A key innovation lies in the definition of six lip zones and the classification of 13 visual aesthetic lines and points (LV LEADS). By anchoring the mapping process to individualized facial landmarks rather than fixed anatomical coordinates, the LA VISION Method reconciles structural consistency with personalization. Its strength lies in the use of a shared decision structure rather than the imposition of uniform measurements, thereby allowing individual, cultural, and generational variation to be preserved within a common clinical framework.

Operational terminology of this type is commonly employed in clinical decision systems to bridge perception, communication, and technical execution, without implying a redefinition of anatomical structures. Within this context, the use of shape logic (e.g., curved, straight, mixed) and the definition of ideal morphological targets for each of the LV LEADS provide a clinically oriented vocabulary that is both visually intuitive and anatomically grounded.

This structured coding system facilitates clear communication among clinicians and supports consistent treatment planning, contributing to the development of shared best practices in aesthetic medicine. For clinicians, regardless of experience level, the LA VISION Method provides a complete procedural algorithm for lip shape assessment in the context of face proportion variables (‘Individual analysis’) and lip shape morphological endpoints (‘Ideal status’). This is then connected to the technical aspect, to ensure control of *where* HA filler is placed (zone, LV LEAD, and section [A–D]), *how* it is deposited (needle angle), *how much* filler is used (volumetry per section), and *with what* product (rheology-based decision-making). This creates a multidimensional injection logic that can be adjusted according to individual patient morphology, dynamic movement, and aesthetic goals. Indeed, the method accommodates all cultural, generational, and gender variations by providing a flexible aesthetic language rather than a one-size-fits-all template. It respects both anatomical complexity and personal individuality.

In parallel with the rise of nonsurgical treatment techniques, the increasing influence of social media has contributed to widespread dissemination of visual standards and aesthetic trends that are often exaggerated, short-lived, or poorly contextualized. This may affect patient perceptions and create a distorted sense of what is ‘normal’ or desirable. As a result, many patients present with requests that are heavily influenced by online interactions rather than by personal anatomical or individual harmony. In turn, clinicians are then placed in the difficult position of either reinforcing these trends or resisting them, with a resulting risk of dissatisfaction or misunderstanding. The LA VISION Method offers a crucial counterbalance. By proposing shape over volume, individuality over standardization, and proportionally adapted ideals over transformation-driven outcomes, it provides practitioners with a clear and structured tool for designing lip treatments that are elegant, personalized, and rooted in the fundamental principles of aesthetic medicine. Accordingly, the LA VISION Method is designed to guide the clinical reasoning process rather than to prescribe aesthetic outcomes. Deviations from reference configurations may be clinically appropriate when consciously chosen within shared decision-making. The method was developed through a progressive process of structured clinical observation and iterative refinement across a longitudinal case series. The reported cases are therefore not presented as post hoc validation of a pre-existing theoretical construct, but as the empirical substrate through which the decision framework was articulated, tested, and consolidated over time. Hence, the case material serves to make the internal logic, operability, and stability of the proposed decision grammar inspectable across heterogeneous clinical scenarios.

Crucially, within the current cohort, benefits were consistently achieved; stratified analyses showed comparable AE profiles and convergent PROM gains, irrespective of filler brand, injection device, or patient goal. AE rates declined over time and late-onset swelling remained ≤ 0.5% (0% at T1Y). Core PROM domains stabilized at values of ~8.0 at 6 months and ~7.6–7.8 at 12 months. These patterns support the idea that LV LEADS-based planning—rather than any specific product or device—was the primary driver of outcomes.

Consistent with current anatomical and ultrasound literature, reliance on surface landmarks alone cannot ensure vascular safety. In the LA VISION Method, safety is addressed through a combination of anatomical knowledge, cautious technique, conservative volumetric decisions, and plane awareness, rather than through presumed ‘safe zones.’ Only one case of clinically relevant palpability was observed across the entire cohort, which may reflect the framework’s emphasis on conservative volumetric decisions and appropriate plane selection.

It is important to note that the LA VISION Method originates from extensive hands-on experience in aesthetic medicine, accumulated through the treatment of a diverse, international patient population. Over time, the algorithm has been adopted by other clinicians, in both daily practice and teaching environments, owing to its perceived utility. The recommended injection volumes (‘Vector doses’) were derived from a quantitative analysis of gel occupancy within target tissues, empirically estimated from the volumetric distributions observed across zones and sessions. These parameters should be viewed as preliminary guidance, subject to adaptation based on the specific anatomical and aesthetic needs of each patient. Aesthetic outcomes cannot be mechanically reproduced by replicating ultra-specific injection points or micro-aliquots. The ‘Vector doses’ as 0.01 mL aliquots are intended to make clinicians aware of the decision space in which they are operating, rather than to prescribe a rigid technical execution. For less experienced clinicians, awareness that meaningful aesthetic modulation may occur at this scale encourages deliberate technical training prior to injection, thereby improving precision and safety. For more experienced practitioners, the same information supports refinement and conscious modulation of technique. In this context, reproducibility should be understood as reproducibility of clinical reasoning rather than as mechanical replication of technical gestures. Accordingly, the purpose of the clinical case series was not assessment of comparative efficacy, but rather to demonstrate the internal coherence, operability, and longitudinal stability of the proposed decision grammar across heterogeneous clinical scenarios.

It should also be noted that the PROMs used with the current cohort were pragmatic 0–10 instruments rather than validated scales, and while they were designed to mirror key constructs, formal psychometric testing is still needed. Further research, including controlled clinical trials and peer-reviewed studies, will be essential for rigorously assessing the reproducibility, safety, and effectiveness of this conceptual model. Integration with ultrasound guidance, three-dimensional imaging technologies, digital planning apps, and customized rheology selection tools may further enhance the method’s effectiveness.

## Conclusions

The LA VISION Method provides an integrated framework for aesthetic improvement of the lips using HA fillers, while preserving individual variation and cultural diversity. The structured three-phase workflow—MAP, ASSESS, PLAN—facilitates the transformation of subjective goals into anatomically grounded targets and reproducible planning and procedural logic. The method allows practitioners to analyze lip morphology in a modular and anatomically precise way; strategically plan injections based on desired shape and volume; and deliver controlled and personalized aesthetic results. In an initial cohort of 400 patients, LA VISION appeared to be safe and effective within the limits of an observational study and a demonstrative framework.

Achieving the highest levels of patient satisfaction requires a shift in treatment goals—moving beyond simple volumization or social media ‘lip trends’ toward a more comprehensive strategy that addresses individual morphology, anatomical ideals, and overall harmony. The sequencing logic embedded in LA VISION offers a layered methodology for creating a lip shape that is individual and ideal. This progression is designed to support a more nuanced and message-aware approach to HA filler-based aesthetic intervention.

## Supplementary Information

Below is the link to the electronic supplementary material.

Supplementary file1 (DOCX 21 KB)

Supplementary file2 (DOCX 28 KB)

Supplementary file3 (DOCX 28 KB)

Supplementary file4 (DOCX 17 KB)

Supplementary file5 (DOCX 32 KB)

Supplementary file6 (DOCX 29 KB)

Supplementary file7 (PDF 804 KB)

Supplementary file8 (PDF 1904 KB)
