# Supplementary material for: LA VISION©: A Structured Approach to Lip Analysis and Aesthetic Treatment with Hyaluronic Acid Fillers
Source: Aesthetic Plast Surg. 2026 Jul 1;50(15):6316–44. doi: 10.1007/s00266-026-05998-7 (PMC13493426; doi:10.1007/s00266-026-05998-7)
Supplement: Supplementary file 1 [file 266_2026_5998_MOESM7_ESM.pdf]

| LATERAL STATUS

© Copyright of Andrea Felice Armenti and Soave Holding srl socio unico

Vermilion Vector

© Copyright of Andrea Felice Armenti and Soave Holding srl socio unico

INDIVIDUAL ANALYSIS

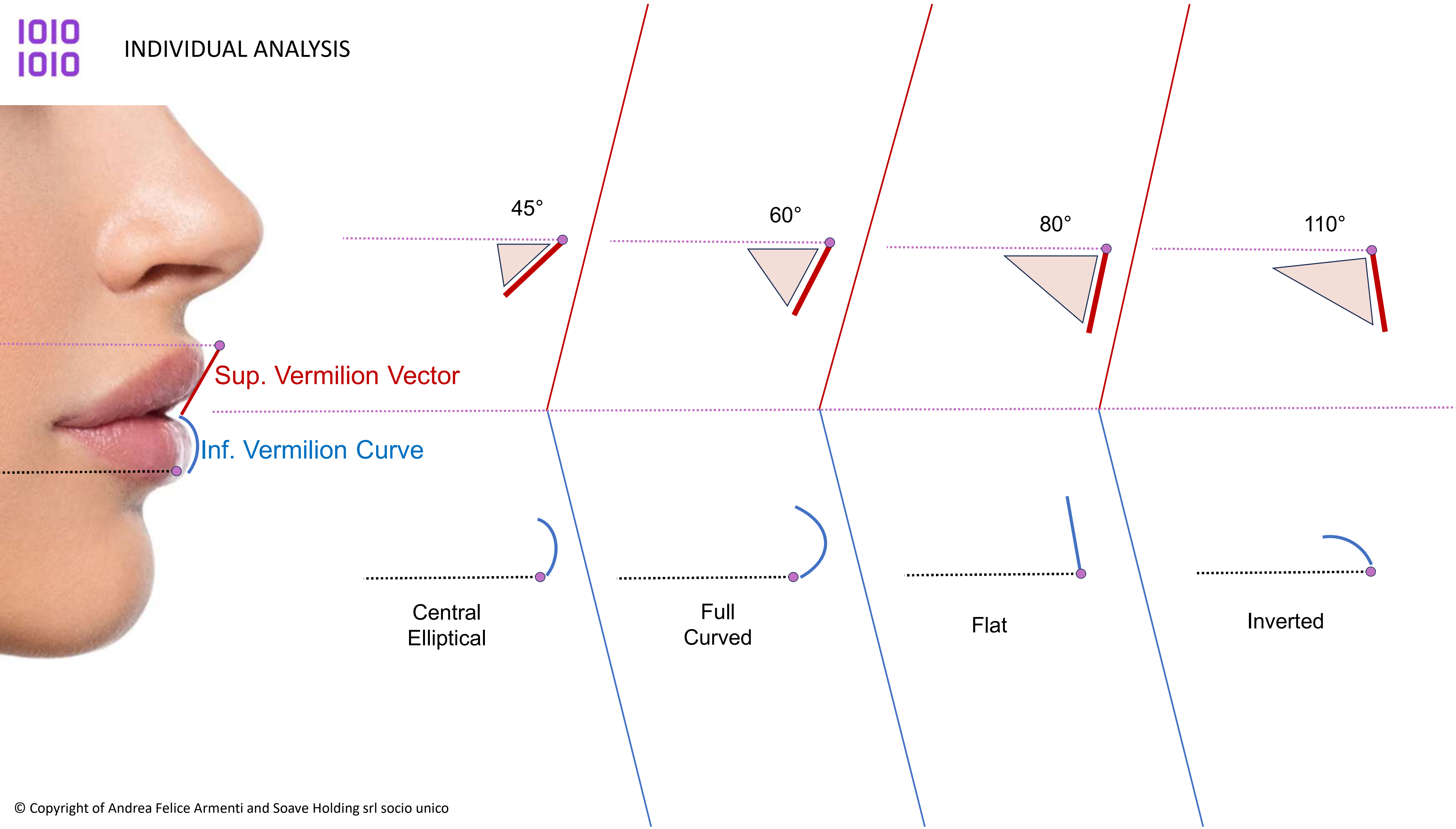

© Copyright of Andrea Felice Armenti and Soave Holding srl socio unico

INDIVIDUAL ANALYSIS

Profile Line

© Copyright of Andrea Felice Armenti and Soave Holding srl socio unico

INDIVIDUAL ANALYSIS

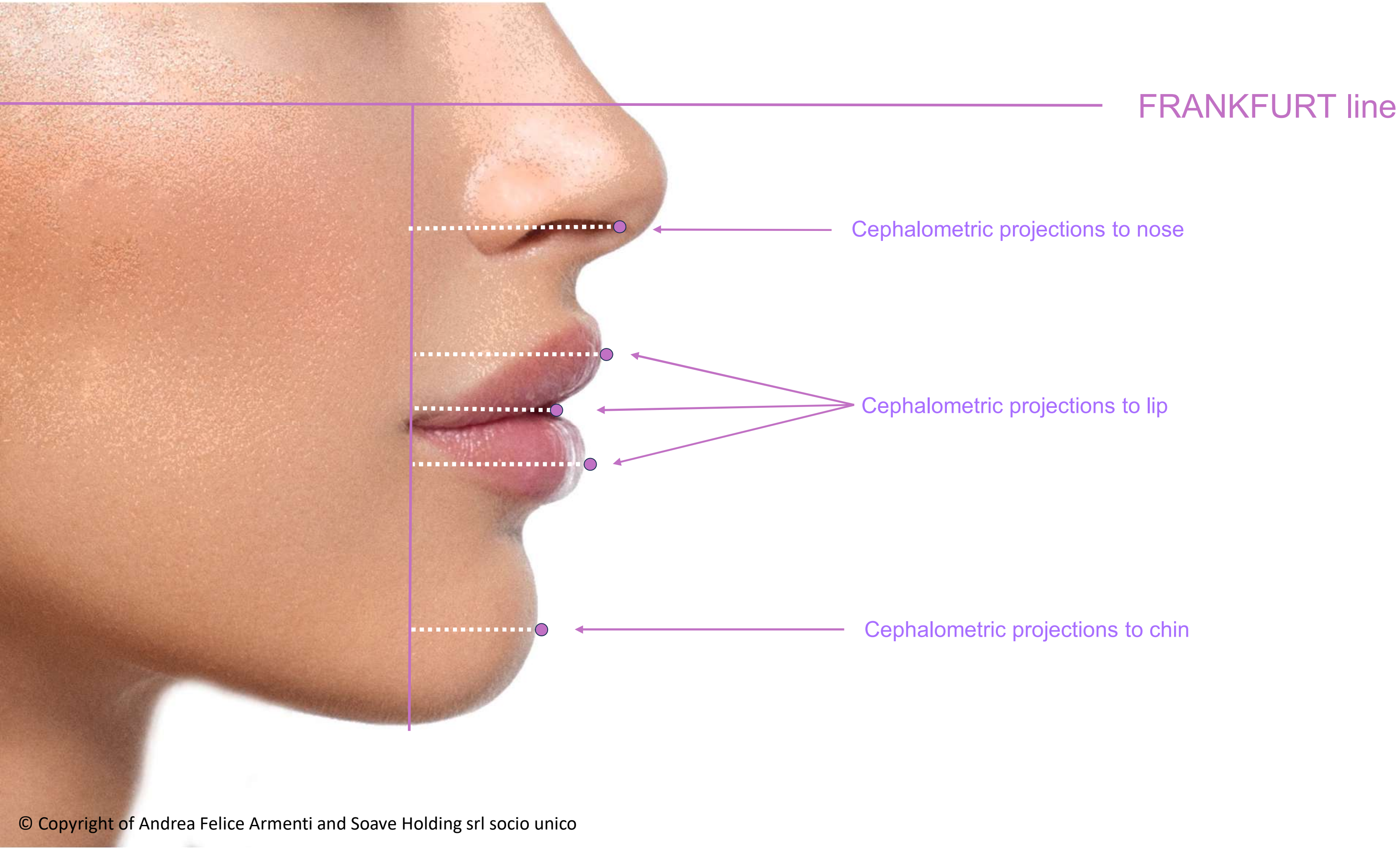

© Copyright of Andrea Felice Armenti and Soave Holding srl socio unico

INDIVIDUAL ANALYSIS

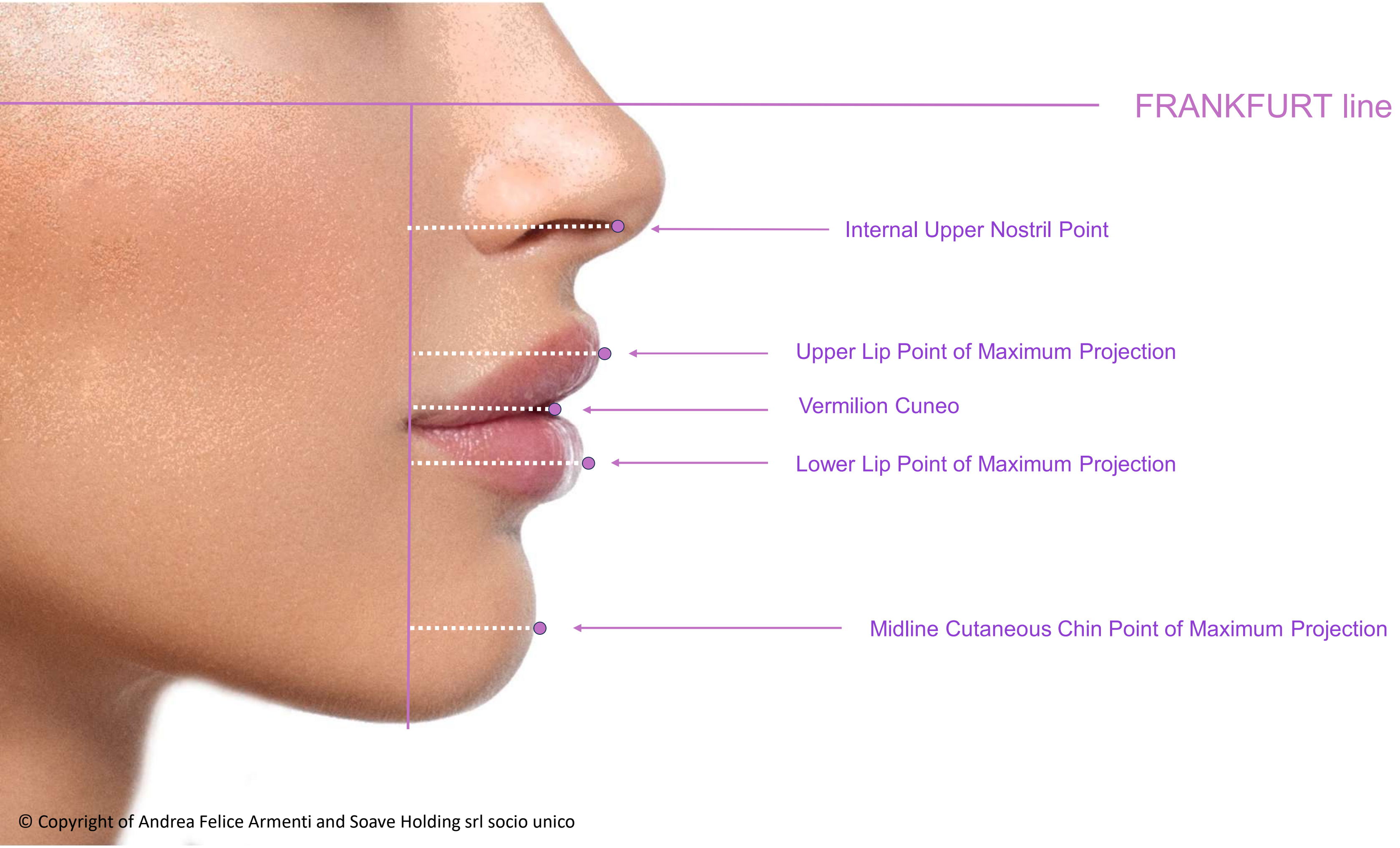

© Copyright of Andrea Felice Armenti and Soave Holding srl socio unico

INDIVIDUAL ANALYSIS

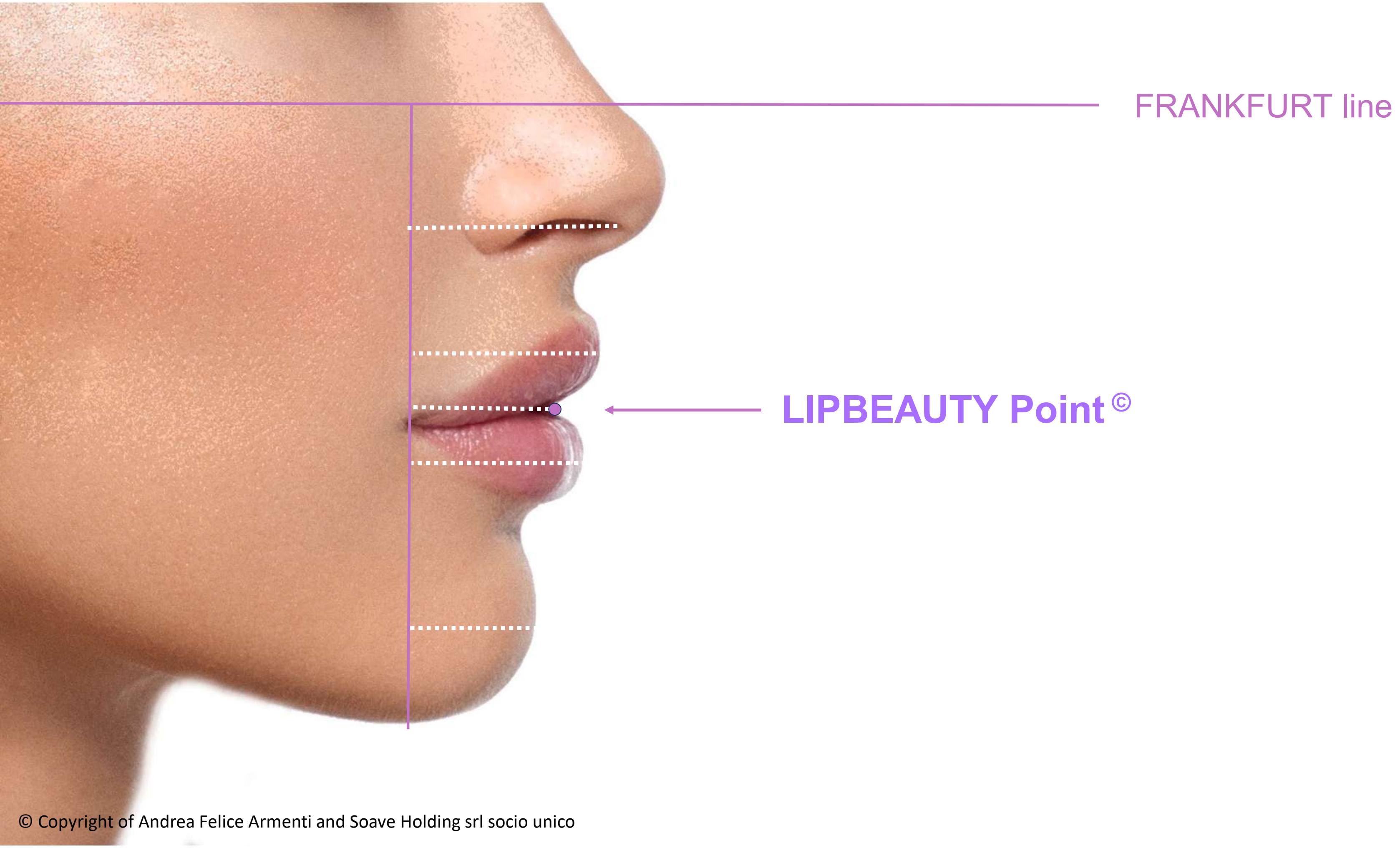

© Copyright of Andrea Felice Armenti and Soave Holding srl socio unico

INDIVIDUAL ANALYSIS

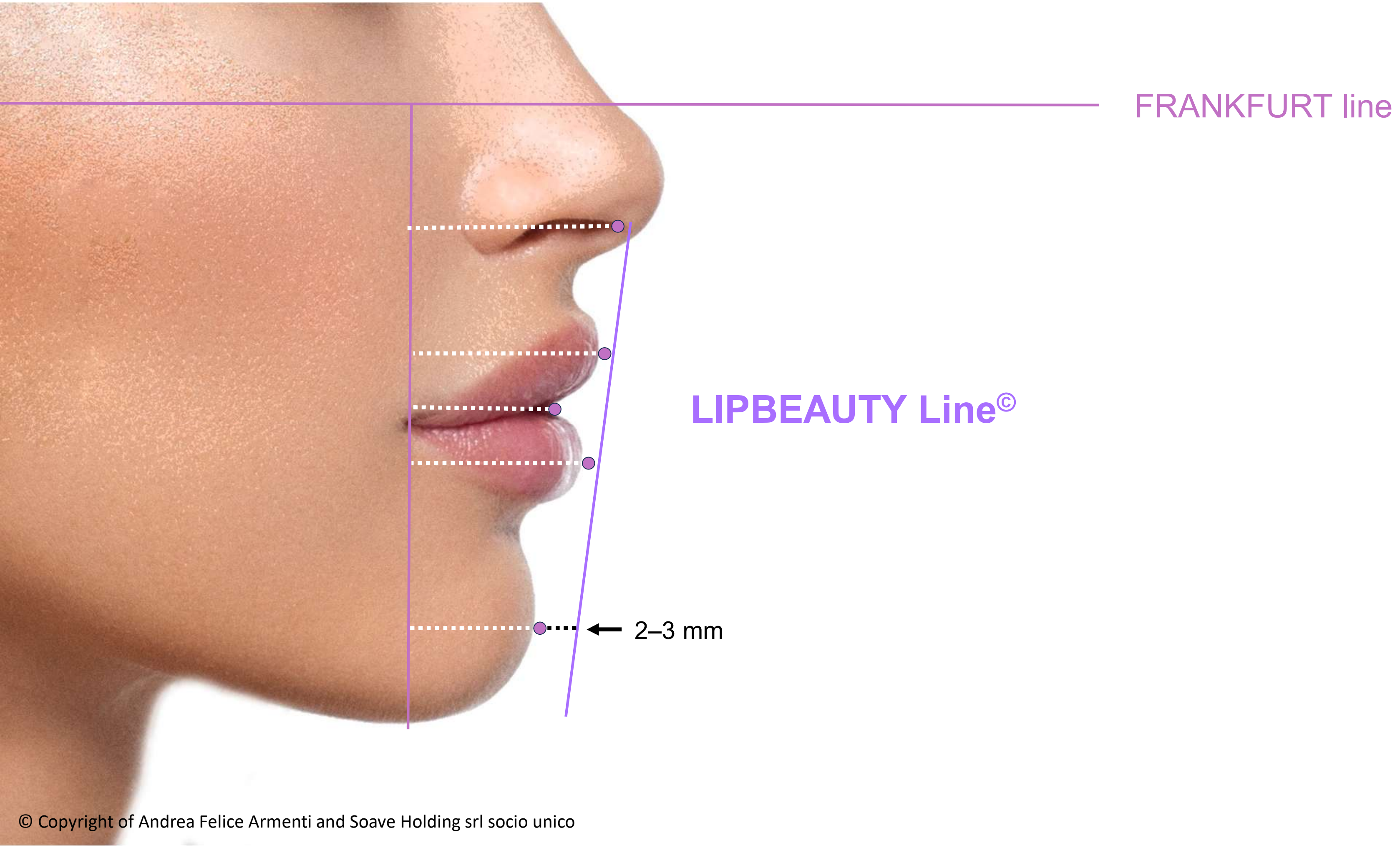

© Copyright of Andrea Felice Armenti and Soave Holding srl socio unico
