# Supplementary material for: LA VISION©: A Structured Approach to Lip Analysis and Aesthetic Treatment with Hyaluronic Acid Fillers
Source: Aesthetic Plast Surg. 2026 Jul 1;50(15):6316–44. doi: 10.1007/s00266-026-05998-7 (PMC13493426; doi:10.1007/s00266-026-05998-7)
Supplement: Supplementary file 2 [file 266_2026_5998_MOESM8_ESM.pdf]

This supplementary material presents a single illustrative example of the LV LEADS framework (Lateral Zone) to support interpretation of the method described in the main text. It is not intended as comprehensive training material.

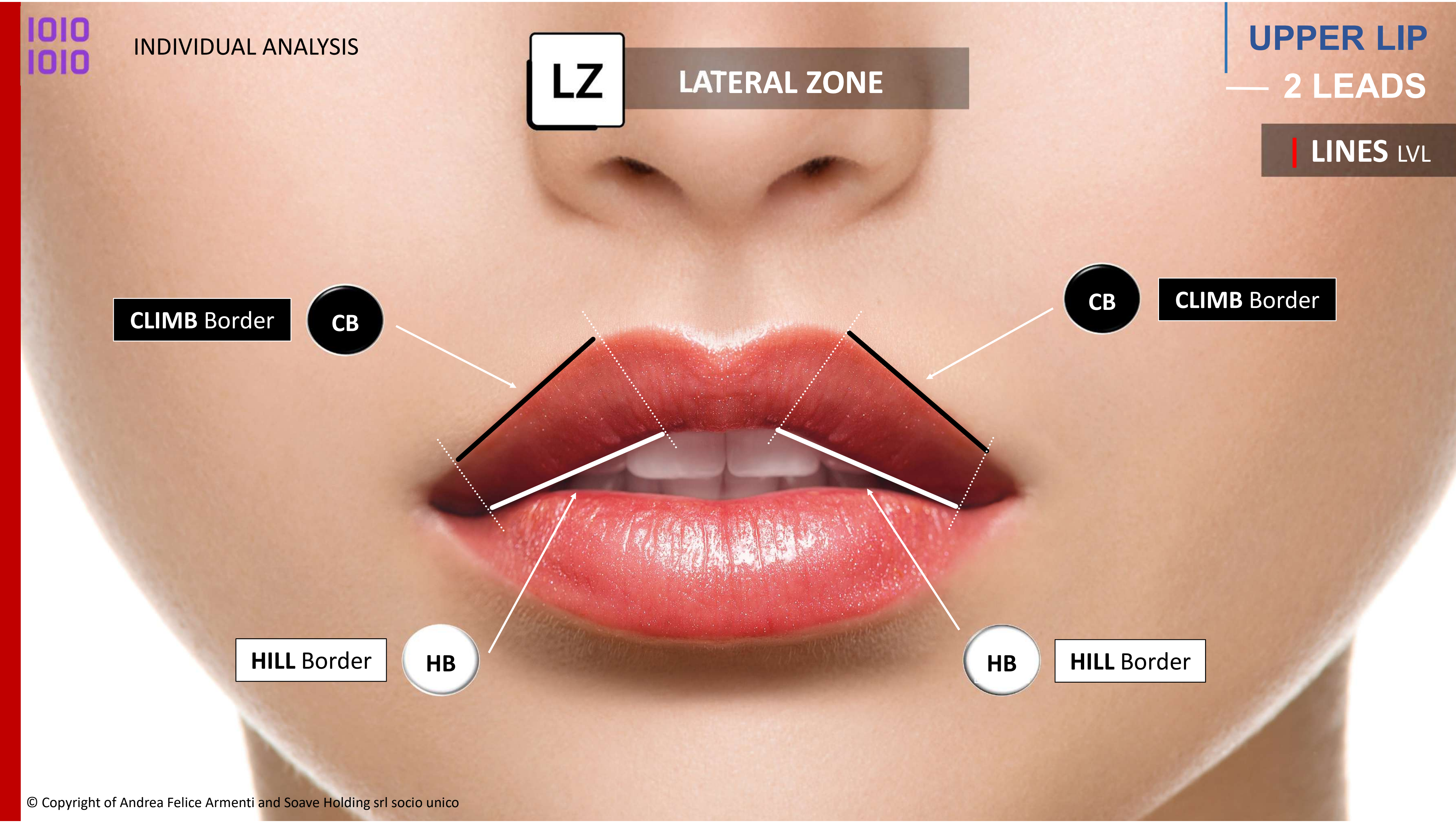

© Copyright of Andrea Felice Armenti and Soave Holding srl socio unico

INDIVIDUAL ANALYSIS

| LINES LVL

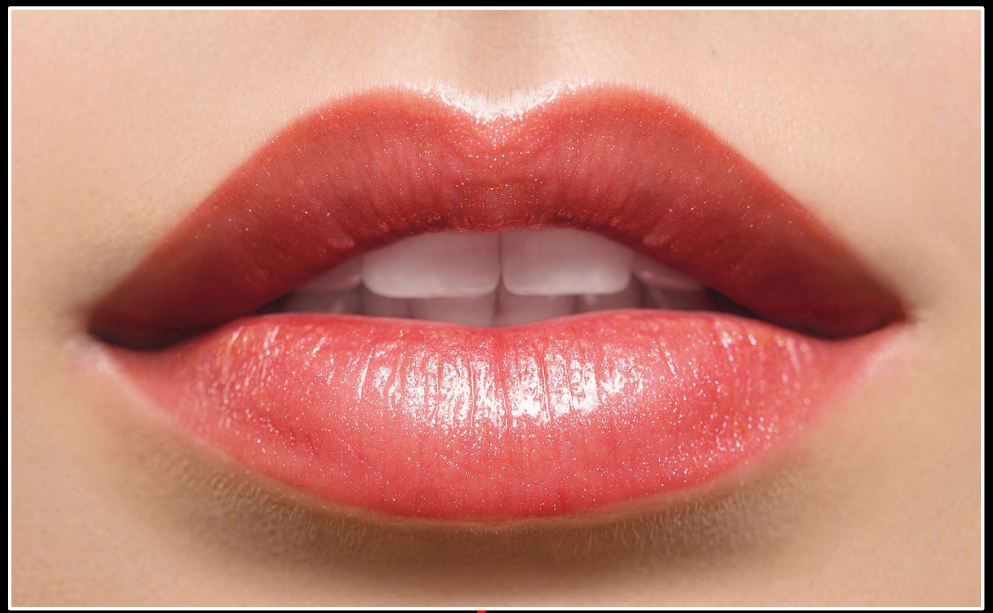

LIP ZONES

CB CLIMB Border

3 Types | 1 KEYs | 1 KEYh

© Copyright of Andrea Felice Armenti and Soave Holding srl socio unico

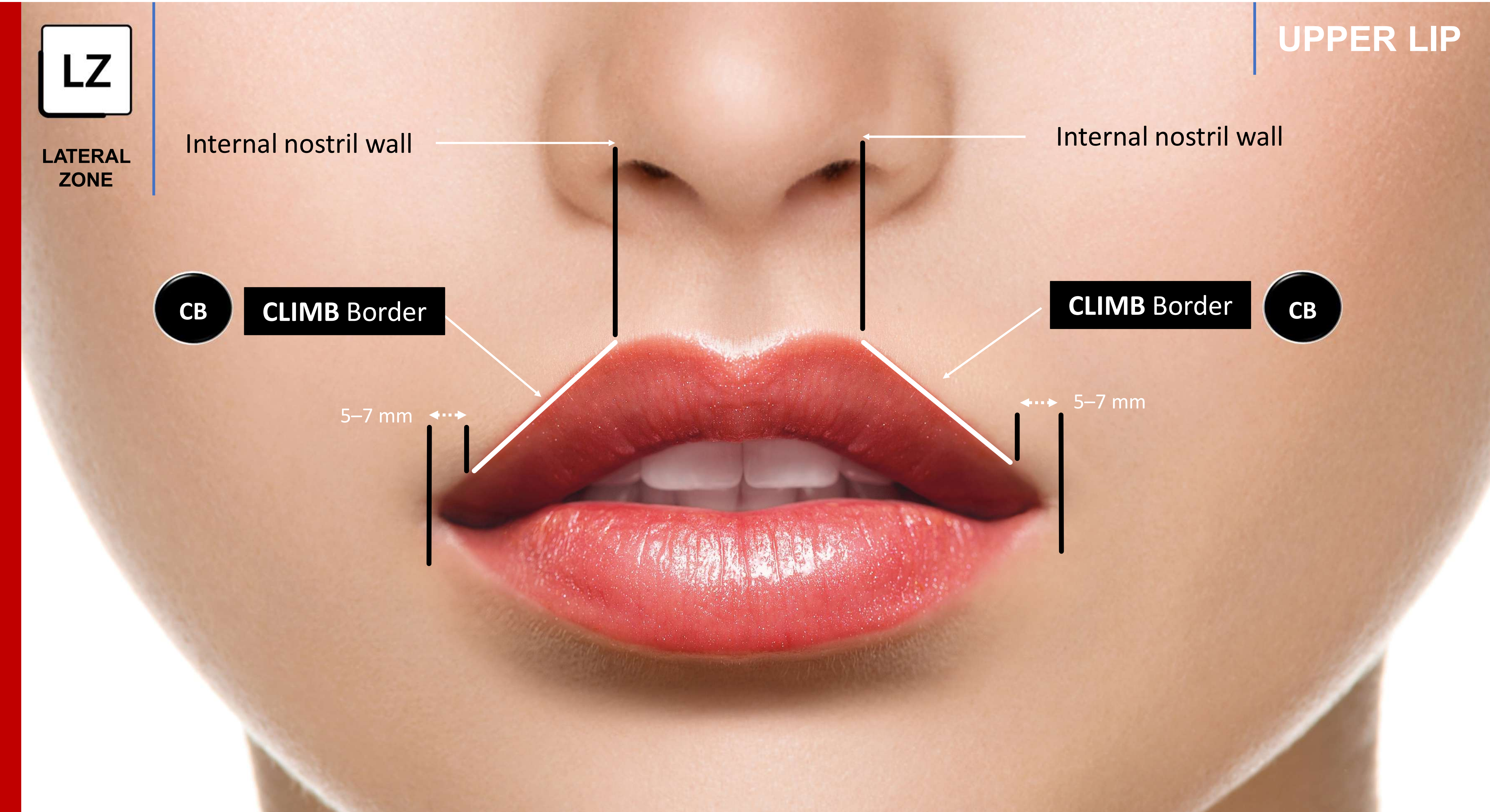

© Copyright of Andrea Felice Armenti and Soave Holding srl socio unico

INDIVIDUAL ANALYSIS

Types CB CLIMB Border

1 type

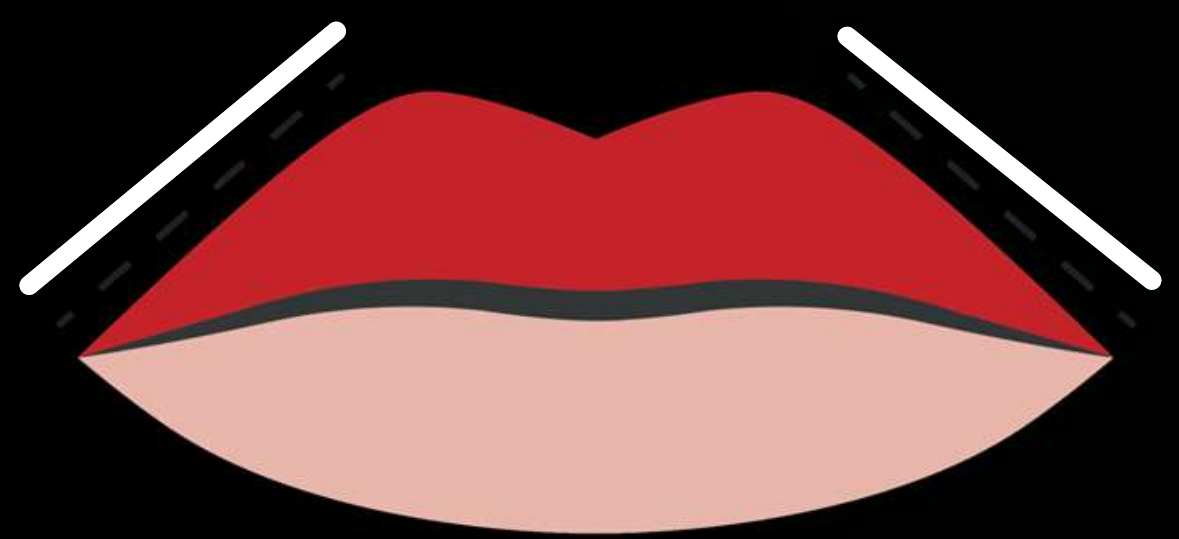

STRAIGHT

2 type

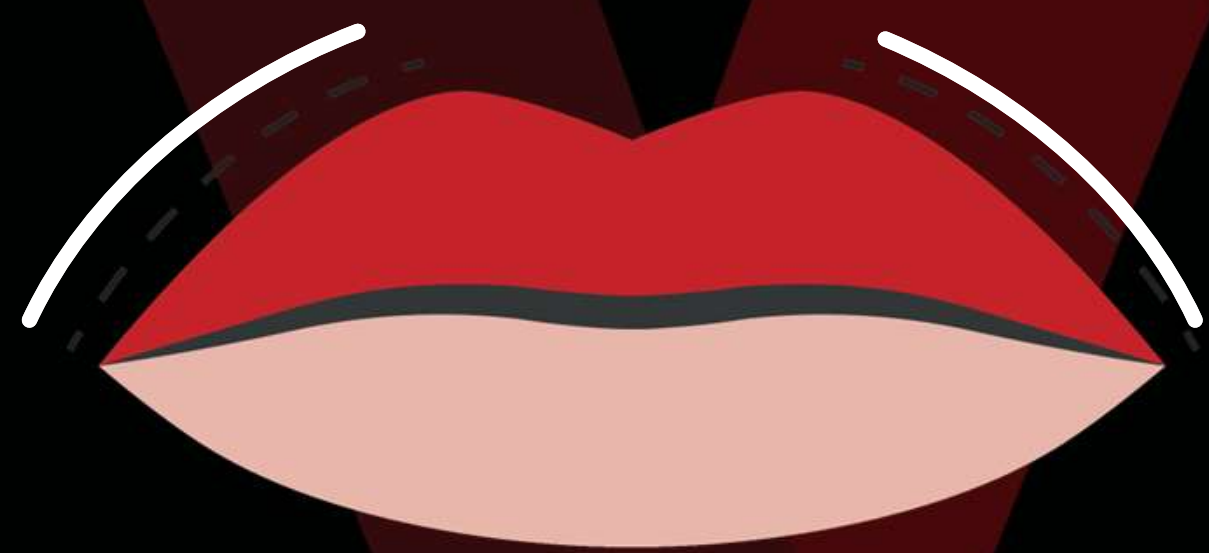

CURVED

3 type

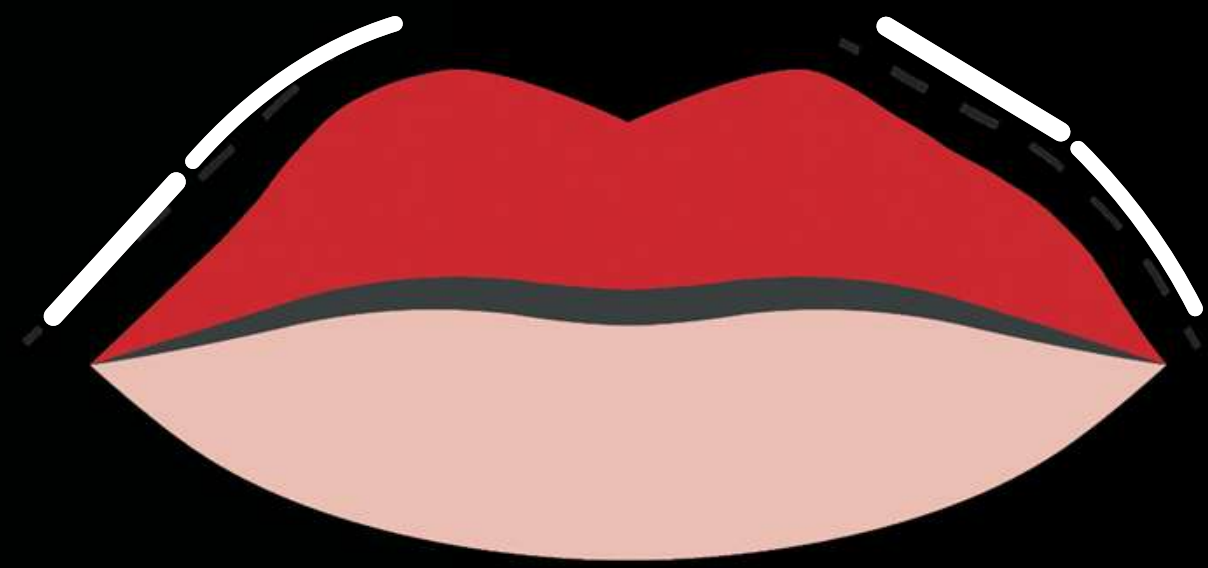

MIXED

Types

© Copyright of Andrea Felice Armenti and Soave Holding srl socio unico

Types CB **CLIMB** Border

1 type

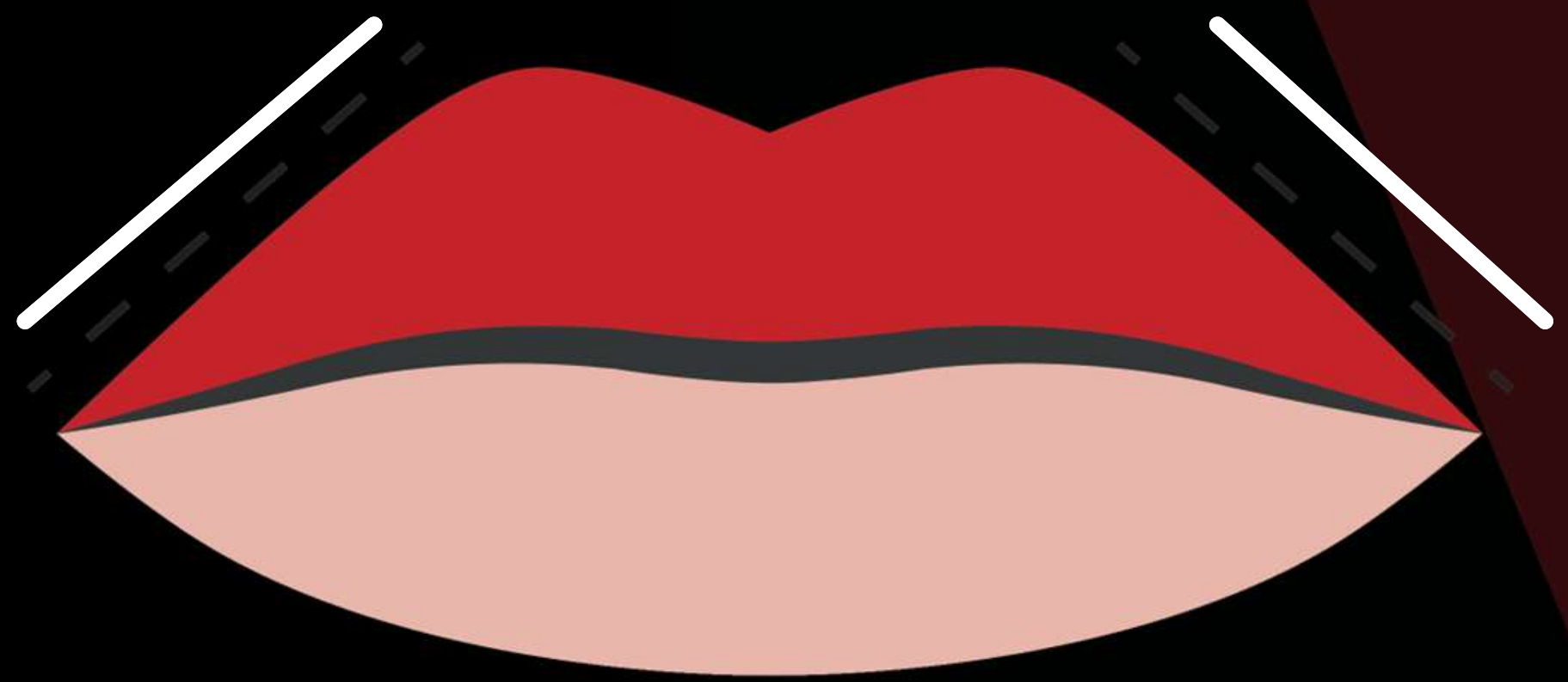

STRAIGHT

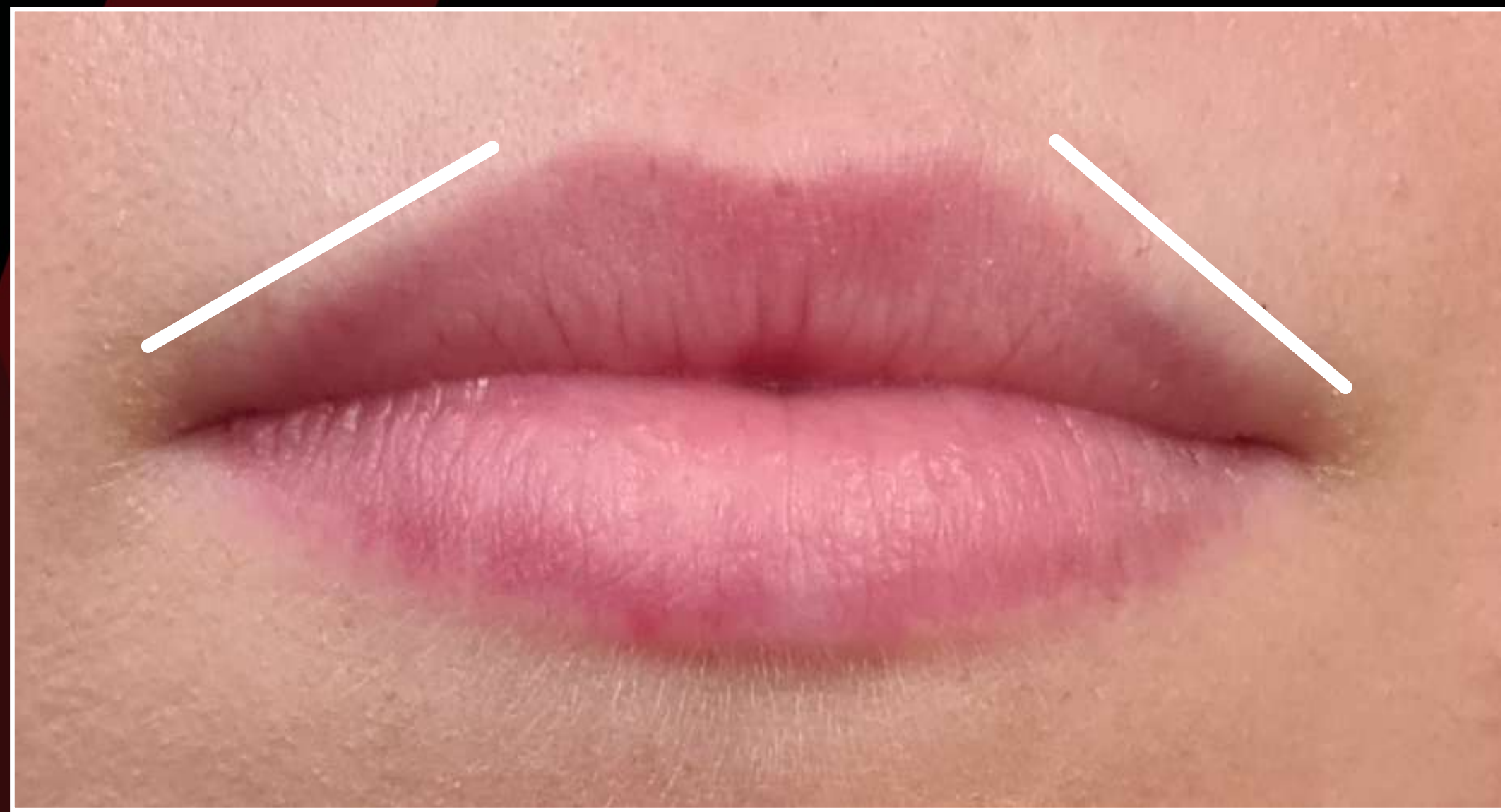

Types

© Copyright of Andrea Felice Armenti and Soave Holding srl socio unico

INDIVIDUAL ANALYSIS

Types CB **CLIMB** Border

2 type

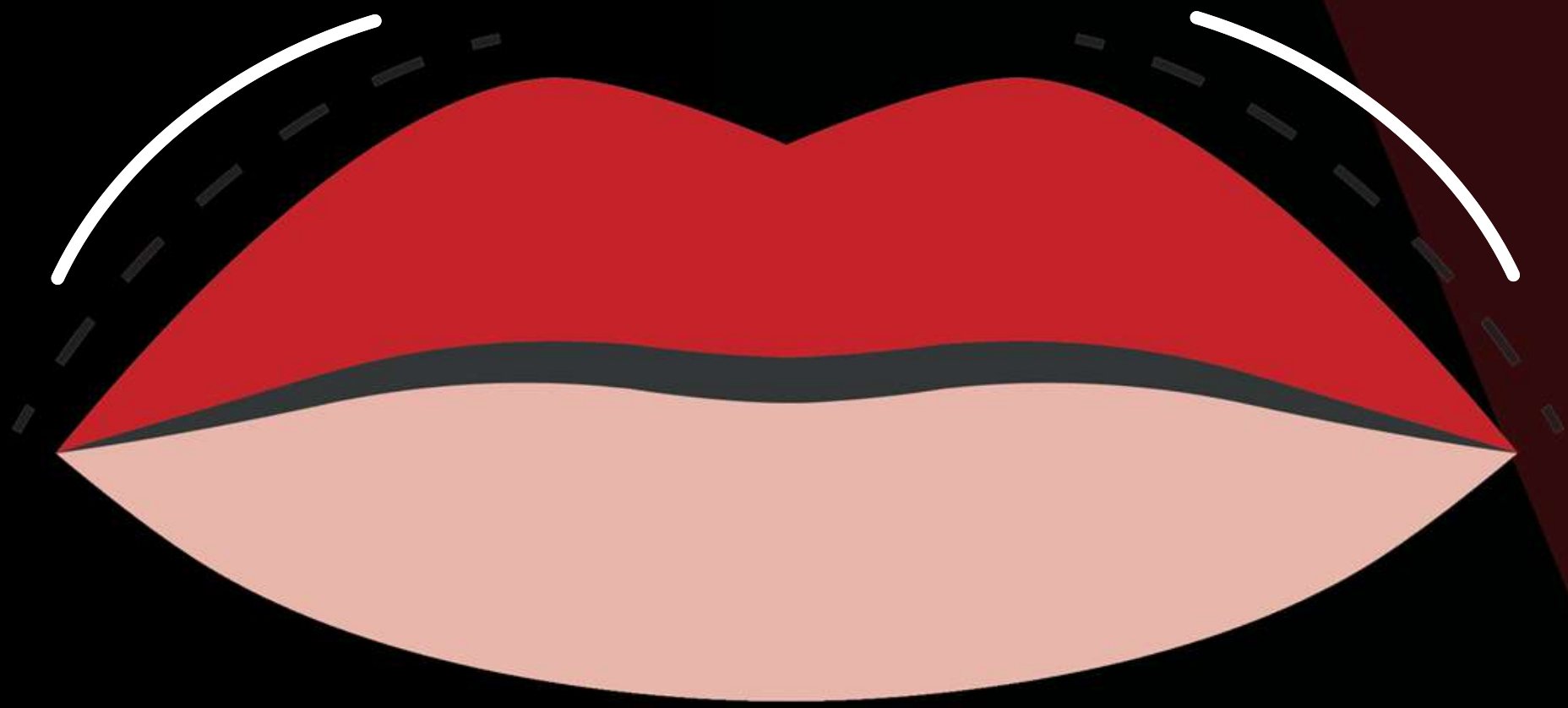

CURVED

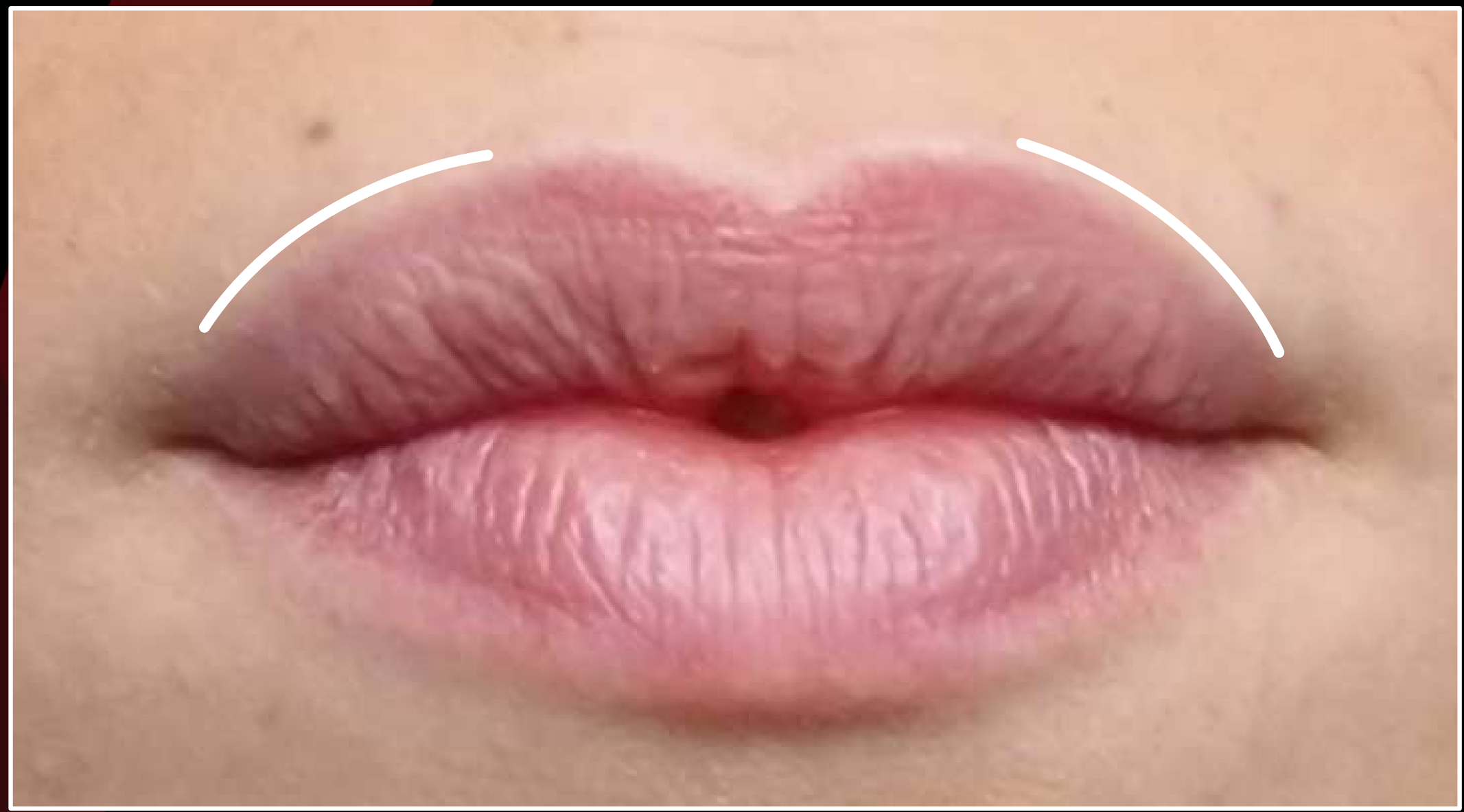

Types

© Copyright of Andrea Felice Armenti and Soave Holding srl socio unico

INDIVIDUAL ANALYSIS

Types **CB** **CLIMB** Border

3 type

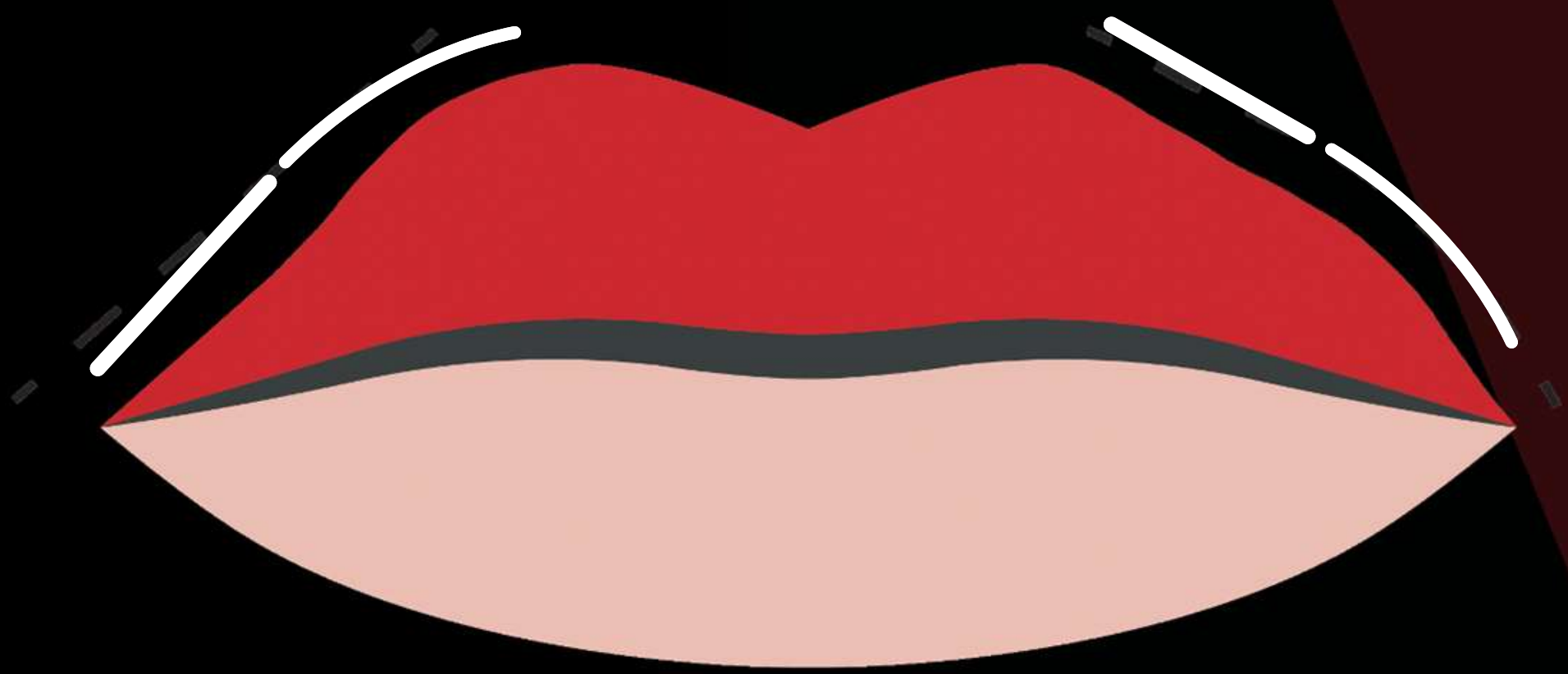

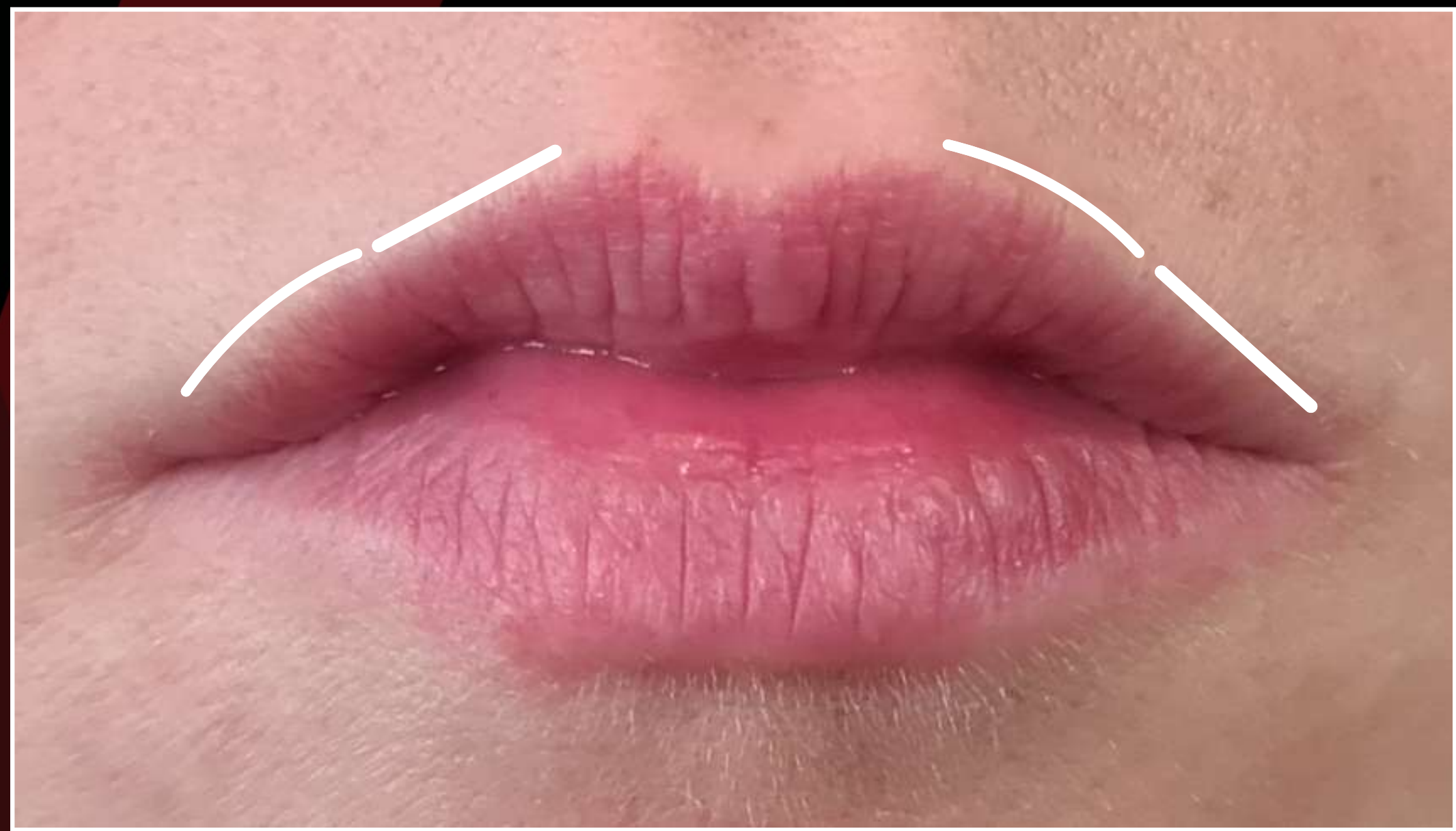

MIXED

Types

© Copyright of Andrea Felice Armenti and Soave Holding srl socio unico

KEYs CB CLIMB Border

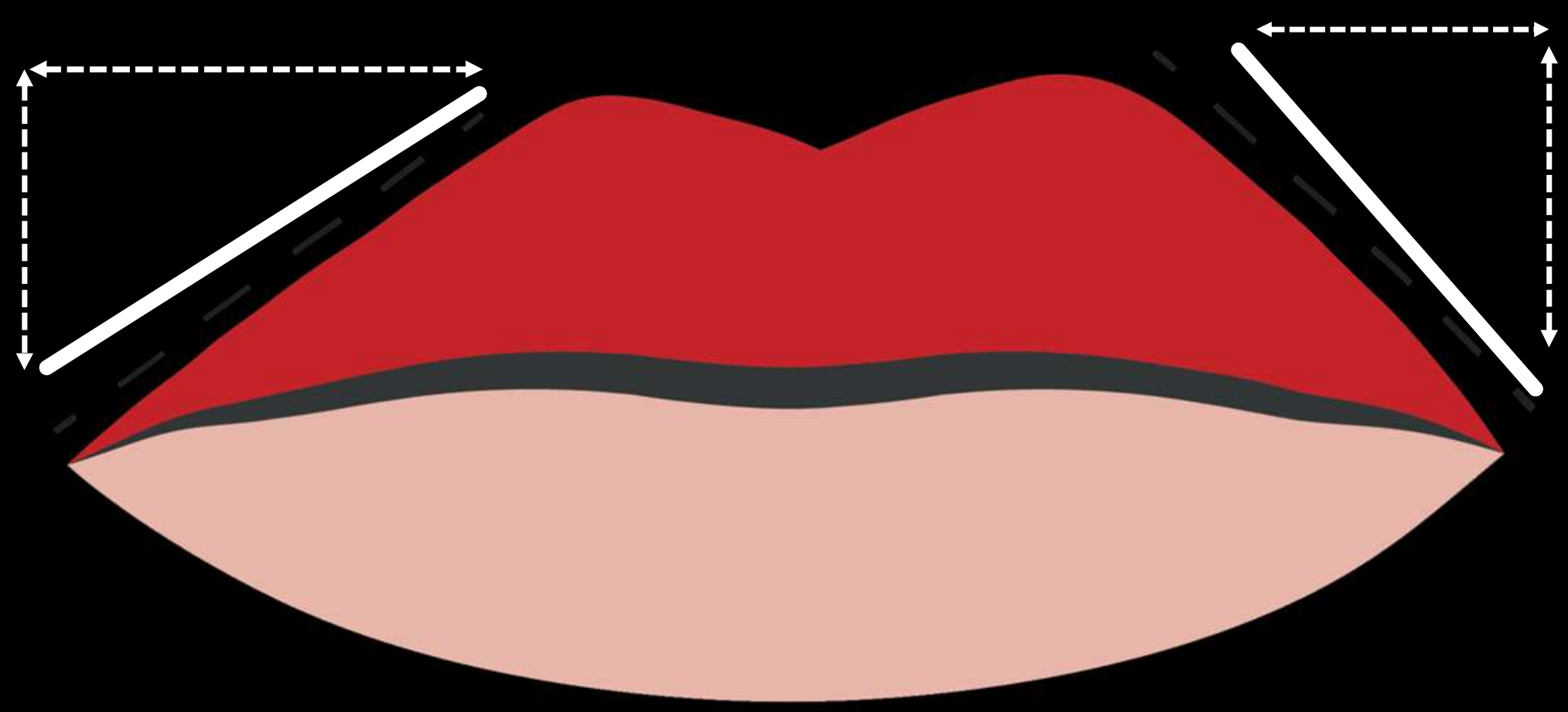

ASYMMETRIC

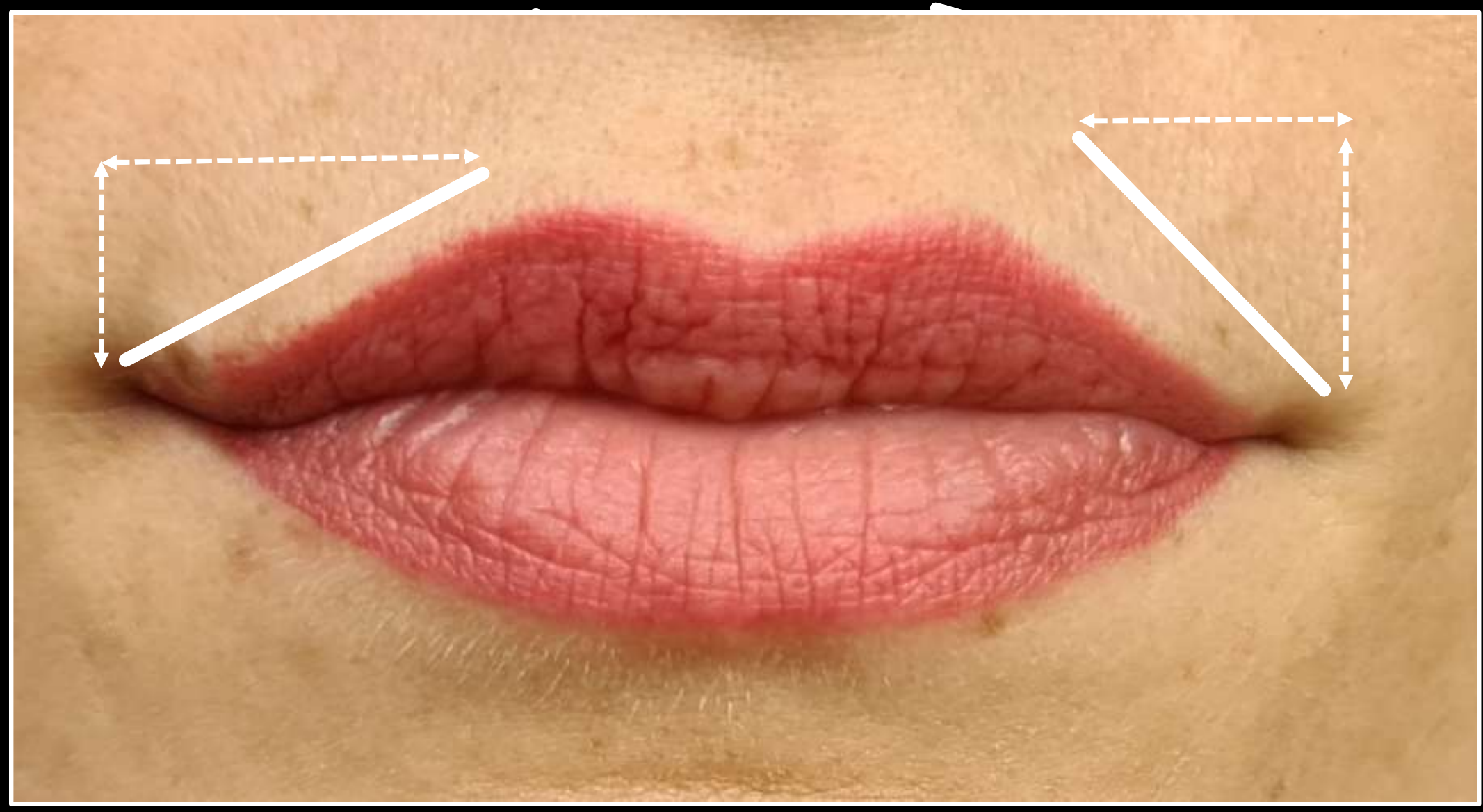

KEYs

© Copyright of Andrea Felice Armenti and Soave Holding srl socio unico

INDIVIDUAL ANALYSIS

KEYh CB CLIMB Border

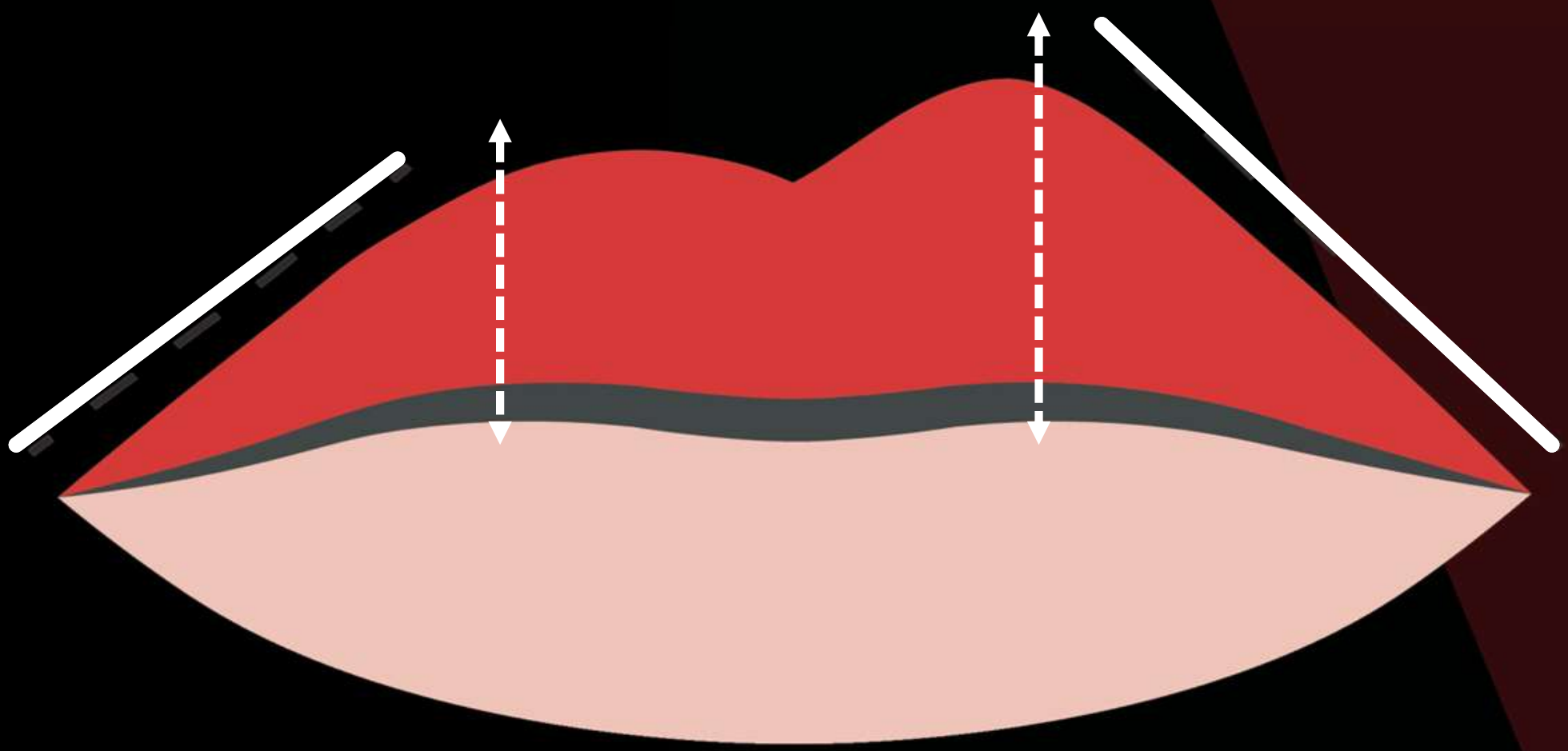

Different HEIGHT

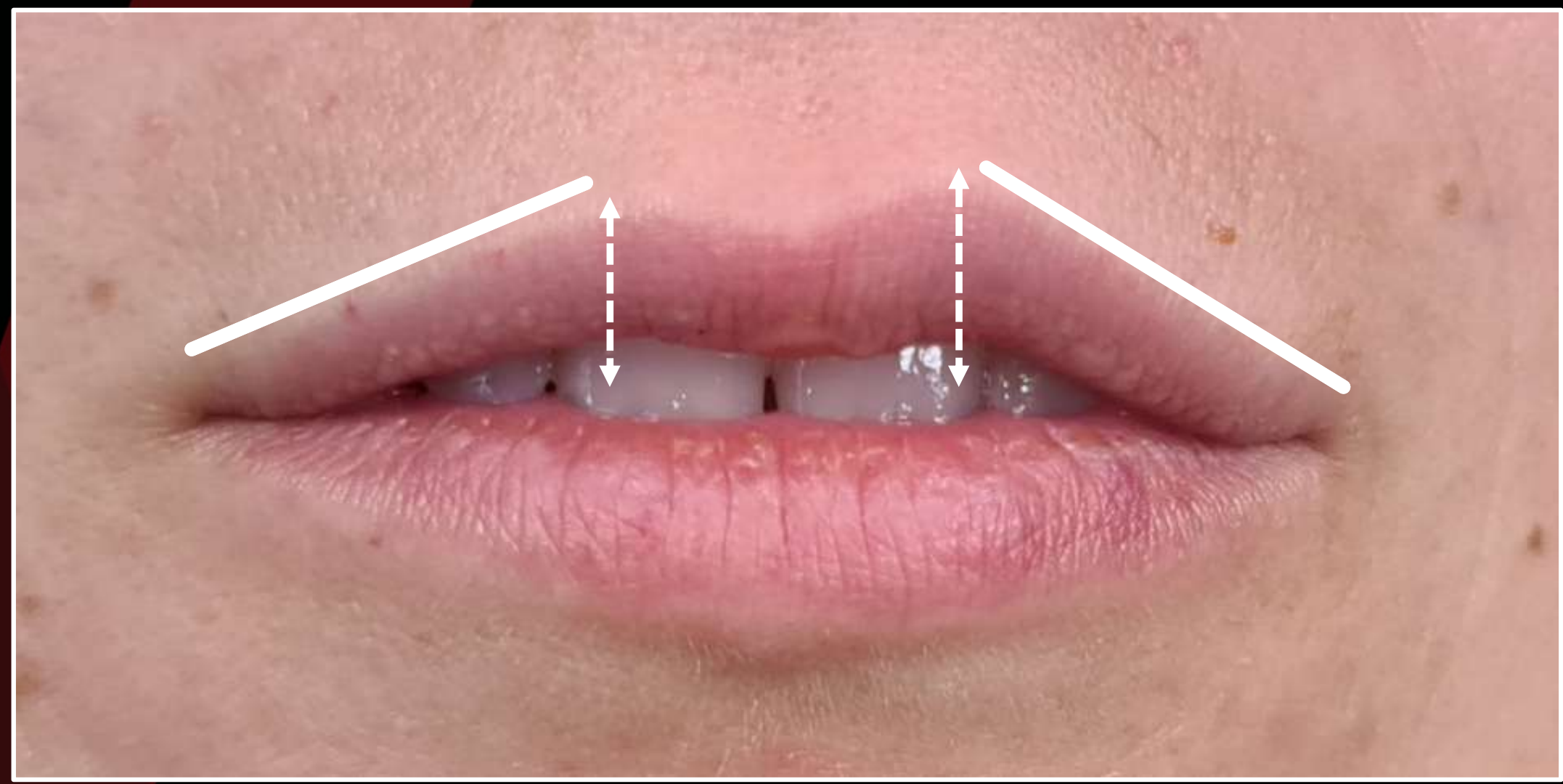

KEYh

© Copyright of Andrea Felice Armenti and Soave Holding srl socio unico

| LINES LVL

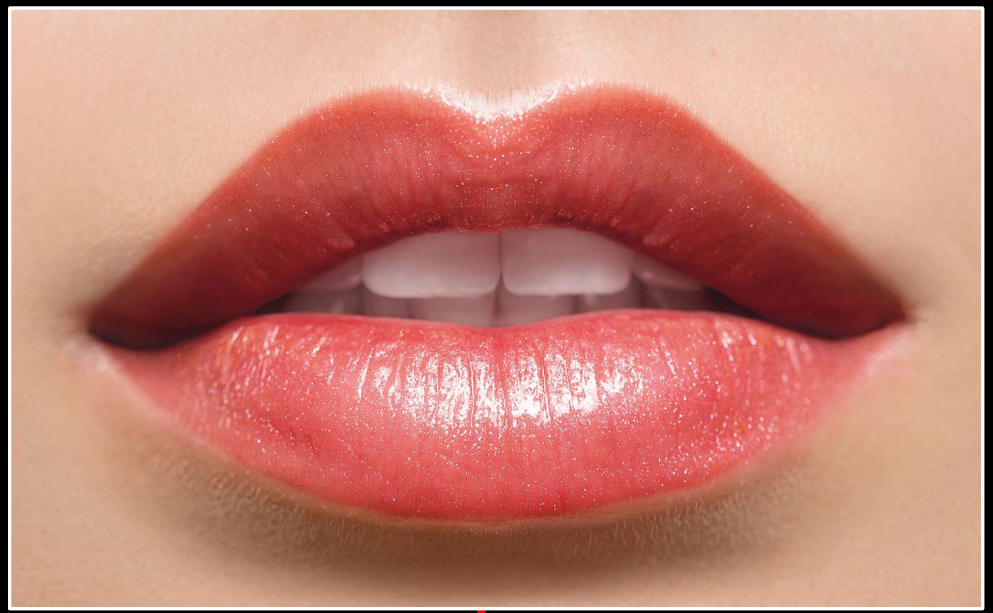

LIP ZONES

HB HILL Border

3 Types | 2 KEYs | 1 KEYh

© Copyright of Andrea Felice Armenti and Soave Holding srl socio unico

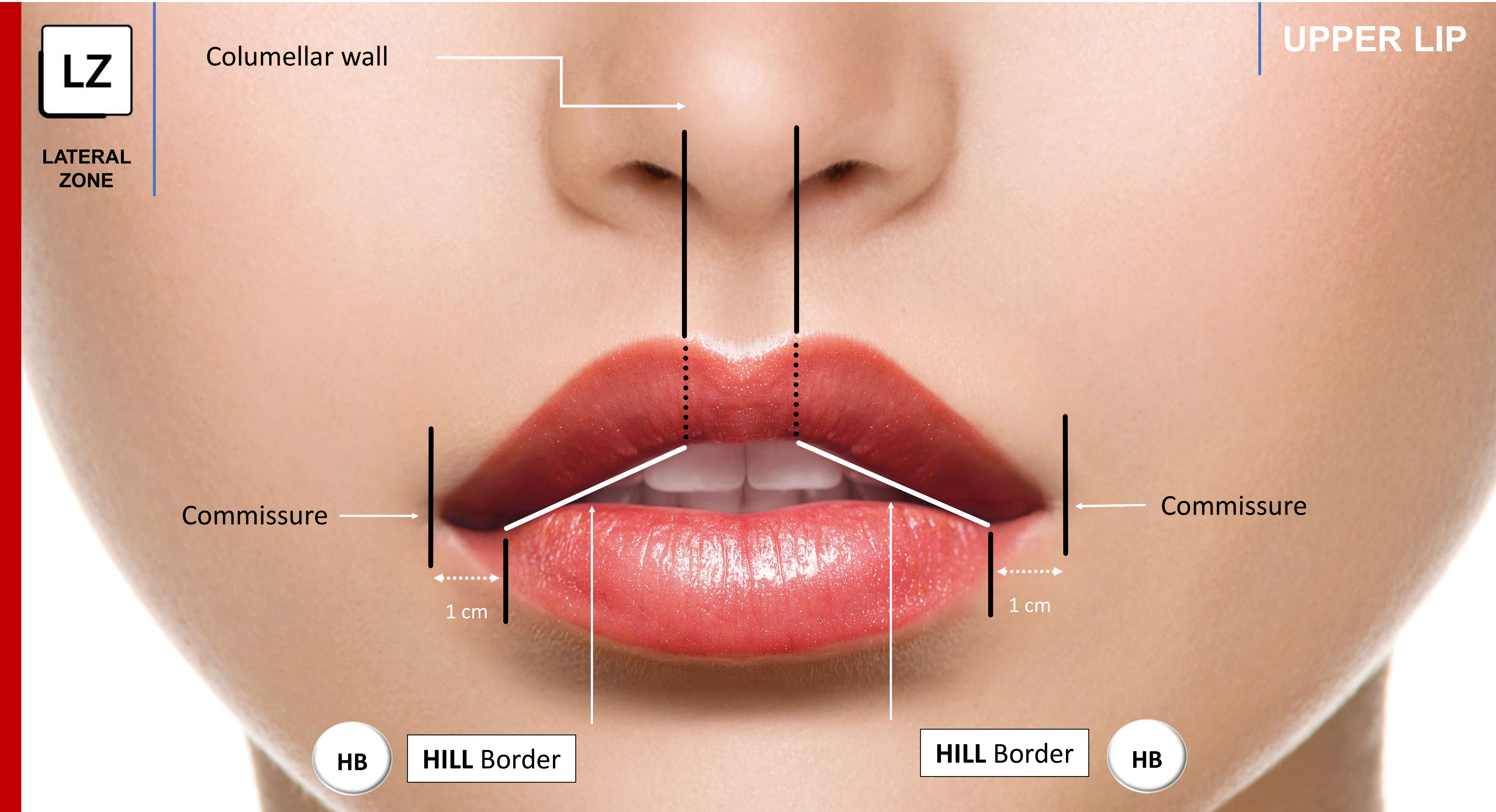

© Copyright of Andrea Felice Armenti and Soave Holding srl socio unico

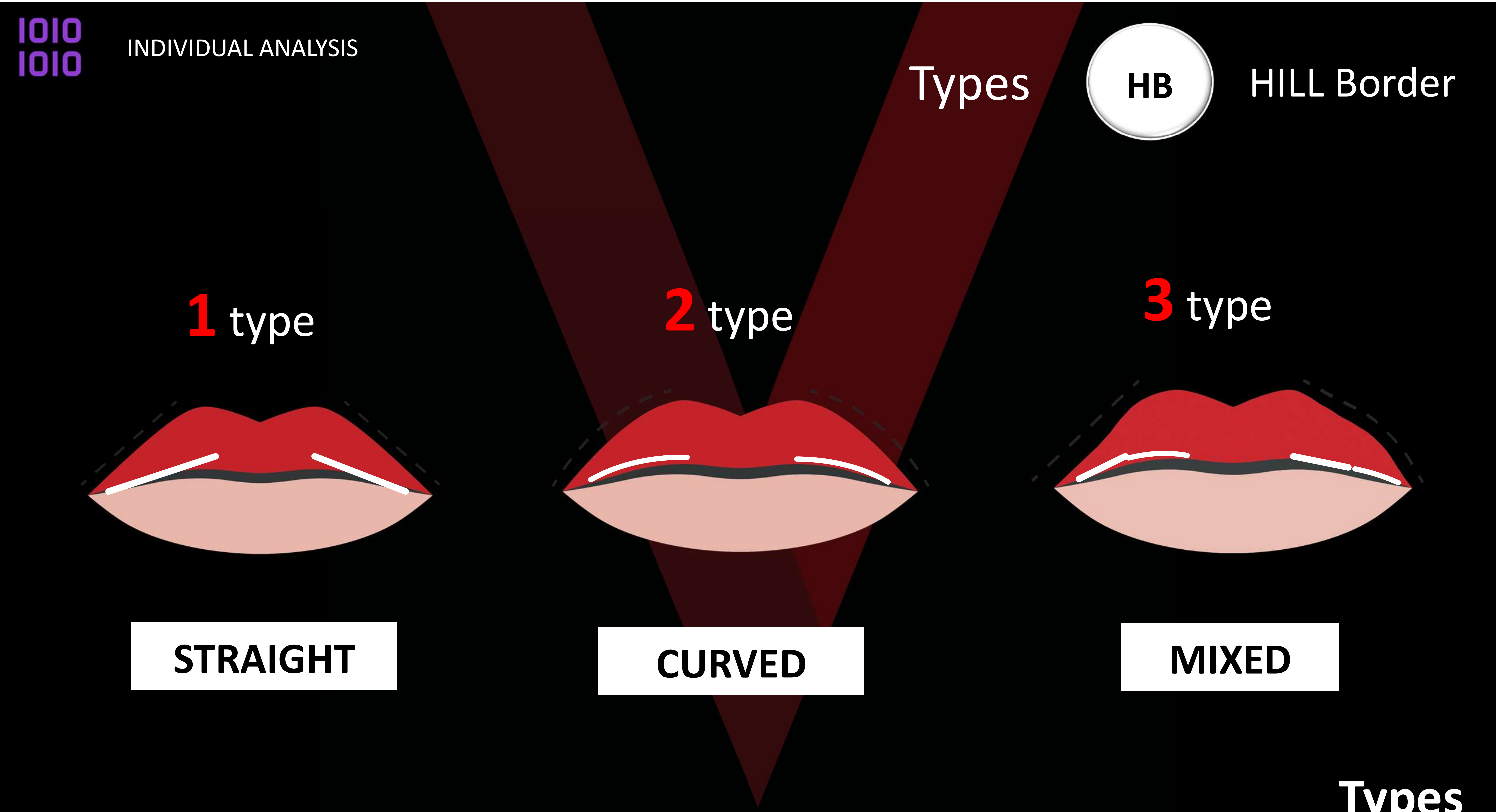

© Copyright of Andrea Felice Armenti and Soave Holding srl socio unico

INDIVIDUAL ANALYSIS

Types

HB

HILL Border

1 type

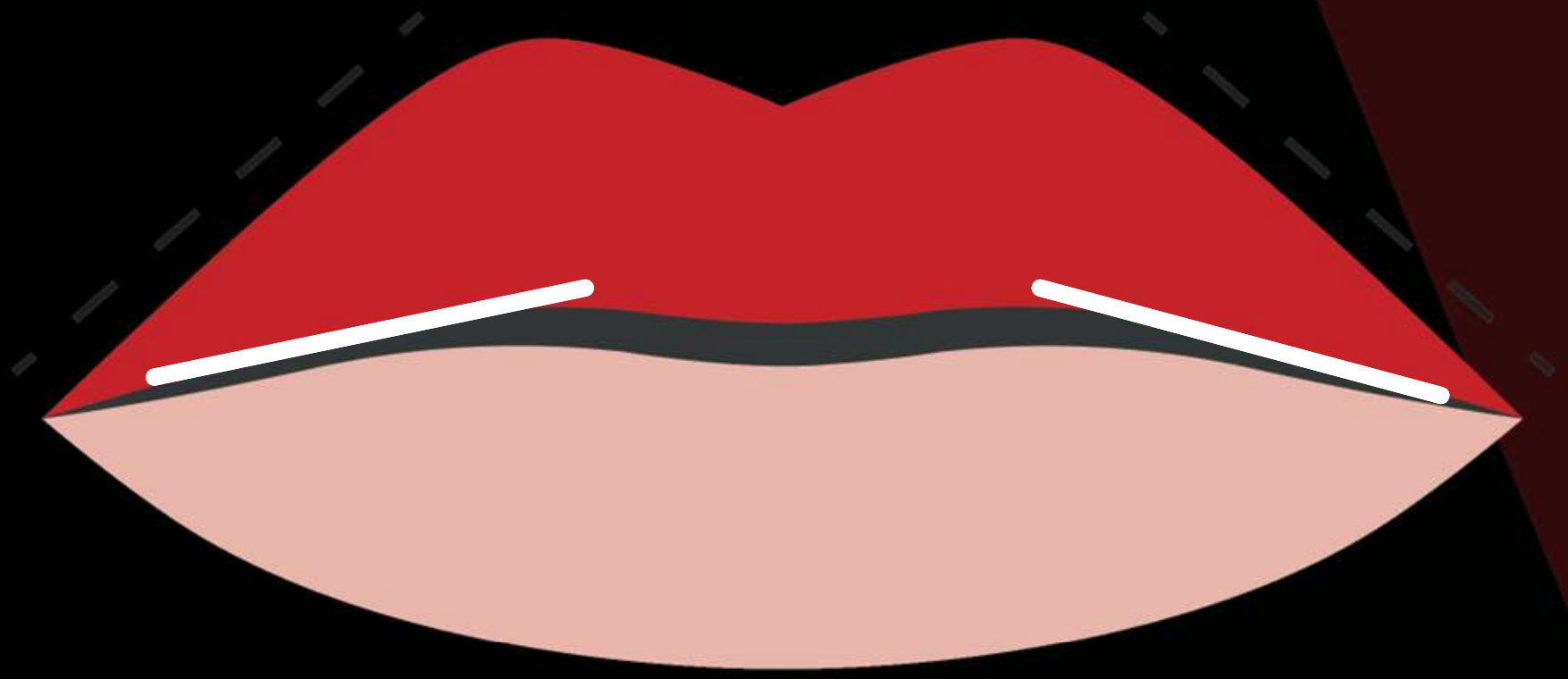

STRAIGHT

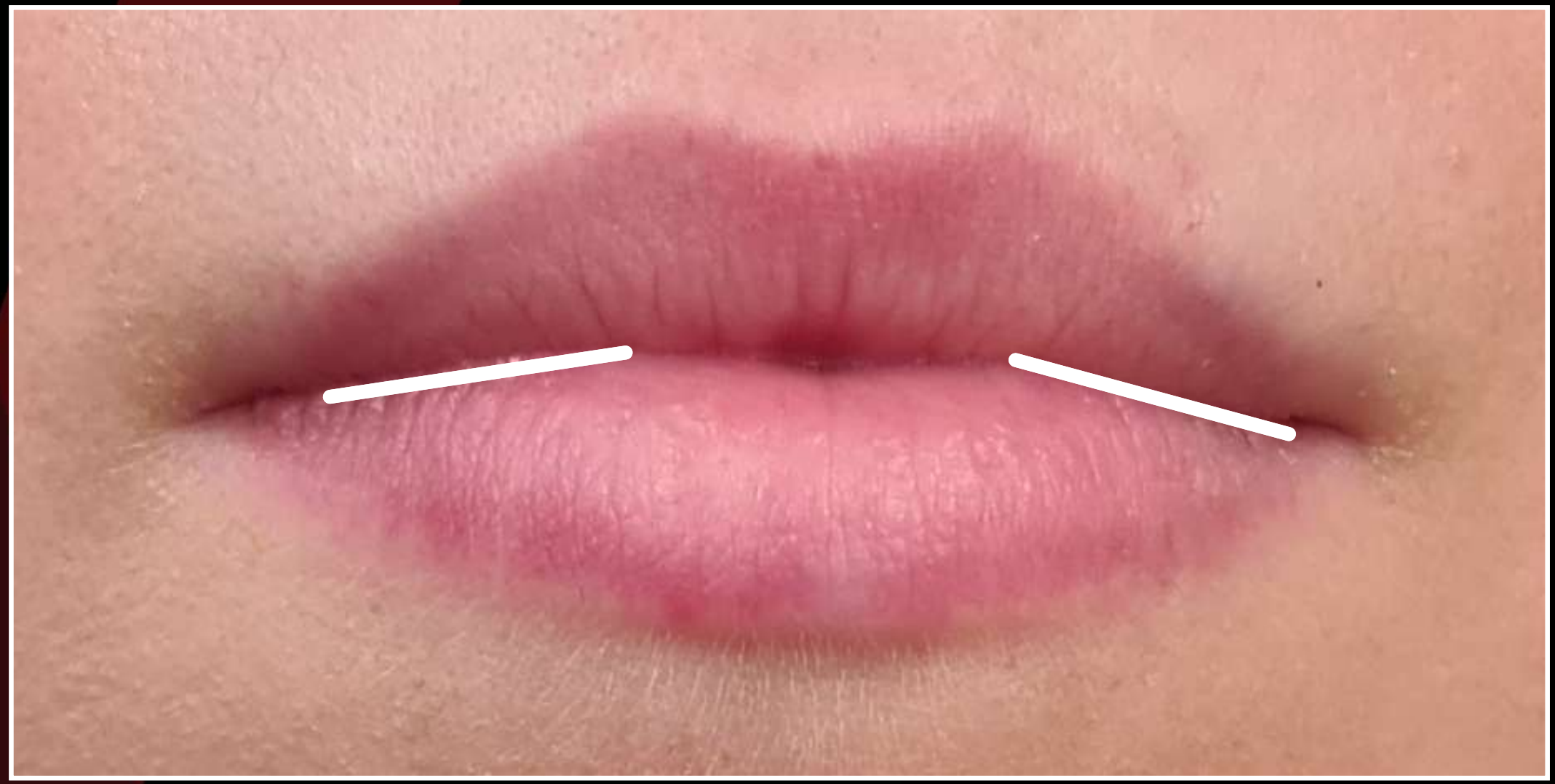

Types

© Copyright of Andrea Felice Armenti and Soave Holding srl socio unico

INDIVIDUAL ANALYSIS

Types HB HILL Border

2 type

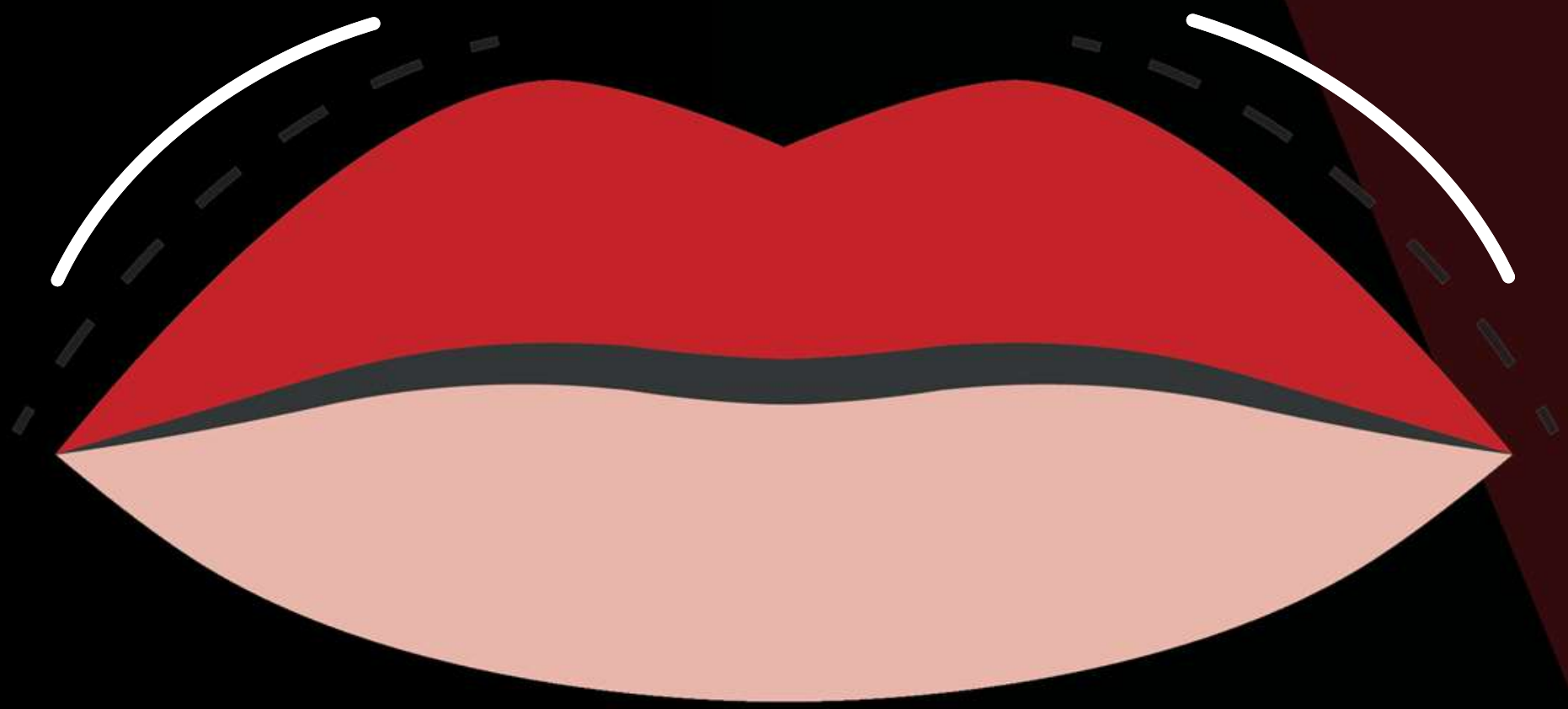

CURVED

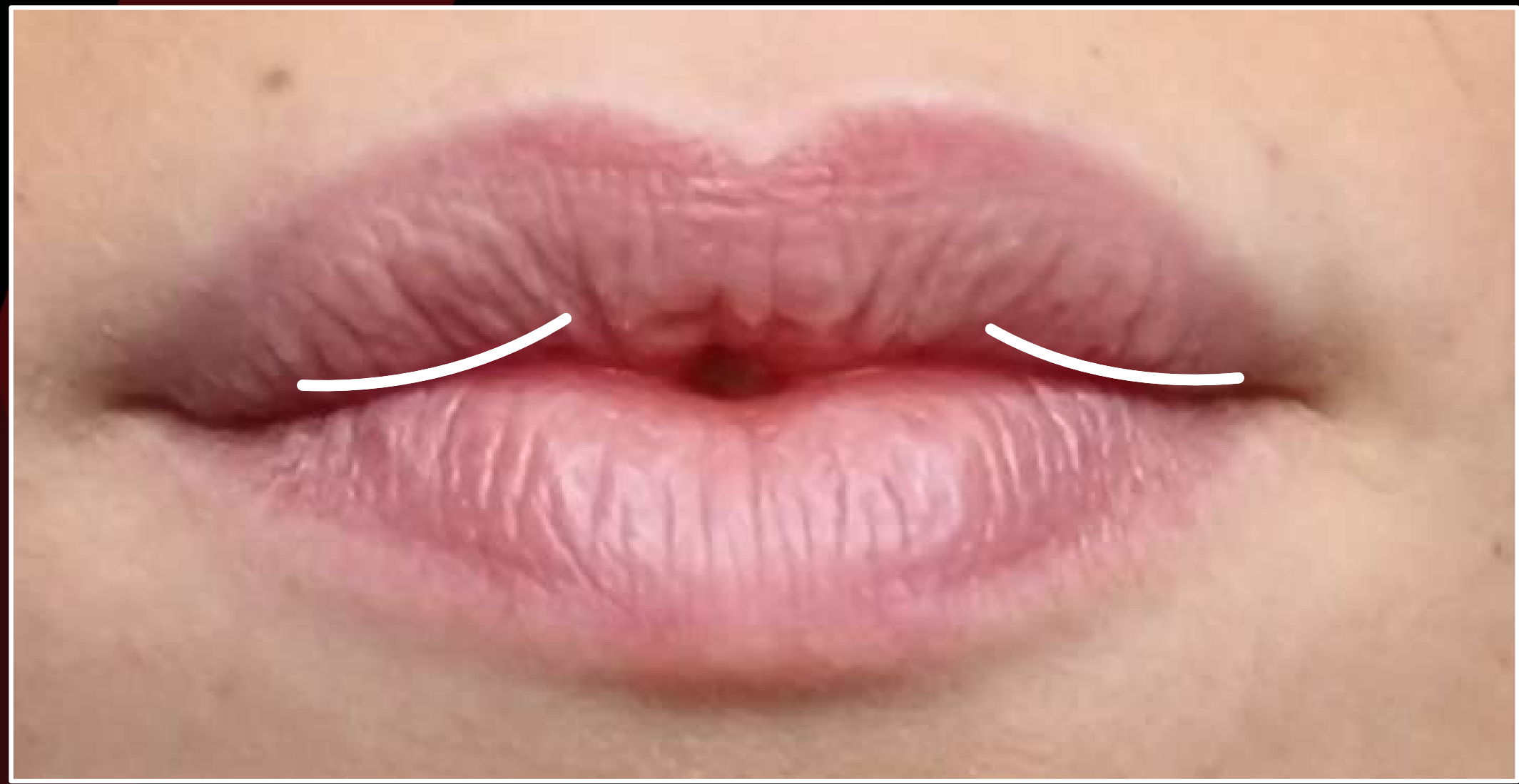

© Copyright of Andrea Felice Armenti and Soave Holding srl socio unico

Types

INDIVIDUAL ANALYSIS

Types

HB

HILL Border

3 type

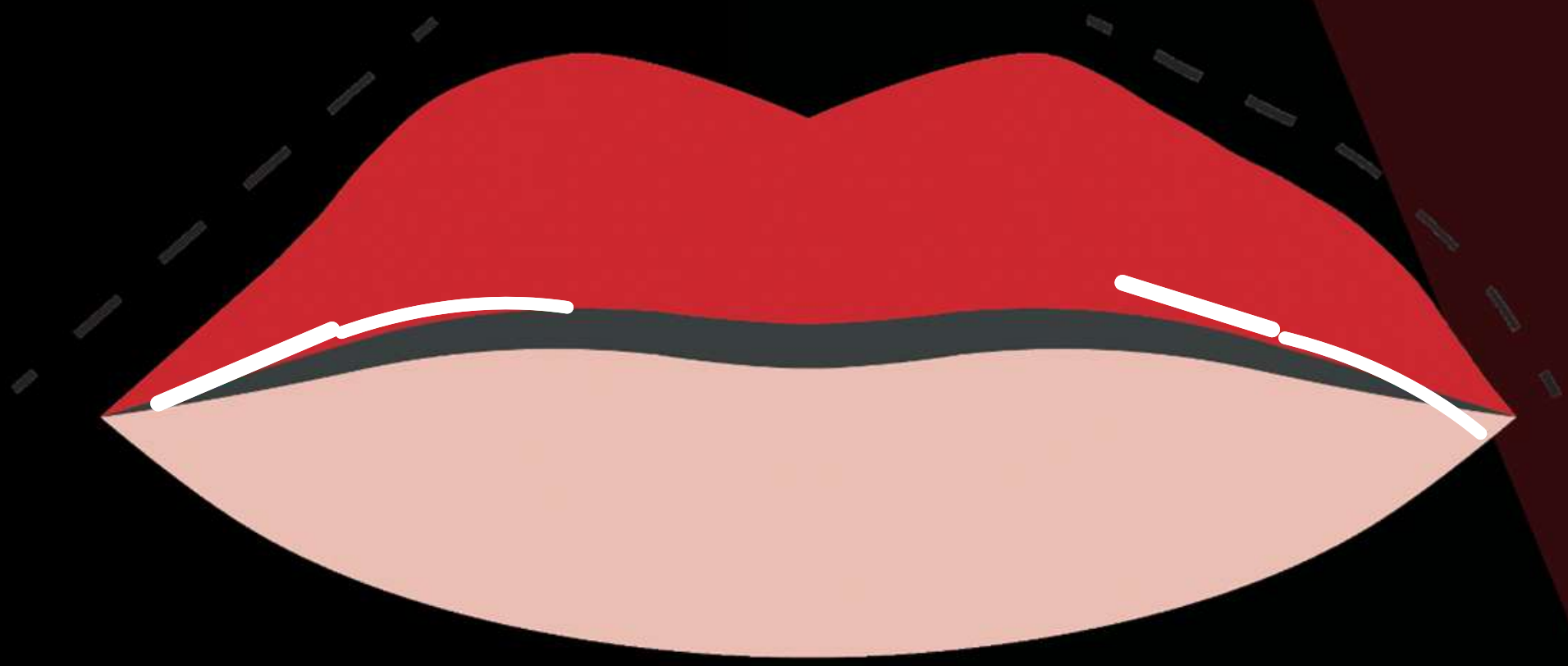

MIXED

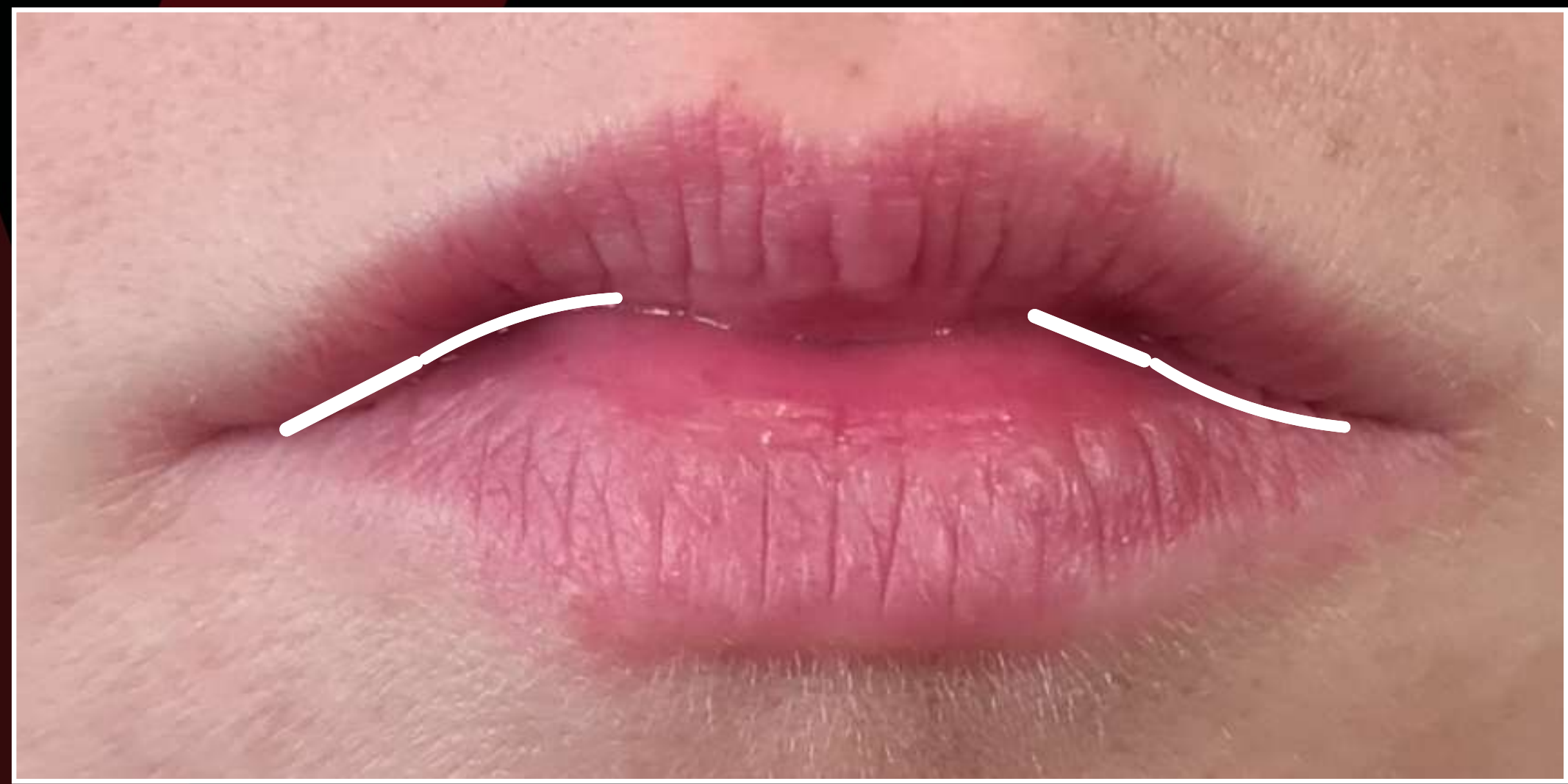

Types

© Copyright of Andrea Felice Armenti and Soave Holding srl socio unico

INDIVIDUAL ANALYSIS

KEYs

HB HILL Border

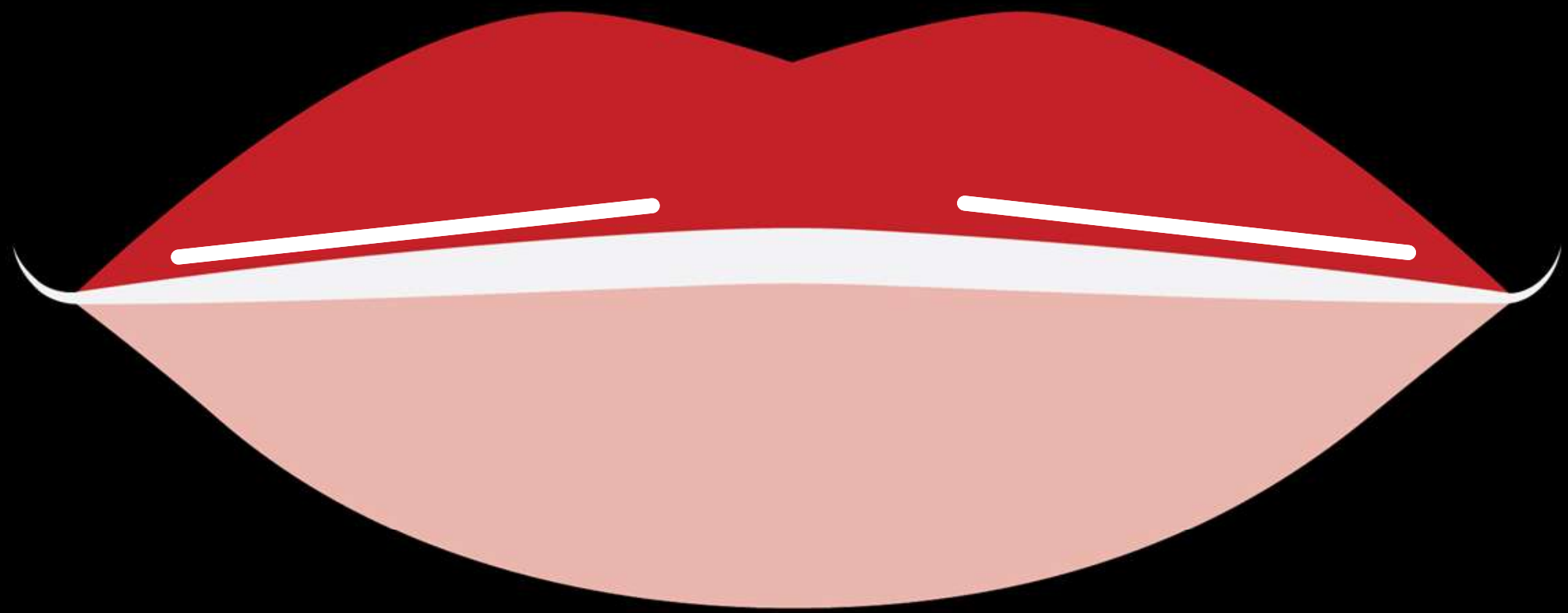

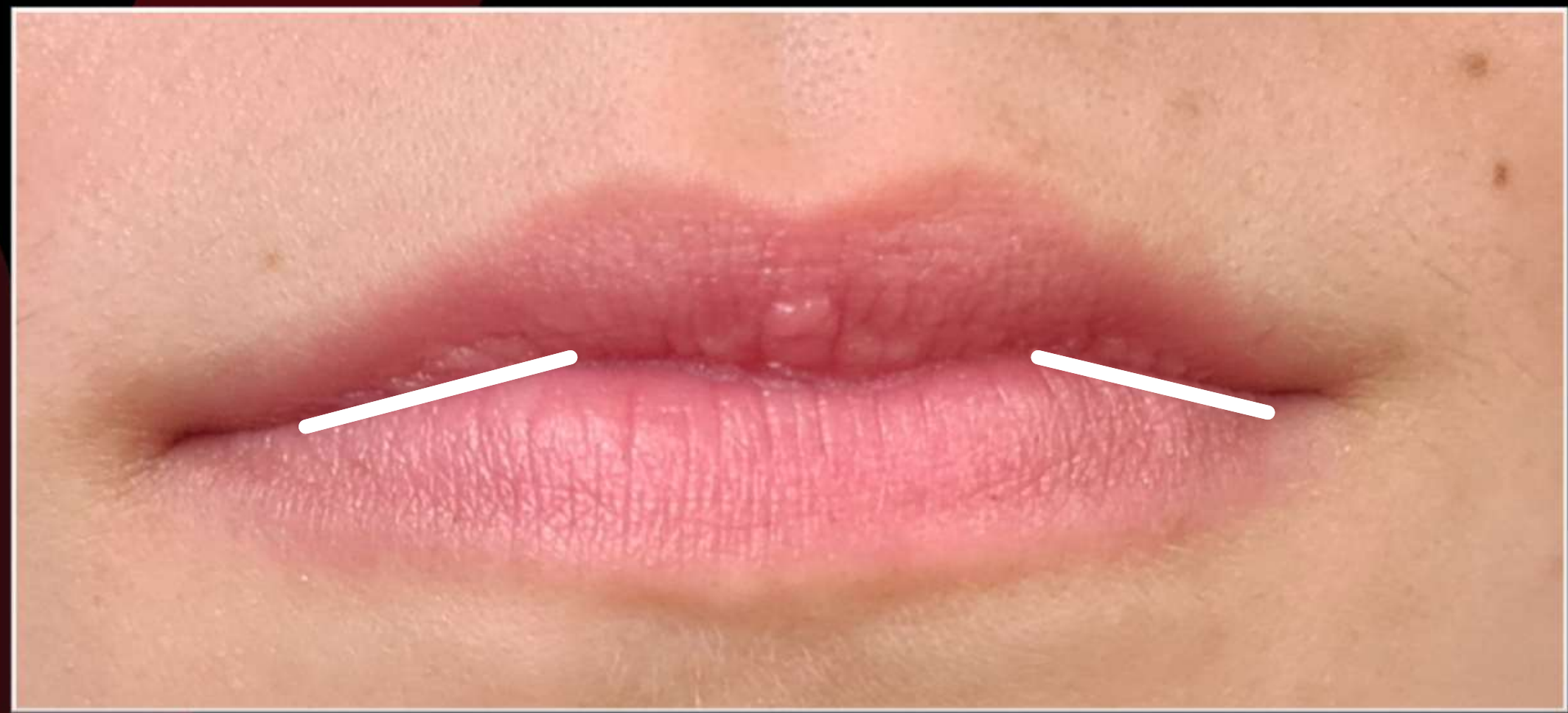

UPWARD

© Copyright of Andrea Felice Armenti and Soave Holding srl socio unico

KEYs

INDIVIDUAL ANALYSIS

KEYs

HB HILL Border

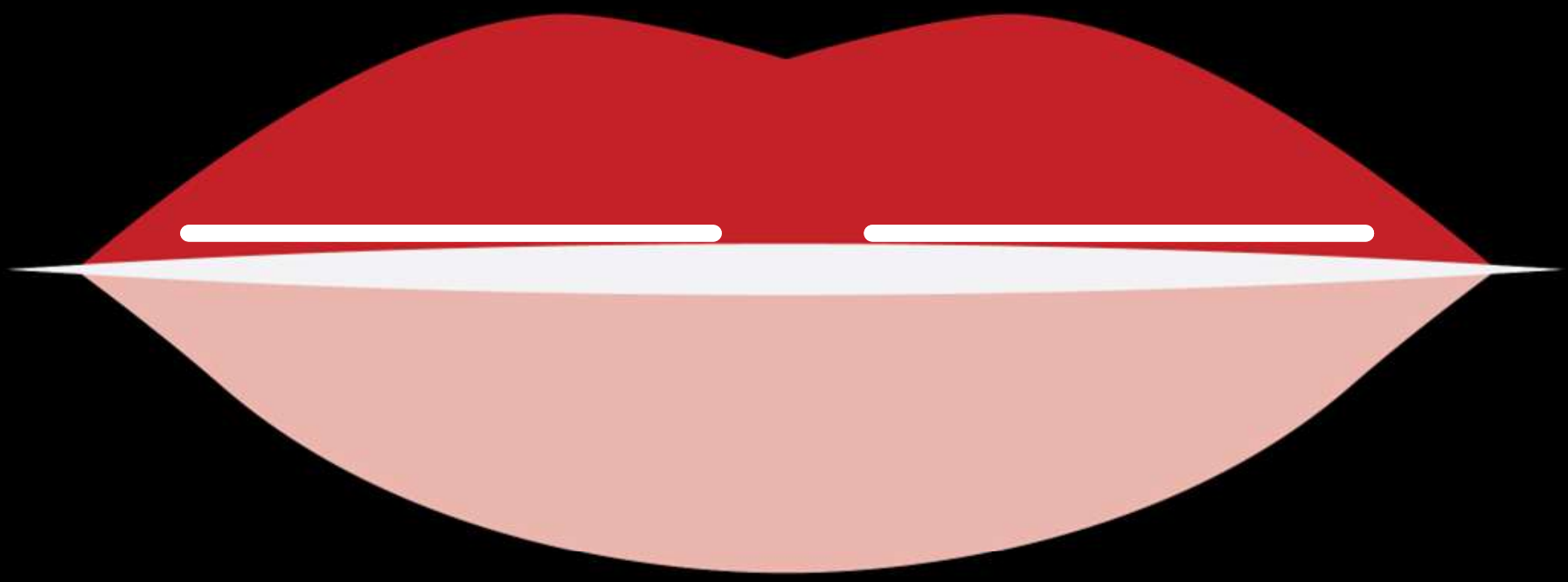

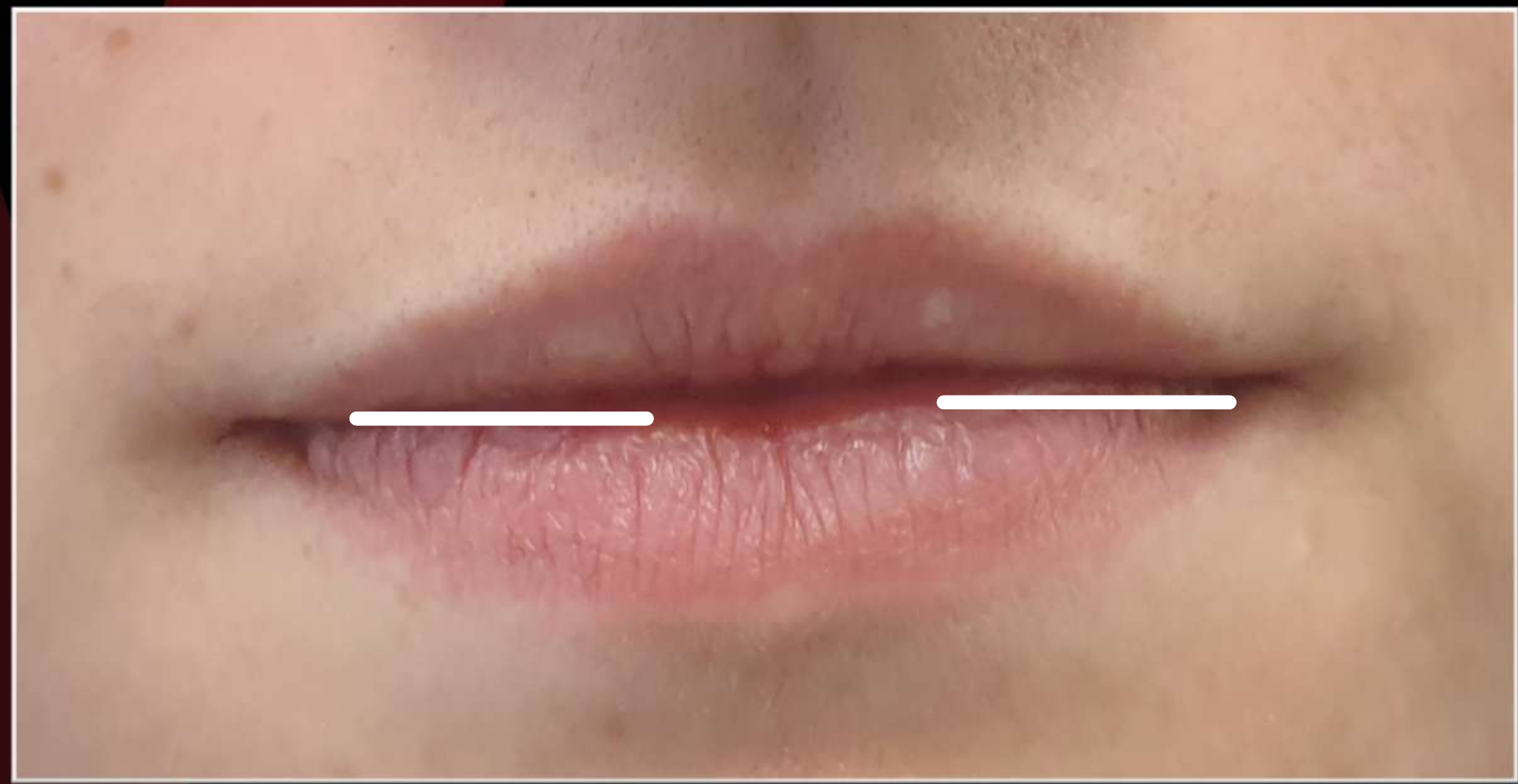

HORIZONTAL

© Copyright of Andrea Felice Armenti and Soave Holding srl socio unico

INDIVIDUAL ANALYSIS

KEYh

HB

HILL Border

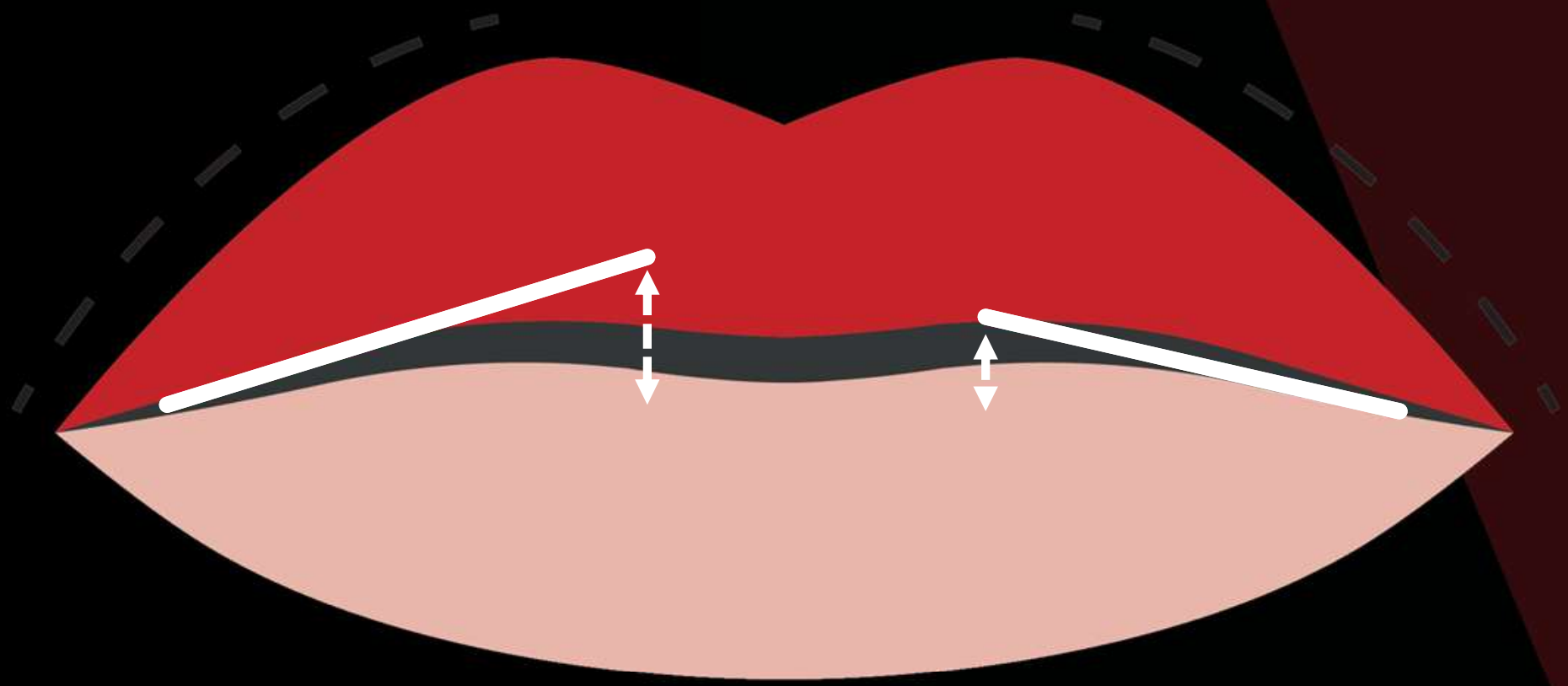

Different HEIGHT

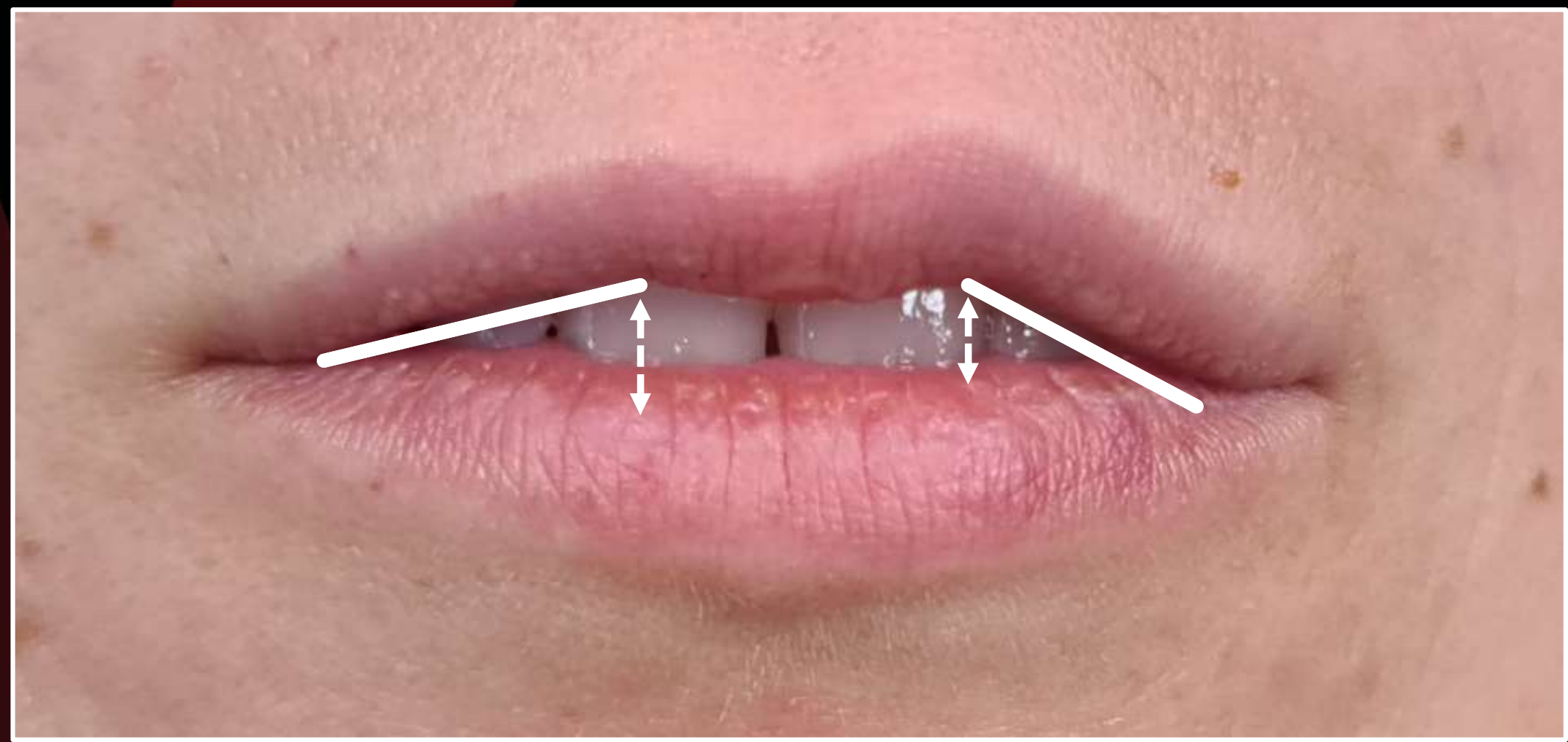

© Copyright of Andrea Felice Armenti and Soave Holding srl socio unico
