# Supplementary material for: LA VISION©: A Structured Approach to Lip Analysis and Aesthetic Treatment with Hyaluronic Acid Fillers
Source: Aesthetic Plast Surg. 2026 Jul 1;50(15):6316–44. doi: 10.1007/s00266-026-05998-7 (PMC13493426; doi:10.1007/s00266-026-05998-7)
Supplement: Supplementary file 3 [file 266_2026_5998_MOESM1_ESM.docx]

**Supplementary Table 1. Horizontal, vertical, and lateral proportions**

| **Class** | **Parameters** |
| --- | --- |
| Horizontal and vertical proportions | - Horizontal ratios (e.g. lip–nose width; lip width–mandibular angle; upper–lower lip) - Vertical ratios (e.g. superior–inferior prolabio) - Chin relations |
| Lateral profile and vectors | - Profile lines (e.g. Frankfurt Line, Ricketts Line, LipBeauty Line^a^) - Vermilion properties (superior vermilion vector; inferior vermilion curve type [central elliptical, full, inverted, flat]) |

*^a^*The LipBeauty Line is a schematic reference used to support structured analysis of upper–lower lip and chin relationships within the Individual Analysis module. It does not represent an aesthetic ideal or prescriptive target but rather a descriptive guide facilitating consistent morphological interpretation across patients (see Supplementary Material 1).
