# Supplementary material for: LA VISION©: A Structured Approach to Lip Analysis and Aesthetic Treatment with Hyaluronic Acid Fillers
Source: Aesthetic Plast Surg. 2026 Jul 1;50(15):6316–44. doi: 10.1007/s00266-026-05998-7 (PMC13493426; doi:10.1007/s00266-026-05998-7)
Supplement: Supplementary file 4 [file 266_2026_5998_MOESM2_ESM.docx]

**Supplementary Table 2. Indication prevalence (multi-label)**

| **Indication** | **Patients, n (%)** |
| --- | --- |
| Volume | 64 (16.0) |
| Restoration | 214 (53.5) |
| Asymmetry | 173 (43.2) |
| Hydration | 50 (12.5) |
| Smile | 63 (15.8) |
| Mixed cases (≥ 2 indications) | 132 (33.0) |
| **Pairwise co-occurrence** | **Patients, n (%)** |
| Volume + Restoration | 29 (7.2) |
| Volume + Asymmetry | 24 (6.0) |
| Volume + Hydration | 5 (1.2) |
| Volume + Smile | 7 (1.8) |
| Restoration + Asymmetry | 58 (14.5) |
| Restoration + Hydration | 15 (3.8) |
| Restoration + Smile | 20 (5.0) |
| Asymmetry + Hydration | 5 (1.2) |
| Asymmetry + Smile | 22 (5.5) |
| Hydration + Smile | 11 (2.8) |
