# Supplementary material for: LA VISION©: A Structured Approach to Lip Analysis and Aesthetic Treatment with Hyaluronic Acid Fillers
Source: Aesthetic Plast Surg. 2026 Jul 1;50(15):6316–44. doi: 10.1007/s00266-026-05998-7 (PMC13493426; doi:10.1007/s00266-026-05998-7)
Supplement: Supplementary file 5 [file 266_2026_5998_MOESM3_ESM.docx]

**Supplementary Table 3. Single vs mixed indications**

| **Treatment satisfaction, mean ± SD** | | | | |
| --- | --- | --- | --- | --- |
| **Group** | **T6M** | | **T1Y** | |
| Single indication (N=268) | 8.10 ± 0.69 | | 7.55 ± 0.70 | |
| Mixed indications (N=132) | 8.07 ± 0.65 | | 7.52 ± 0.67 | |
| **AEs per treatment session (all timepoints pooled), n (%) [Wilson 95% CI]** | | | | |
| **Group** | **Edema** | **Bruising** | **Late-onset swelling** | **Nodule formation** |
| Single indication (N=697) | 104 (14.9)  [12.5–17.8] | 68 (9.8)  [7.8–12.2] | 2 (0.3)  [0.1–1.0] | 1 (0.1)  [0.0–0.8] |
| Mixed indications (N=342) | 50 (14.6)  [11.3–18.8] | 34 (9.9)  [7.2–13.6] | 2 (0.6)  [0.2–2.1] | 0 (0.0)  [0.0–1.1] |

‘Mixed’ denotes patients with ≥ 2 indications at baseline; ‘Single’ denotes exactly 1 indication. AE, adverse event; CI, confidence interval; T1Y, 1-year treatment; T6M, 6-month treatment.
