# Supplementary material for: LA VISION©: A Structured Approach to Lip Analysis and Aesthetic Treatment with Hyaluronic Acid Fillers
Source: Aesthetic Plast Surg. 2026 Jul 1;50(15):6316–44. doi: 10.1007/s00266-026-05998-7 (PMC13493426; doi:10.1007/s00266-026-05998-7)
Supplement: Supplementary file 6 [file 266_2026_5998_MOESM4_ESM.docx]

**Supplementary Table 4.** **Per-product adverse event rates and treatment satisfaction.**

| **Product** | **Adverse events, n (%) [95% CI]** | | | | | **Treatment satisfaction, mean ± SD [95% CI]** | |
| --- | --- | --- | --- | --- | --- | --- | --- |
|  | **Sessions (N)** | **Edema** | **Bruising** | **Late-onset swelling** | **Nodule formation** | **N** | **Score, mean ± SD** |
| Flux | 72 | 0 (0)  [0.0–5.1] | 7 (9.7)  [4.8–18.7] | 0 (0)  [0.0–5.1] | 0 (0)  [0.0–5.1] | 36 | 8.14 ± 0.56 [7.96–8.32] |
| Intense Rheology | 176 | 0 (0)  [0.0–2.1] | 11 (6.3)  [3.5–10.8] | 0 (0)  [0.0–2.1] | 0 (0)  [0.0–2.1] | 117 | 7.75 ± 0.64 [7.63–7.87] |
| Juvéderm Ultra 3 | 218 | 0 (0)  [0.0–1.7] | 21 (9.6)  [6.4–14.3] | 0 (0)  [0.0–1.7] | 0 (0)  [0.0–1.7] | 145 | 7.75 ± 0.69 [7.63–7.86] |
| Juvéderm Volbella | 20 | 10 (50.0)  [29.9–70.1] | 0 (0)  [0.0–16.1] | 0 (0)  [0.0–16.1] | 0 (0)  [0.0–16.1] | 10 | 8.00 ± 0.58 [7.64–8.36] |
| Juvéderm Volift | 93 | 42 (45.2)  [35.4–55.3] | 13 (14.0)  [8.4–22.5] | 2 (2.2)  [0.6–7.5] | 1 (1.1)  [0.2–5.8] | 47 | 8.05 ± 0.60 [7.88–8.22] |
| RHA II | 10 | 1 (10.0)  [1.8–40.4] | 0 (0)  [0.0–27.8] | 0 (0)  [0.0–27.8] | 0 (0)  [0.0–27.8] | 5 | 8.00 ± 0.63 [7.45–8.55] |
| RHA III | 137 | 4 (2.9)  [1.1–7.3] | 9 (6.6)  [3.5–12.0] | 0 (0)  [0.0–2.7] | 0 (0)  [0.0–2.7] | 70 | 8.07 ± 0.61 [7.93–8.22] |
| Stylage Lips Plus | 135 | 14 (10.4)  [6.3–16.7] | 18 (13.3)  [8.6–20.1] | 0 (0)  [0.0–2.8] | 0 (0)  [0.0–2.8] | 90 | 7.69 ± 0.67 [7.56–7.83] |
| Stylage Special Lips | 177 | 67 (37.9)  [31.0–45.2] | 14 (7.9)  [4.8–12.8] | 2 (1.1)  [0.3–4.0] | 0 (0)  [0.0–2.1] | 118 | 7.78 ± 0.66 [7.66–7.90] |

CI, confidence interval; SD, standard deviation.
