# Supplementary material for: LA VISION©: A Structured Approach to Lip Analysis and Aesthetic Treatment with Hyaluronic Acid Fillers
Source: Aesthetic Plast Surg. 2026 Jul 1;50(15):6316–44. doi: 10.1007/s00266-026-05998-7 (PMC13493426; doi:10.1007/s00266-026-05998-7)
Supplement: Supplementary file 7 [file 266_2026_5998_MOESM5_ESM.docx]

**Supplementary Table 5. Adverse events per treatment session by primary indication and timepoint**

| **Primary** | **T0** | **T6M** | **T1Y** |
| --- | --- | --- | --- |
| **Edema (non-late)** | | | |
| Asymmetry | 16 / 144 (11.11) [6.96–17.29] | 16 / 144 (11.11) [6.96–17.29] | 9 / 81 (11.11)  [5.96–19.79] |
| Hydration | 7 / 38 (18.42)  [9.22–33.42] | 6 / 38 (15.79)  [7.44–30.42] | 1 / 20 (5.00)  [0.89–23.61] |
| Restoration | 37 / 166 (22.29) [16.63–29.21] | 22 / 166 (13.25) [8.92–19.25] | 8 / 109 (7.34)  [3.77–13.82] |
| Smile | 7 / 33 (21.21)  [10.68–37.75] | 3 / 33 (9.09)  [3.14–23.57] | 1 / 19 (5.26)  [0.94–24.64] |
| Volume | 2 / 19 (10.53)  [2.94–31.39] | 1 / 19 (5.26)  [0.94–24.64] | 0 / 10 (0.00)  [0.00–27.75] |
| **Bruising** | | | |
| Asymmetry | 12 / 144 (8.33)  [4.83–14.00] | 12 / 144 (8.33)  [4.83–14.00] | 3 / 81 (3.70)  [1.27–10.33] |
| Hydration | 5 / 38 (13.16)  [5.75–27.33] | 7 / 38 (18.42)  [9.22–33.42] | 2 / 20 (10.00)  [2.79–30.10] |
| Restoration | 22 / 166 (13.25) [8.92–19.25] | 18 / 166 (10.84) [6.97–16.49] | 6 / 109 (5.50)  [2.55–11.49] |
| Smile | 3 / 33 (9.09)  [3.14–23.57] | 1 / 33 (3.03)  [0.54–15.32] | 1 / 19 (5.26)  [0.94–24.64] |
| Volume | 0 / 19 (0)  [0.00–16.82] | 0 / 19 (0)  [0.00–16.82] | 1 / 10 (10.00)  [1.79–40.42] |
| **Late-onset swelling** | | | |
| Asymmetry | 0 / 144 (0)  [0.00–2.60] | 2 / 144 (1.39)  [0.38–4.92] | 0 / 81 (0)  [0.00–4.53] |
| Hydration | 1 / 38 (2.63)  [0.47–13.50] | 0 / 38 (0)  [0.00–9.18] | 0 / 20 (0)  [0.00–16.11] |
| Restoration | 1 / 166 (0.60)  [0.11–3.33] | 0 / 166 (0)  [0.00–2.26] | 0 / 109 (0)  [0.00–3.40] |
| Smile | 0 / 33 (0)  [0.00–10.43] | 0 / 33 (0)  [0.00–10.43] | 0 / 19 (0)  [0.00–16.82] |
| Volume | 0 / 19 (0)  [0.00–16.82] | 0 / 19 (0)  [0.00–16.82] | 0 / 10 (0)  [0.00–27.75] |
| **Nodule formation** | | | |
| Asymmetry | 0 / 144 (0)  [0.00–2.60] | 0 / 144 (0)  [0.00–2.60] | 0 / 81 (0)  [0.00–4.53] |
| Hydration | 0 / 38 (0)  [0.00–9.18] | 0 / 38 (0)  [0.00–9.18] | 0 / 20 (0)  [0.00–16.11] |
| Restoration | 0 / 166 (0)  [0.00–2.26] | 1 / 166 (0.60)  [0.11–3.33] | 0 / 109 (0)  [0.00–3.40] |
| Smile | 0 / 33 (0)  [0.00–10.43] | 0 / 33 (0)  [0.00–10.43] | 0 / 19 (0)  [0.00–16.82] |
| Volume | 0 / 19 (0)  [0.00–16.82] | 0 / 19 (0)  [0.00–16.82] | 0 / 10 (0)  [0.00–27.75] |
| **Swelling composite (edema including late onset)** | | | |
| Asymmetry | 16 / 144 (11.11)  [6.96–17.29] | 18 / 144 (12.50)  [8.06–18.89] | 9 / 81 (11.11)  [5.96–19.79] |
| Hydration | 8 / 38 (21.05)  [11.07–36.35] | 6 / 38 (15.79)  [7.44–30.42] | 1 / 20 (5.00)  [0.89–23.61] |
| Restoration | 38 / 166 (22.89)  [17.16–29.85] | 22 / 166 (13.25)  [8.92–19.25] | 8 / 109 (7.34)  [3.77–13.82] |
| Smile | 7 / 33 (21.21)  [10.68–37.75] | 3 / 33 (9.09)  [3.14–23.57] | 1 / 19 (5.26)  [0.94–24.64] |
| Volume | 2 / 19 (10.53)  [2.94–31.39] | 1 / 19 (5.26)  [0.94–24.64] | 0 / 10 (0)  [0.00–27.75] |

All data are n/N (%) [Wilson 95% confidence interval]. Edema (non-late) excludes episodes labeled late / delayed / tardive. Late-onset swelling is listed separately. Primary indication was assigned at T0 and not re-assigned at maintenance. Analyses reflect maintenance of the primary goal. T0, baseline treatment; T1Y, 1-year treatment; T6M, 6-month treatment.
