# Supplementary material for: LA VISION©: A Structured Approach to Lip Analysis and Aesthetic Treatment with Hyaluronic Acid Fillers
Source: Aesthetic Plast Surg. 2026 Jul 1;50(15):6316–44. doi: 10.1007/s00266-026-05998-7 (PMC13493426; doi:10.1007/s00266-026-05998-7)
Supplement: Supplementary file 8 [file 266_2026_5998_MOESM6_ESM.docx]

**Supplementary Table 6. Adverse events per treatment session by device and timepoint**

| **Device** | **T0** | **T6M** | **T1Y** |
| --- | --- | --- | --- |
| **Edema (non-late)** | | | |
| Cannula | 12 / 56 (21.43)  [12.71–33.82] | 3 / 56 (5.36)  [1.84–14.61] | 5 / 36 (13.89)  [6.08–28.66] |
| Needle | 57 / 344 (16.57) [13.01–20.86] | 45 / 344 (13.08) [9.92–17.06] | 14 / 203 (6.90)  [4.15–11.24] |
| **Bruising** | | | |
| Cannula | 7 / 56 (12.50)  [6.19–23.63] | 6 / 56 (10.71)  [5.00–21.47] | 0 / 36 (0)  [0.00–9.64] |
| Needle | 35 / 344 (10.17)  [7.41–13.82] | 32 / 344 (9.30)  [6.67–12.84] | 13 / 203 (6.40)  [3.78–10.65] |
| **Late-onset swelling** | | | |
| Cannula | 2 / 56 (3.57)  [0.98–12.12] | 0 / 56 (0)  [0.00–6.42] | 0 / 36 (0)  [0.00–9.64] |
| Needle | 0 / 344 (0)  [0.00–1.10] | 2 / 344 (0.58)  [0.16–2.09] | 0 / 203 (0)  [0.00–1.86] |
| **Nodule formation** | | | |
| Cannula | 0 / 56 (0)  [0.00–6.42] | 1 / 56 (1.79)  [0.32–9.45] | 0 / 36 (0)  [0.00–9.64] |
| Needle | 0 / 344 (0)  [0.00–1.10] | 0 / 344 (0)  [0.00–1.10] | 0 / 203 (0)  [0.00–1.86] |
| **Swelling composite (edema including late onset)** | | | |
| Cannula | 14 / 56 (25.00)  [15.52–37.69] | 3 / 56 (5.36)  [1.84–14.61] | 5 / 36 (13.89)  [6.08–28.66] |
| Needle | 57 / 344 (16.57)  [13.01–20.86] | 47 / 344 (13.66)  [10.43–17.70] | 14 / 203 (6.90)  [4.15–11.24] |

All data are n/N (%) [Wilson 95% confidence interval]. Edema (non-late) excludes episodes labeled late / delayed / tardive. Late-onset swelling is listed separately. Device was selected at T0 and maintained at maintenance sessions whenever clinically appropriate; categories were not re-assigned. T0, baseline treatment; T1Y, 1-year treatment; T6M, 6-month treatment.
